# Novel associations between parental and newborn cord blood metabolic profiles in the Norwegian Mother, Father and Child Cohort Study

**DOI:** 10.1186/s12916-021-01959-w

**Published:** 2021-04-14

**Authors:** Linn K. L. Øyri, Martin P. Bogsrud, Jacob J. Christensen, Stine M. Ulven, Anne Lise Brantsæter, Kjetil Retterstøl, Hilde K. Brekke, Trond M. Michelsen, Tore Henriksen, Jeanine E. Roeters van Lennep, Per Magnus, Marit B. Veierød, Kirsten B. Holven

**Affiliations:** 1 Department of Nutrition, Institute of Basic Medical Sciences, University of Oslo, P.O. Box 1046, Blindern, 0317 Oslo, Norway; 2 Unit for Cardiac and Cardiovascular Genetics, Department of Medical Genetics, Oslo University Hospital Ullevål, PO Box 4956, Nydalen, 0424 Oslo, Norway; 3 Norwegian National Advisory Unit on Familial Hypercholesterolemia, Department of Endocrinology, Morbid Obesity and Preventive Medicine, Oslo University Hospital Aker, PO Box 4959, Nydalen, 0424 Oslo, Norway; 4 Division of Infection Control and Environmental Health, Section of Environmental Exposure and Epidemiology, Norwegian Institute of Public Health, PO Box 222, Skøyen, 0213 Oslo, Norway; 5 The Lipid Clinic, Department of Endocrinology, Morbid Obesity and Preventive Medicine, Oslo University Hospital Aker, PO Box 4959, Nydalen, 0424 Oslo, Norway; 6 Department of Obstetrics, Oslo University Hospital Rikshospitalet, PO Box 4956, Nydalen, 0424 Oslo, Norway; 7 Institute of Clinical Medicine, Faculty of Medicine, University of Oslo, PO Box 1171, Blindern, 0318 Oslo, Norway; 8 Department of Internal Medicine, Erasmus University Medical Center, Erasmus MC, Dr Molewaterplein 40, 3015 GD Rotterdam, the Netherlands; 9 Centre for Fertility and Health, Norwegian Institute of Public Health, PO Box 222, Skøyen, 0213 Oslo, Norway; 10 Oslo Centre for Biostatistics and Epidemiology, Department of Biostatistics, Institute of Basic Medical Sciences, University of Oslo, PO Box 1122, Blindern, 0317 Oslo, Norway

**Keywords:** MoBa, the Norwegian Mother, Father and Child Cohort Study, MBRN, Medical Birth Registry of Norway, Cholesterol, Metabolic profiling, Cord blood, Sex differences

## Abstract

**Background:**

More than one third of Norwegian women and men between 20 and 40 years of age have elevated cholesterol concentration. Parental metabolic health around conception or during pregnancy may affect the offspring’s cardiovascular disease risk. Lipids are important for fetal development, but the determinants of cord blood lipids have scarcely been studied. We therefore aimed to describe the associations between maternal and paternal peri-pregnancy lipid and metabolic profile and newborn cord blood lipid and metabolic profile.

**Methods:**

This study is based on 710 mother–father–newborn trios from the Norwegian Mother, Father and Child Cohort Study (MoBa) and uses data from the Medical Birth Registry of Norway (MBRN). The sample included in this study consisted of parents with and without self-reported hypercholesterolemia the last 6 months before pregnancy and their partners and newborns. Sixty-four cord blood metabolites detected by nuclear magnetic resonance spectroscopy were analyzed by linear mixed model analyses. The false discovery rate procedure was used to correct for multiple testing.

**Results:**

Among mothers with hypercholesterolemia, maternal and newborn plasma high-density lipoprotein cholesterol, apolipoprotein A1, linoleic acid, docosahexaenoic acid, alanine, glutamine, isoleucine, leucine, valine, creatinine, and particle concentration of medium high-density lipoprotein were significantly positively associated (0.001 ≤ *q* ≤ 0.09). Among mothers without hypercholesterolemia, maternal and newborn linoleic acid, valine, tyrosine, citrate, creatinine, high-density lipoprotein size, and particle concentration of small high-density lipoprotein were significantly positively associated (0.02 ≤ *q* ≤ 0.08). Among fathers with hypercholesterolemia, paternal and newborn ratio of apolipoprotein B to apolipoprotein A1 were significantly positively associated (*q* = 0.04). Among fathers without hypercholesterolemia, no significant associations were found between paternal and newborn metabolites. Sex differences were found for many cord blood lipids.

**Conclusions:**

Maternal and paternal metabolites and newborn sex were associated with several cord blood metabolites. This may potentially affect the offspring’s long-term cardiovascular disease risk.

## Background

More than one third of Norwegian women and men between 20 and 40 years of age have elevated cholesterol concentration [1]. Parental metabolic health around conception or during pregnancy [2, 3] and offspring lifelong cholesterol exposure [4] are increasingly recognized as determinants of the offspring’s cardiovascular disease risk. Lipids required for fetal growth and development are supplied from either maternal blood or endogenous synthesis in the placenta or in the fetus itself [5–9]. Circulating fat and cholesterol are mainly transported by lipoproteins, such as low-density lipoprotein (LDL) and high-density lipoprotein (HDL) [6]. Both the ratio of LDL to HDL cholesterol and the total cholesterol concentration are considerably lower in full-term newborns than in adults [10, 11]. The fetus depends on maternal supply of cholesterol during the first weeks of gestation. Around mid-pregnancy, the fetus establishes its own production of cholesterol [7]. The placenta also supplies the fetus with cholesterol in the last half of pregnancy [9], however in unknown amounts [5]. Studies have found positive associations between maternal early pregnancy and both fetal [12] and offspring childhood [13] cholesterol concentrations. However, regarding the association between maternal gestational and full-term newborn cord blood cholesterol concentration, both positive and no associations have been published [9, 10]. Additionally, the association between paternal and newborn cord blood cholesterol concentration has to our knowledge never been published.

The determinants of the newborn lipid profile are incompletely characterized. We hypothesized that cord blood lipid profile may be related to parental lipid profile, but also to newborn sex [6, 11] and birth weight [6]. By including parents with and without hypercholesterolemia, we tested the hypothesis by exploring the associations between maternal and paternal peri-pregnancy lipid and metabolic profile and newborn cord blood lipid and metabolic profile.

## Methods

### Subjects and study design

The Norwegian Mother, Father and Child Cohort Study (MoBa) is a population-based pregnancy cohort study conducted by the Norwegian Institute of Public Health [14]. Participants were recruited from all over Norway from 1999 to 2008. The women consented to participation in 41% of the invited pregnancies. The cohort now includes 114,500 children, 95,200 mothers, and 75,200 fathers. More than 90% of the women were born in Norway. Invited participants were asked to provide blood and urine samples at baseline and answer questionnaires at regular intervals during pregnancy and after birth.

The sample included in this study consisted of mothers with self-reported hypercholesterolemia or use of lipid-lowering treatment (LLT) the last 6 months before pregnancy and their partners and newborns, and fathers with self-reported use of LLT the last 6 months before pregnancy and their partners and newborns. We had no information regarding paternal self-reported hypercholesterolemia. To simplify readability, both the mothers and the fathers are denoted as with or without hypercholesterolemia. The mothers discontinued LLT before gestational week (GW) 9, while we assumed that the fathers continued using LLT at conception and during the pregnancy. The inclusion criteria were designed to ensure a wide range in measured parental cholesterol concentrations, as it includes both mothers and fathers with and without self-reported hypercholesterolemia.

We included data from four self-reported questionnaires answered around GW 17 (questionnaire 1 for mothers and fathers’ questionnaire), GW 22 (questionnaire 2), and 6 months after birth (questionnaire 4). Questionnaire 1, fathers’ questionnaire, and questionnaire 4 were general questionnaires covering lifestyle, background, illness, and health-related factors. Questionnaire 2 was a semi-quantitative food frequency questionnaire, in which women reported their dietary habits during the first half of the pregnancy. Pregnancy and birth records from the Medical Birth Registry of Norway (MBRN) are linked to the MoBa database using the unique personal identification number assigned to all residents in Norway [15]. The MBRN is a national health registry containing information about all births in Norway. The current study is based on version 11 of the quality-assured data files released for research in September 2018.

**Fig. 1:**
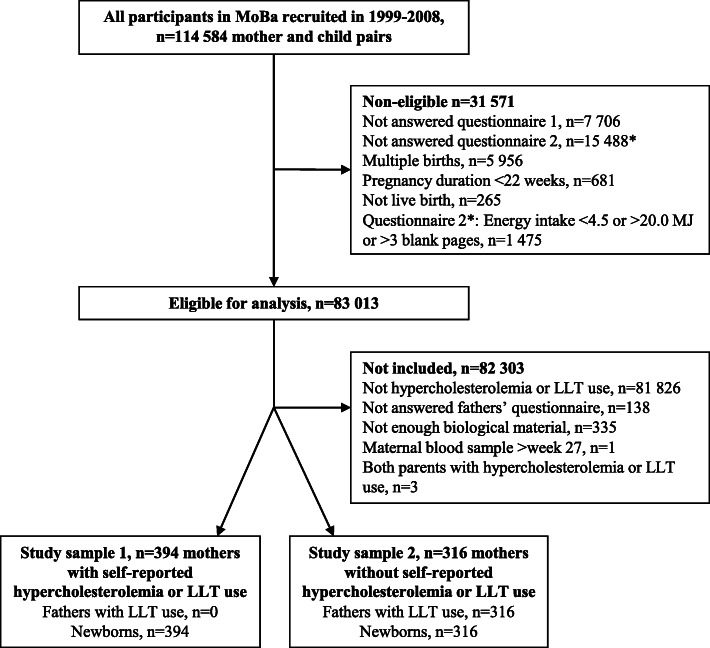
Flow chart of the study sample from the Norwegian Mother, Father and Child Cohort Study. MJ, mega joule; LLT, lipid-lowering treatment. Asterisk indicates questionnaire 2 was included from March 2002

Mothers who did not answer questionnaires 1 or 2; who had a multiple pregnancy, a miscarriage (before GW 22), or stillbirth; or who had an invalid energy intake were not eligible (Fig. 1). We only analyzed blood samples from complete trios where the father had answered his questionnaire. One of the mothers who took the blood sample after GW 27 (in GW 40) was excluded due to the expected gradual increase in maternal cholesterol concentrations during pregnancy [16, 17]. Finally, three trios were excluded as both parents were hypercholesterolemic. This resulted in a total study sample of 710 mother–father–newborn trios, of which 31 mothers participated twice.

### Covariates

Parental age (years), newborn sex (female/male), birth weight (kg), body length (cm), and gestational age (days) were obtained from MBRN. Maternal pre-pregnancy and paternal peri-pregnancy body weight (kg), parental height (cm), education (< 12, 12, 13–16, ≥ 17 years), peri-pregnancy smoking (yes/no), maternal peri-pregnancy blood pressure (mmHg), physical activity level (four categories), and pre-pregnancy hypercholesterolemia (yes/no) were obtained from questionnaire 1. Parental BMI (kg/m^2^) was calculated from questionnaire 1 and gestational weight gain (kg) from questionnaires 1 and 4. Parental self-reported history of cardiovascular disease (yes/no) and use of LLT (yes/no) were obtained from questionnaire 1 and the fathers’ questionnaire. We included the following categories for LLT: HMG-CoA reductase inhibitors (C10A A), bile acid sequestrants (C10A C), other lipid-modifying agents (mainly ezetimibe) (C10A X09), and HMG-CoA reductase inhibitors combined with other LLTs (C10B A). Maternal dietary intake of macronutrients during pregnancy was obtained from questionnaire 2. Maternal age < 17 years was coded as 16 years and > 45 years as 46 years, while paternal age < 18 years was coded as 17 years and > 59 years as 60 years to fulfill data privacy regulations. Birth weights < 500 g and > 6500 g and lengths < 25 cm and > 75 cm were replaced with missing. Parental body weight < 35 kg and > 200 kg and height < 140 cm were replaced with missing. Gestational weight loss ≥ 50 kg and weight gain ≥ 50 kg were replaced with missing.

### Blood samples

Non-fasting blood samples were obtained from both parents during pregnancy (around GW 19) and from the newborn umbilical cord at birth. The processing of the samples is described elsewhere [18]. We measured 148 metabolic biomarkers in EDTA plasma samples by nuclear magnetic resonance spectroscopy at the accredited laboratory Nightingale Health in Finland. Lipid concentration and composition of 14 lipoprotein subclass particles, and among others amino acids, glycolysis measures, ketone bodies, and fatty acids were measured [19]. Only 78 metabolites were detectable in the cord blood. The concentration of phospholipids, cholesterol, cholesteryl esters, free cholesterol, and triglycerides in the 14 lipoprotein subclass particles were not detectable in the cord blood. We included the particle concentration, and not the total lipid concentration, of the 14 lipoprotein subclasses in the statistical analyses. Plasma glucose concentrations of zero were replaced with missing as it is biologically impossible.

### Statistics

Data are presented as mean (standard deviation, SD) or median (interquartile range, IQR) for continuous variables and frequency (%) for categorical variables. The mothers who participated twice are represented with the first pregnancy in subject characteristics, but twice in the main analyses. A kernel density plot is used to visualize the cholesterol distribution in subgroups. In the main analyses, all metabolites of fathers using LLT were LLT corrected, i.e., divided by the metabolite-specific effect of statin treatment relative to placebo found in a study including more than 5000 subjects (Additional file 1 [20]). This was not done for the mothers as they terminated the use of LLT before GW 9, and the effect of previous LLT use was absent in GW 19 [21]. The main analyses were performed in two subgroups including (1) mothers with and fathers without hypercholesterolemia and (2) mothers without and fathers with hypercholesterolemia. Linear mixed model analyses were performed for all cord blood metabolites, except all lipoprotein subclasses. Variables were log_e_ transformed when model assumptions were not fulfilled. Due to many zero values for the largest cord blood lipoprotein subclasses, these variables were dichotomized (≤ or > median) and analyzed by generalized linear mixed model analyses. Newborn sex, birth weight, parental metabolite concentration, BMI, age, and smoking were included in the models based on previous knowledge and directed acyclic graphs (Additional file 2). Gestational age was not included due to a small range and high correlation with birth weight (*r* = 0.6). We performed *p*-value adjustments for multiple testing by the Benjamini−Hochberg false discovery rate (FDR) procedure to correct for the number of metabolites tested (*n* = 64) for all variables in the linear and generalized linear mixed model analyses, and FDR *q*-values from the multivariable analyses are presented in the results section. FDR *q*-values < 0.10 were considered significant. We summarized the results from the multivariable linear mixed model and generalized linear mixed model analyses in a heatmap presenting standardized *β* coefficients and log-odds ratios, and a forest plot presenting estimated *β* coefficients and log-odds ratios, respectively. Univariable and multivariable estimated *β* coefficients (log-odds ratios from generalized linear mixed model analyses) with 95% confidence intervals (CIs), *p*-values, and FDR *q*-values are presented in an additional file. We conducted sensitivity analyses with and without birth weight, gestational age, maternal and paternal education, BMI, age, smoking, correction for paternal use of LLT, and all subjects included. The statistical analyses were performed in R version 3.6.1 with RStudio IDE version 1.3.1073 [22].

## Results

### Subjects

Subject characteristics of the 679 mother–father–newborn trios are shown in Table 1. Fifty percent of the newborns were female, and the mean (SD) birth weight and length were 3.5 (0.5) kg and 50.0 (2.1) cm in females and 3.7 (0.5) kg and 50.9 (2.1) cm in males, respectively. The mothers included in the current study were slightly older (mean 31.7 vs 30.2 years) and had a higher pre-pregnancy BMI (25.2 vs 24.1 kg/m^2^) than the remaining MoBa participants; so were the included fathers (34.7 vs 32.8 years, and 27.0 vs 25.9 kg/m^2^, respectively) (Additional file 3). Proportions smoking during pregnancy were similar in the mothers in the current sample and the remaining MoBa participants (7.4 vs 8.0%), while lower for fathers (16.0 vs 20.1%). Moreover, fathers with hypercholesterolemia were older (36.9 vs 33.0 years) and had a higher prevalence of cardiovascular disease (19.5 vs 0.5%) than fathers without hypercholesterolemia. Of the 316 fathers using LLT, 298 used only statins (C10A A), eight used only bile acid sequestrants (C10A C), two used statins and bile acid sequestrants, and eight used statins and ezetimibe (C10A X09). Maternal physical activity level and dietary intake of macronutrients are shown in Table 2. The prevalence of cesarean section was 14% in mothers with and 12% in mothers without hypercholesterolemia. The prevalence of gestational diabetes, gestational hypertension, preeclampsia, and newborn congenital malformations was ≤ 5% in both mothers with and without hypercholesterolemia.

**Table 1:** Subject characteristics

	Mothers				Fathers				Newborns	
	With hypercholesterolemia	n*	Without hypercholesterolemia	n*	With hypercholesterolemia	n*	Without hypercholesterolemia	n*	All	n*
**Age (years), mean (SD)**	31.2 (4.5)	376	32.3 (4.4)	303	36.9 (6.6)	303	33.0 (5.1)	376	0	679
**Female,** ***n*** **(%)**	–		–		–		–		341 (50.2)	679
Body weight** (kg), mean (SD)	70.7 (14.9)	373	71.1 (14.0)	294	91.0 (14.3)	293	86.3 (12.6)	369	3.6 (0.5)	679
**Height (cm), mean (SD)**	167 (6)	375	168 (6)	301	180 (7)	297	182 (7)	371	51 (2)	652
BMI*** (kg/m^**2**^**), mean (SD)**	25.2 (5.1)	373	25.2 (4.9)	294	28.0 (4.0)	292	26.2 (3.5)	369	–	
**Gestational weight gain (kg), mean (SD)**	14.3 (6.4)	334	14.4 (6.1)	251	–		–		–	
**Gestational age (days), median (IQR)**	281 (273–287)	376	281 (274–287)	303	–		–		–	
**Education (years),** ***n*** **(%)**										
**< 12**	24 (6.6)	366	22 (7.5)	293	25 (8.7)	289	33 (9.1)	362	–	
**12**	87 (23.8)	366	80 (27.3)	293	108 (37.4)	289	123 (34.0)	362	–	
**13–16**	151 (41.3)	366	123 (42.0)	293	90 (31.1)	289	110 (30.4)	362	–	
≥ **17**	104 (28.4)	366	68 (23.2)	293	66 (22.8)	289	96 (26.5)	362	–	
**Smoking during pregnancy,** ***n*** **(%)**	28 (7.5)	375	22 (7.3)	303	46 (15.3)	300	62 (16.6)	374	–	
**SPB (mmHg), mean (SD)**	116 (12)	344	115 (12)	262	–		–		–	
**DBP (mmHg), mean (SD)**	71 (9)	344	70 (9)	262	–		–		–	
**CVD,** ***n*** **(%)**	3 (0.8)	376	3 (1.0)	303	59 (19.5)	303	2 (0.5)	376	–	
**LLT,** ***n*** **(%)**	48 (12.8)	376	0		303 (100)	303	0		–	
**Total cholesterol (mmol/l), mean (SD)**	6.1 (1.2)	375	4.8 (0.9)	302	3.8 (1.0)	303	3.9 (0.9)	375	1.4 (0.5)	674
**LDL cholesterol (mmol/l), mean (SD)**	2.3 (0.7)	375	1.6 (0.5)	302	1.4 (0.6)	303	1.5 (0.5)	375	0.3 (0.2)	674
**HDL cholesterol (mmol/l), mean (SD)**	1.9 (0.3)	375	1.8 (0.3)	302	1.1 (0.2)	303	1.1 (0.2)	375	0.8 (0.2)	674

*SD*, standard deviation; *IQR*, interquartile range; *SPB*, peri-pregnancy systolic blood pressure; *DBP*, peri-pregnancy diastolic blood pressure; *CVD*, cardiovascular disease; *LLT*, pre-pregnancy lipid-lowering treatment; *LDL*, low-density lipoprotein; *HDL*, high-density lipoprotein

*Includes first participation only (31 mothers participated twice in our sample); **body weight is birth weight for newborns; ***BMI is pre-pregnancy for mothers and peri-pregnancy for fathers (at completion of questionnaire 1)

**Table 2:** Maternal physical activity level and dietary intake during pregnancy

	Mothers			
	With hypercholesterolemia	n*	Without hypercholesterolemia	n*
**Physical activity level,** ***n*** **(%)**				
Never	45 (22.2)	203	40 (23.8)	168
1–3 times/month	94 (46.3)	203	93 (55.4)	168
1–2 times/week	48 (23.6)	203	28 (16.7)	168
≥ 3 times/week	16 (7.9)	203	7 (4.2)	168
**Dietary intake, mean (SD)**				
Energy (MJ)	9.4 (2.3)	376	9.6 (2.5)	303
Fat (E%)	31.1 (4.8)	376	31.2 (5.1)	303
SFA (E%)	11.7 (2.0)	376	11.9 (2.2)	303
MUFA (E%)	10.1 (2.0)	376	10.1 (2.0)	303
PUFA (E%)	6.1 (1.8)	376	6.0 (1.8)	303
Protein (E%)	15.5 (2.1)	376	15.7 (2.1)	303
Carbohydrates (E%)	53.2 (4.9)	376	52.8 (5.2)	303
Sugar (E%)	10.1 (4.4)	376	9.9 (4.9)	303

*SD*, standard deviation; *MJ*, mega joule; *E%*, percentage of energy; *SFA*, saturated fatty acids; *MUFA*, monounsaturated fatty acids; *PUFA*, polyunsaturated fatty acids

*Includes first participation only (31 mothers participated twice in our sample)

**Fig. 2:**
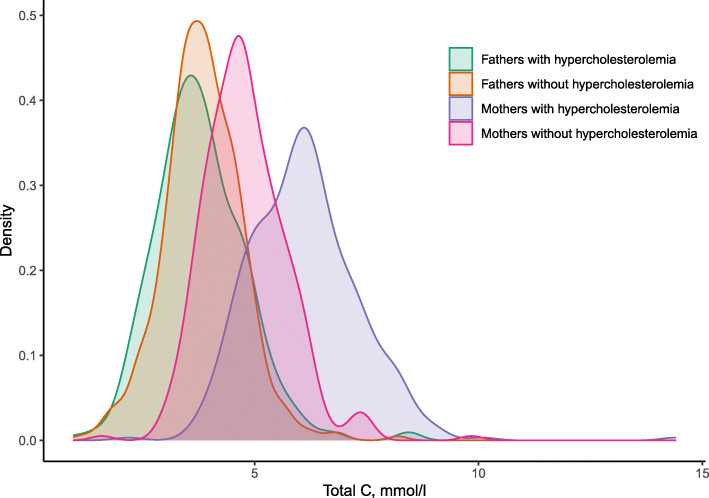
Density plot of distributions of total cholesterol concentrations in the parents. C, cholesterol. Includes first participation only (31 mothers participated twice in our sample)

Blood samples were obtained in mean (SD, min-max) GW 19 (1, 13–27) among the mothers and 19 (2, 13–41) among the fathers. Mothers with hypercholesterolemia had higher total cholesterol concentration (mean [SD] 6.1 [1.2] vs 4.8 [0.9] mmol/l) than mothers without hypercholesterolemia. However, fathers with hypercholesterolemia did not have higher total cholesterol concentration than fathers without hypercholesterolemia (3.8 [1.0] vs 3.9 [0.9] mmol/l, not LLT corrected) (Fig. 2 and Table 1). The concentrations of female and male newborn and parental metabolites are shown in Additional file 1.

### Associations between parental and newborn metabolic profile

**Fig. 3:**
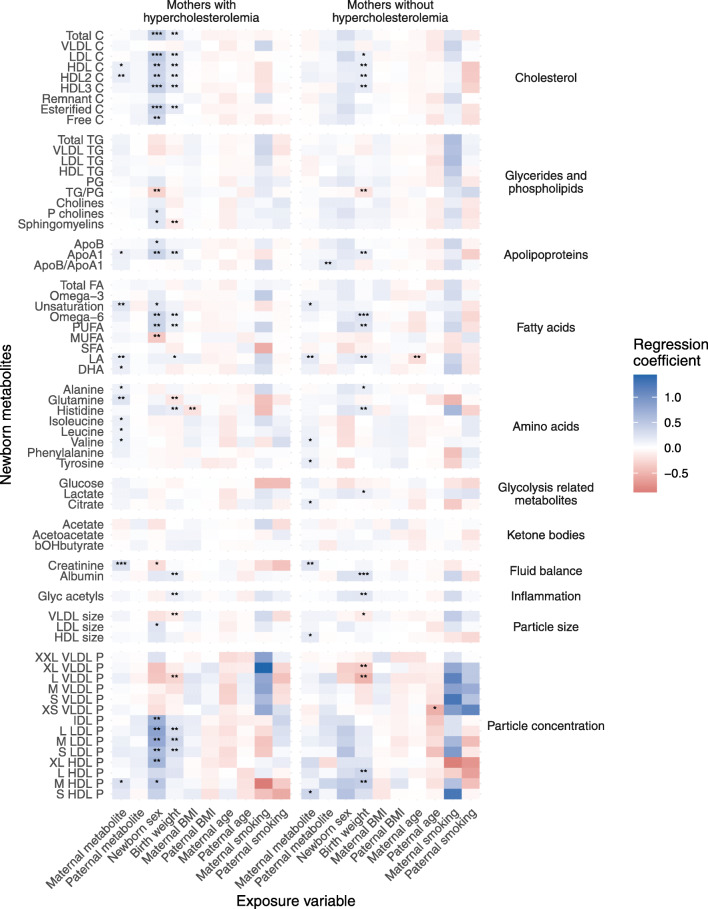
Heatmap of the associations between parental and newborn exposures and newborn metabolites. Results are presented as standardized *β* coefficients from multivariable linear mixed model analyses, but as log-odds ratios from multivariable generalized linear mixed model analyses for lipoprotein particle concentrations. C, cholesterol; VLDL, very low-density lipoprotein; LDL, low-density lipoprotein; HDL, high-density lipoprotein; TG, triglycerides; PG, phosphoglycerides; P cholines, phosphatidylcholines; Apo, apolipoprotein; FA, fatty acid; PUFA, polyunsaturated fatty acid; MUFA, monounsaturated fatty acid; SFA, saturated fatty acid; LA, linoleic acid; DHA, docosahexaenoic acid; bOHbutyrate, β-hydroxybutyrate; glyc, glycoprotein; P, particle concentration; IDL, intermediate-density lipoprotein. Sex, females vs males; smoking, yes vs no; FDR, adjusted for false discovery rate. ***q_FDR_ < 0.001; **0.001 ≤ *q*_FDR_ < 0.05; *0.05 ≤ *q*_FDR_ < 0.10

Among mothers with hypercholesterolemia, maternal and newborn plasma HDL and HDL2 cholesterol, apolipoprotein (Apo)A1, degree of fatty acid unsaturation, linoleic acid (%), docosahexaenoic acid (%), alanine, glutamine, isoleucine, leucine, valine, creatinine, and particle concentration of medium HDL were significantly positively associated (multivariable 0.001 ≤ *q* ≤ 0.09, Fig. 3 and Additional file 4). Among mothers without hypercholesterolemia, maternal and newborn degree of fatty acid unsaturation, linoleic acid (%), valine, tyrosine, citrate, creatinine, HDL size, and particle concentration of small HDL were significantly positively associated (multivariable 0.02 ≤ *q* ≤ 0.08). Among fathers with hypercholesterolemia, paternal LLT corrected and newborn ratio of ApoB to ApoA1 were significantly positively associated (multivariable *q* = 0.04). Among fathers without hypercholesterolemia, no significant associations were found between paternal and newborn metabolites.

### Associations between other possible determinants and newborn metabolic profile

**Fig. 4:**
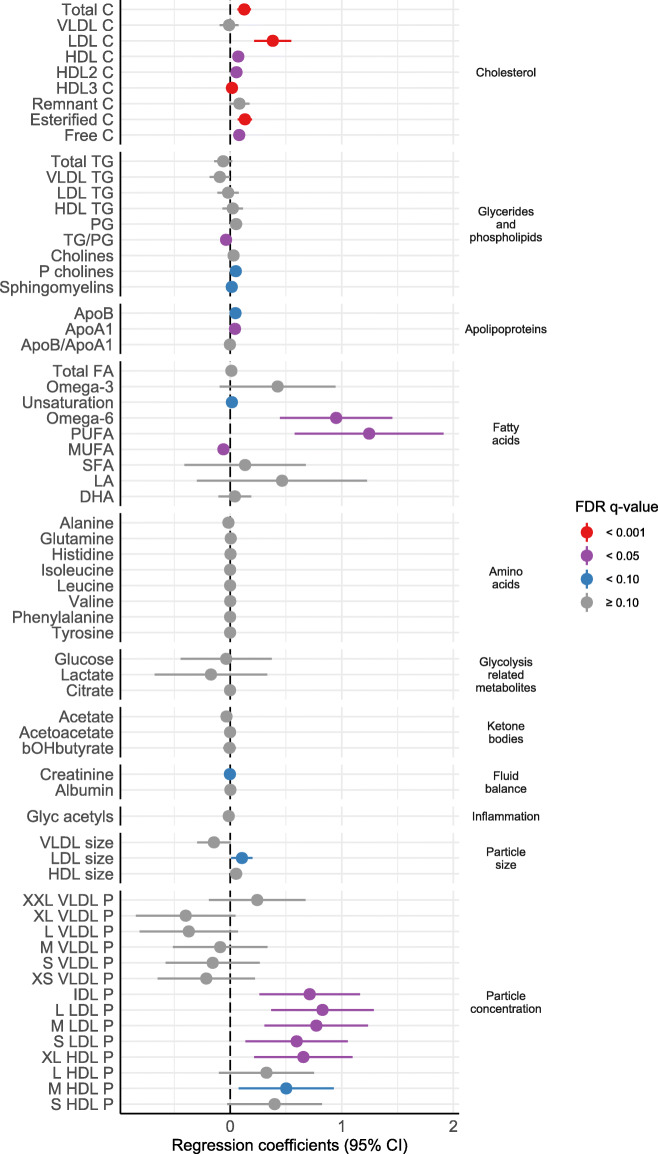
Forest plot of sex differences in cord blood metabolites in newborns of mothers with hypercholesterolemia. Results are presented as estimated *β* coefficients from multivariable linear mixed model analyses, but as log-odds ratios from multivariable generalized linear mixed model analyses for lipoprotein particle concentrations, with 95% confidence intervals (CIs), and FDR *q*-values (adjusted for false discovery rate) for females compared to males (vertical zero-line). C, cholesterol; VLDL, very low-density lipoprotein; LDL, low-density lipoprotein; HDL, high-density lipoprotein; TG, triglycerides; PG, phosphoglycerides; P cholines, phosphatidylcholines; Apo, apolipoprotein; FA, fatty acid; PUFA, polyunsaturated fatty acid; MUFA, monounsaturated fatty acid; SFA, saturated fatty acid; LA, linoleic acid; DHA, docosahexaenoic acid; bOHbutyrate, β-hydroxybutyrate; Glyc, glycoprotein; P, particle concentration; IDL, intermediate-density lipoprotein

Among newborns of mothers with hypercholesterolemia, female compared to male newborns had significantly higher plasma concentrations of several cholesterol metabolites, phosphatidylcholines, sphingomyelins, ApoB, ApoA1, degree of fatty acid unsaturation, omega-6 fatty acids (%), polyunsaturated fatty acids (%), LDL size, and particle concentration of intermediate-density lipoprotein, LDL, and HDL subclasses (multivariable 0.001 ≤ *q* ≤ 0.098, Figs. 3 and 4 and Additional files 1 and 4). Female newborns had a significantly lower concentration of ratio of triglycerides to phosphoglycerides, monounsaturated fatty acids (%), and creatinine than male newborns (multivariable 0.003 ≤ *q* ≤ 0.06). Among newborns of mothers without hypercholesterolemia, no significant sex differences were found.

We found several significant associations between birth weight and newborn metabolites (multivariable 0.001 ≤ *q* ≤ 0.09, Fig. 3 and Additional file 4). There were few significant associations between parental BMI, age, and smoking and newborn metabolites. Sensitivity analyses with and without parental education, BMI, age, smoking, correction for paternal use of LLT, newborn birth weight, and gestational age gave similar results for parent–newborn associations and sex differences as presented in Fig. 3 (results not shown). Results of an analysis including all subjects are presented in Additional file 5.

## Discussion

We found significant positive associations between maternal and newborn HDL cholesterol and subclasses, long-chain polyunsaturated fatty acids, and several amino acids, and between paternal and newborn ratio of ApoB to ApoA1. Furthermore, we found that female newborns had higher concentrations of many cholesterol metabolites and polyunsaturated fatty acids, but lower concentrations of monounsaturated fatty acids than male newborns.

### Associations between parental and newborn metabolic profiles

During pregnancy, several maternal metabolic alterations occur, such as an increase in the circulating concentration of cholesterol, triglycerides, phospholipids, and fatty acids [16]. It is well known that the placenta is supplied with cholesterol from lipoproteins such as LDL and HDL in the maternal circulation [5]. In a recent Norwegian study, fetal uptake of cholesterol from the umbilical circulation was positively correlated to uteroplacental uptake of cholesterol in the maternal circulation at term [9]. Furthermore, newborns lacking the ability to synthesize cholesterol have measurable amounts of cholesterol in the circulation [5]. These findings indicate that there is a fetal uptake of cholesterol released from the placenta at term and that the placenta modulates maternal–fetal cholesterol transfer. The mechanisms and significance of cholesterol transport from the placenta to the fetal circulation are unclear. It is hypothesized that placental cholesterol enter the fetal circulation via the ATP-binding cassette transporters ABCA1 and ABCG1 and are thereafter embedded in (nascent) HDL particles [8]. The placenta may also secrete apoB containing particles to the fetal circulation [23].

We found a significant association between maternal and newborn HDL cholesterol and subclasses, but not LDL cholesterol and subclasses. This association might be explained by fetal HDL being the major acceptor of cholesterol from the placenta [6, 8]. Furthermore, the association between maternal and newborn HDL cholesterol might be more clinically relevant than LDL cholesterol, as newborn HDL contributed more to the total cholesterol concentration in the current study.

Similar genes in mother and newborn may explain parts of the association between maternal and newborn cholesterol concentration [10]. In a paper using data from adults in the Framingham study, 12% of the variance in total cholesterol could be explained by single nucleotide polymorphisms [24]. In the same study, a significant association was found between maternal pre-pregnancy and adult offspring LDL cholesterol beyond measured lifestyle, anthropometric factors, and known single nucleotide polymorphisms; the authors suggested a role of epigenetics [25]. It is tempting to speculate if the potential maternal–newborn association related to genetics is masked by the low LDL cholesterol concentrations in the cord blood and the altered maternal metabolism and lifestyle during pregnancy.

A positive association between total cholesterol concentration of fathers and offspring aged 0.5 to 4 years has been found previously [26]. The association between paternal and newborn cord blood lipids has to our knowledge never been published. Fathers with hypercholesterolemia in the current study had similar not LLT corrected, but higher LLT corrected total cholesterol concentration than fathers without hypercholesterolemia. The association between hypercholesterolemic paternal and newborn ratio of ApoB to ApoA1 was similar both when correcting and not correcting for LLT use. This association might be explained by genetics [24], shared lifestyle between cohabiting parents, and paternal pre-conception lifestyle. Both human, animal, and cell studies indicate that paternal diet can affect sperm quality and epigenetic status and subsequently affect offspring health [3].

### Associations between newborn sex and metabolic profile

Among newborns of mothers with hypercholesterolemia, we found several sex differences in the cord blood lipid profile, revealing that females had significantly higher concentrations of LDL and HDL cholesterol and subclasses than males. Among newborns of mothers without hypercholesterolemia, there were similar but non-significant trends possibly related to lower total cholesterol concentration among their mothers. Previous studies have shown that newborn girls have higher LDL and HDL cholesterol [6, 11], but no study has found sex differences in such a wide range of metabolic markers as the present study. Higher LDL cholesterol concentration in girls than in boys has also been shown from 0 to 19 years of age in both normocholesterolemic and hypercholesterolemic populations [27, 28].

There could be several physiological mechanisms behind the sex differences. We adjusted for birth weight, which was lower among female than male newborns. However, others have found that female newborns have different body composition compared to male newborns, with higher body fat percentage [29] and leptin concentrations [30] in female newborns. Positive associations between leptin and body fat percentage and total and LDL cholesterol have been reported in adolescents previously [31]. Furthermore, cholesterol is essential for the synthesis of steroid hormones [5]. Female estrogen has been linked to higher HDL, but lower LDL cholesterol concentrations in adults [32]. Finally, there may be differences in placental lipid transport in pregnancies with female compared to male fetuses [33].

### Clinical relevance

The clinical relevance of the associations discussed above is unclear. Female fetuses have shown better adaptation to an adverse intrauterine environment and lower prevalence of pregnancy complications than male fetuses [33]. We and others [6] have found a positive association between newborn cholesterol concentration and birth weight, and one may speculate if this relation is causal as cholesterol is used for cell growth. Furthermore, the periconceptional and in utero period has emerged as critical regarding parental influence on offspring long-term risk of diseases [2, 3]. It is not known whether LDL cholesterol in the cord blood is suitable to identify individuals at risk for hypercholesterolemia and subsequent cardiovascular disease later in life, due to the low concentrations and large dependence of factors such as birth weight [6]. Newborn LDL concentrations are probably not suitable to identify subjects with the genetic disorder familial hypercholesterolemia [34]. However, maternal gestational hypercholesterolemia has been associated with increased fetal and childhood fatty streak formation [12, 35], indicating that the process of atherosclerosis may begin already in fetal life.

### Strengths and limitations

A major strength of the current study is the large number of population-based blood samples from the well-established MoBa study linked to the national MBRN providing detailed pregnancy data. We measured a comprehensive metabolic profile in mother–father–newborn trios at an accredited laboratory and have detailed information about a wide range of potential confounders. Weaknesses include the reliance on self-reported variables from the MoBa questionnaires: parental weight, height, smoking, and hypercholesterolemia and use of LLT for selection of the study sample. However, a substantial agreement between self-reported and measured BMI was found in a large cohort of Norwegian females [36]. Smoking during pregnancy has been validated [37]. We had no information about the use of drugs beyond LLT. Total cholesterol has been analyzed previously in 891 randomly selected pregnant mothers in MoBa [38], and the concentration was median 5.4 mmol/l as compared to 6.1 and 4.8 mmol/l in women with and without hypercholesterolemia in the current study. In contrast, total cholesterol concentration has been found to be mean 9.1 mmol/l in GW 17–20 in Norwegian women with familial hypercholesterolemia [39]; thus, we assume that subjects without familial hypercholesterolemia are dominating in our sample.

The blood samples were non-fasting; however, minimal postprandial changes have previously been observed in total cholesterol and triglycerides [40]. Ninety percent of the plasma samples were stored at room temperature for 3 days or less after blood sampling [18]. This probably had a minor effect on cholesterol and triglycerides, but may have affected the absolute concentration of fatty acids [41, 42]. Moreover, parental blood samples were drawn mid-pregnancy while newborn cord blood was drawn at birth. Maternal total cholesterol has been shown to increase linearly from the second to the ninth gestational month [17]. The percentage increase seems to be independent of pre-pregnancy total cholesterol concentration, as it has been shown to be similar between women with and without familial hypercholesterolemia [39]. We did not include maternal blood samples drawn immediately after birth due to the assumed large changes in plasma metabolites at that time [43]. Finally, of those invited to the whole MoBa study, 41% agreed to participate, and they have been reported to be older, better educated, and more frequently non-smokers than those who declined participation [44], which is in agreement with the trend in other epidemiological studies [45]. Thus, one cannot rule out selection bias.

## Conclusions

In the current study, we found significant positive associations between maternal and newborn HDL cholesterol and subclasses, and for the first time between paternal and newborn ratio of ApoB to ApoA1. Furthermore, we found that girls had higher concentrations of LDL and HDL cholesterol and subclasses than boys already at birth. This may potentially affect the offspring’s long-term cardiovascular disease risk; however, further research is warranted to elucidate molecular mechanisms and clinical significance.

## Supplementary Information

**Additional file 1.** Concentration of plasma metabolites in female and male newborns (cord blood), mothers and fathers.

**Additional file 2.** Directed acyclic graphs of the relation between parental and newborn exposures and newborn metabolites.

**Additional file 3.** Characteristics of subjects who were included and not included.

**Additional file 4.** Results from mixed model analyses for the association between parental and newborn exposures and newborn metabolites.

**Additional file 5.** Heatmap of the associations between parental and newborn exposures and newborn metabolites including all subjects.

## Data Availability

The consent given by the participants does not open for storage of data on an individual level in repositories or journals. Researchers who want access to data sets for replication should submit an application to datatilgang@fhi.no. Access to data sets requires approval from the Regional Committee for Medical Research Ethics in Norway and a formal contract with MoBa.

## Abbreviations

Apo: apolipoprotein
FDR: False discovery rate
GW: Gestational week
HDL: High-density lipoprotein
LDL: Low-density lipoprotein
LLT: Lipid-lowering treatment
MBRN: Medical Birth Registry of Norway
MoBa: The Norwegian Mother, Father and Child Cohort Study
