# Supplementary material for: Novel associations between parental and newborn cord blood metabolic profiles in the Norwegian Mother, Father and Child Cohort Study
Source: BMC Med. 2021 Apr 14;19:91. doi: 10.1186/s12916-021-01959-w (PMC8045233; doi:10.1186/s12916-021-01959-w)
Supplement: Supplementary file 1 — Additional file 1. Concentration of plasma metabolites in female and male newborns (cord blood), mothers and fathers. [file 12916_2021_1959_MOESM1_ESM.pdf]

Additional File 1. Concentration of plasma metabolites in female and male newborns (cord blood), mothers and fathers.

		Newborns								Mothers								Fathers								
		Female				Male				With hypercholesterolemia				Without hypercholesterolemia				With hypercholesterolemia*				Without hypercholesterolemia				Correction factor*
**Variable**	**Unit**	**n**	**p25**	**p50**	**p75**	**n**	**p25**	**p50**	**p75**	**n**	**p25**	**p50**	**p75**	**n**	**p25**	**p50**	**p75**	**n**	**p25**	**p50**	**p75**	**n**	**p25**	**p50**	**p75**	
**Total C**	mmol/l	358	1.16	1.43	1.70	347	1.05	1.30	1.58	393	5.26	6.05	6.80	315	4.15	4.70	5.32	316	4.06	4.82	5.72	393	3.30	3.83	4.39	0.773
**VLDL C**	mmol/l	358	0.14	0.18	0.23	347	0.14	0.18	0.24	393	0.72	0.91	1.13	315	0.50	0.64	0.82	316	0.67	0.85	1.08	393	0.47	0.62	0.78	0.739
**LDL C**	mmol/l	358	0.15	0.25	0.39	347	0.09	0.20	0.33	393	1.83	2.24	2.66	315	1.27	1.55	1.83	316	1.63	2.12	2.72	393	1.17	1.44	1.78	0.651
**HDL C**	mmol/l	358	0.71	0.81	0.93	347	0.65	0.78	0.90	393	1.64	1.85	2.07	315	1.62	1.81	2.00	316	0.99	1.11	1.24	393	0.99	1.11	1.25	0.992
**LDL C/HDL C**	ratio	358	0.19	0.29	0.46	347	0.12	0.26	0.41	393	0.96	1.20	1.49	315	0.70	0.87	1.03	316	1.42	1.91	2.42	393	1.00	1.28	1.63	
**HDL2 C**	mmol/l	358	0.28	0.37	0.47	347	0.24	0.34	0.45	393	1.08	1.27	1.48	315	1.09	1.25	1.43	316	0.48	0.58	0.70	393	0.52	0.64	0.77	1.065
**HDL3 C**	mmol/l	358	0.42	0.44	0.46	347	0.40	0.43	0.45	393	0.56	0.57	0.59	315	0.53	0.55	0.57	316	0.53	0.55	0.57	393	0.46	0.48	0.49	0.869
**Remnant C**	mmol/l	358	0.25	0.34	0.44	347	0.24	0.32	0.41	393	1.55	1.86	2.23	315	1.13	1.33	1.60	316	1.35	1.69	2.10	393	0.96	1.24	1.49	0.720
**Esterified C**	mmol/l	358	0.77	0.96	1.18	346	0.70	0.88	1.09	391	3.71	4.28	4.79	316	2.91	3.31	3.71	316	2.81	3.35	3.98	393	2.32	2.69	3.07	0.779
**Free C**	mmol/l	358	0.39	0.46	0.55	346	0.37	0.43	0.50	391	1.56	1.79	2.00	316	1.25	1.40	1.57	316	1.25	1.48	1.73	393	0.99	1.14	1.31	0.759
**Total TG**	mmol/l	358	0.25	0.31	0.43	347	0.25	0.32	0.44	393	1.17	1.48	1.91	315	0.94	1.16	1.52	316	1.06	1.45	1.86	393	0.84	1.09	1.42	0.823
**VLDL TG**	mmol/l	358	0.13	0.17	0.23	347	0.14	0.18	0.25	393	0.55	0.77	1.07	315	0.44	0.60	0.84	316	0.65	0.96	1.29	393	0.51	0.72	0.96	0.821
**LDL TG**	mmol/l	358	0.04	0.05	0.07	347	0.04	0.05	0.07	393	0.25	0.30	0.36	315	0.20	0.23	0.28	316	0.16	0.20	0.25	393	0.12	0.15	0.19	0.801
**HDL TG**	mmol/l	358	0.03	0.04	0.06	347	0.03	0.04	0.05	393	0.19	0.22	0.25	315	0.16	0.19	0.22	316	0.13	0.16	0.19	393	0.11	0.13	0.16	0.856
**PG**	mmol/l	357	0.71	0.83	0.99	345	0.68	0.80	0.93	391	2.38	2.62	2.89	316	2.02	2.21	2.52	316	1.47	1.68	1.96	393	1.27	1.49	1.68	0.890
**TG/PG**	ratio	357	0.24	0.33	0.42	345	0.25	0.33	0.46	391	0.48	0.57	0.69	316	0.47	0.55	0.63	316	0.56	0.73	0.89	393	0.52	0.65	0.80	0.968
**Cholines**	mmol/l	357	1.15	1.29	1.44	345	1.11	1.24	1.40	391	2.84	3.10	3.40	316	2.43	2.63	2.95	316	1.85	2.08	2.38	393	1.64	1.86	2.06	0.891
**Phosphatidyl-cholines**	mmol/l	357	0.75	0.87	1.02	345	0.73	0.84	0.97	391	2.39	2.64	2.90	316	2.03	2.22	2.52	316	1.53	1.73	1.99	393	1.34	1.53	1.73	0.894
**Sphingo-myelins**	mmol/l	357	0.20	0.24	0.28	345	0.19	0.23	0.26	391	0.53	0.60	0.66	316	0.44	0.49	0.54	316	0.38	0.43	0.52	393	0.34	0.39	0.43	0.861
**ApoB**	g/l	358	0.30	0.34	0.39	347	0.29	0.32	0.37	393	0.94	1.10	1.26	315	0.74	0.84	0.96	316	0.89	1.04	1.22	393	0.68	0.82	0.93	0.772
**ApoA1**	g/l	358	0.85	0.92	1.00	347	0.83	0.90	0.97	393	1.68	1.79	1.91	315	1.59	1.70	1.81	316	1.24	1.33	1.42	393	1.22	1.30	1.38	0.974
**ApoB/ApoA1**	ratio	358	0.32	0.36	0.41	347	0.32	0.36	0.40	393	0.53	0.61	0.70	315	0.43	0.50	0.57	316	0.69	0.79	0.89	393	0.54	0.63	0.72	0.794
**Total FA**	mmol/l	352	3.77	4.34	5.06	337	3.76	4.26	4.87	389	13.70	15.40	17.10	316	11.10	12.50	14.30	315	9.87	11.60	13.90	392	8.09	9.55	11.10	0.846
**Unsaturation**	degree	352	1.23	1.28	1.3	337	1.23	1.27	1.32	389	1.15	1.18	1.20	316	1.14	1.17	1.21	315	1.12	1.18	1.22	392	1.16	1.20	1.24	1.024
**Omega- 3**	%	352	7.2	8.8	10.8	337	6.7	8.5	10.4	389	4.6	5.0	5.5	316	4.4	4.8	5.4	315	4.1	4.7	5.2	392	3.8	4.3	4.8	1.030
**Omega-6**	%	352	24.5	26.5	27.7	337	24.2	25.6	27.2	389	29.2	30.6	31.7	316	29.1	30.4	31.6	315	29.2	31.2	33.5	392	29.7	31.5	33.1	0.982
**PUFA**	%	352	33.6	35.1	37.3	337	32.4	34.2	36.5	389	34.3	35.8	37.2	316	33.8	35.4	36.6	315	33.8	36.0	38.4	392	34.0	36.0	37.6	0.987
**MUFA**	%	352	19.9	22.2	24.6	337	20.7	23.0	25.8	389	26.0	27.1	28.3	316	26.5	27.4	28.6	315	25.9	27.6	29.2	392	25.8	27.0	28.5	1.004
**SFA**	%	352	40.6	42.2	44.0	337	40.4	42.1	44.4	389	36.4	37.2	37.9	316	36.5	37.2	38.1	315	35.4	36.3	37.4	392	35.9	36.9	37.9	1.010
**LA**	%	352	11.2	13.4	15.9	337	10.9	13.1	15.4	389	23.0	24.3	25.6	316	22.3	23.7	25.0	315	22.6	24.8	26.3	392	22.9	24.6	26.0	0.946
**DHA**	%	352	2.5	2.9	3.3	337	2.5	2.8	3.3	389	1.5	1.7	1.9	316	1.4	1.6	1.8	315	1.3	1.5	1.7	392	1.2	1.4	1.6	1.054
**Omega- 3**	mmol/l	352	0.30	0.39	0.50	337	0.28	0.37	0.48	389	0.68	0.77	0.89	316	0.52	0.61	0.71									
**Omega-6**	mmol/l	352	0.97	1.14	1.34	337	0.96	1.09	1.25	389	4.13	4.66	5.18	316	3.37	3.75	4.24									
**PUFA**	mmol/l	352	1.31	1.55	1.80	337	1.24	1.48	1.72	389	4.83	5.43	6.07	316	3.94	4.37	4.96									

**MUFA**	mmol/l	352	0.80	0.97	1.16	337	0.81	1.00	1.19	389	3.61	4.15	4.76	316	2.96	3.41	4.06									
**SFA**	mmol/l	352	1.59	1.85	2.08	337	1.63	1.82	2.03	389	5.02	5.71	6.39	316	4.12	4.62	5.36									
**LA**	mmol/l	352	0.44	0.59	0.76	337	0.43	0.55	0.71	389	3.27	3.69	4.20	316	2.58	2.97	3.42									
**DHA**	mmol/l	352	0.11	0.13	0.16	337	0.10	0.12	0.15	389	0.22	0.26	0.30	316	0.17	0.20	0.24									
**Alanine**	mmol/l	358	0.54	0.65	0.79	347	0.55	0.64	0.77	393	0.35	0.37	0.40	316	0.33	0.36	0.39	316	0.37	0.40	0.44	393	0.35	0.39	0.43	1.009
**Glutamine**	mmol/l	320	0.28	0.33	0.37	296	0.28	0.32	0.36	383	0.27	0.30	0.33	310	0.26	0.30	0.33	292	0.33	0.37	0.42	367	0.35	0.40	0.44	1.007
**Histidine**	mmol/l	357	0.07	0.08	0.09	343	0.07	0.07	0.08	392	0.04	0.04	0.05	316	0.04	0.04	0.05	316	0.05	0.05	0.06	390	0.05	0.06	0.06	0.992
**Isoleucine**	mmol/l	357	0.04	0.05	0.05	347	0.04	0.05	0.05	392	0.04	0.05	0.06	316	0.04	0.04	0.05	316	0.05	0.07	0.08	393	0.05	0.06	0.08	0.972
**Leucine**	mmol/l	358	0.05	0.06	0.07	347	0.05	0.06	0.07	393	0.05	0.06	0.07	316	0.05	0.06	0.07	316	0.07	0.08	0.10	393	0.07	0.08	0.09	1.013
**Valine**	mmol/l	358	0.14	0.16	0.17	347	0.14	0.16	0.18	393	0.11	0.13	0.14	315	0.10	0.12	0.14	316	0.15	0.17	0.20	393	0.15	0.17	0.20	1.016
**Phenylalanine**	mmol/l	355	0.07	0.08	0.09	344	0.07	0.08	0.09	393	0.06	0.06	0.07	316	0.06	0.06	0.07	314	0.05	0.06	0.07	393	0.05	0.06	0.06	1.008
**Tyrosine**	mmol/l	358	0.05	0.06	0.07	344	0.05	0.06	0.07	392	0.03	0.04	0.05	316	0.03	0.04	0.04	316	0.05	0.05	0.06	390	0.04	0.05	0.06	1.005
**Glucose**	mmol/l	316	0.08	0.53	1.91	305	0.09	0.56	2.23	391	3.07	3.48	3.85	316	3.06	3.44	3.78	316	3.26	3.79	4.28	389	3.06	3.59	3.92	1.000
**Lactate**	mmol/l	358	6.50	8.06	9.94	347	6.31	8.01	9.78	393	0.94	1.21	1.71	316	0.90	1.22	1.76	316	0.98	1.29	2.13	393	0.92	1.27	1.93	1.000
**Citrate**	mmol/l	351	0.14	0.15	0.17	340	0.13	0.15	0.17	393	0.10	0.11	0.12	316	0.10	0.11	0.12	316	0.10	0.11	0.12	393	0.10	0.11	0.12	0.991
**Acetate**	mmol/l	354	0.03	0.03	0.04	345	0.03	0.04	0.04	393	0.03	0.03	0.04	316	0.03	0.03	0.03	316	0.03	0.03	0.04	393	0.03	0.03	0.04	0.979
**Acetoacetate**	mmol/l	355	0.02	0.03	0.04	345	0.02	0.03	0.04	393	0.01	0.02	0.02	316	0.01	0.02	0.02	316	0.02	0.02	0.03	393	0.02	0.02	0.03	1.031
**bOHbutyrate**	mmol/l	355	0.18	0.21	0.25	345	0.18	0.21	0.24	393	0.15	0.17	0.19	315	0.15	0.16	0.18	315	0.14	0.16	0.18	392	0.13	0.15	0.17	0.970
**Creatinine**	mmol/l	357	0.03	0.04	0.04	341	0.03	0.04	0.05	392	0.04	0.04	0.05	316	0.04	0.04	0.05	316	0.06	0.06	0.07	390	0.06	0.06	0.07	0.996
**Albumin**	signal area	358	0.07	0.08	0.08	347	0.07	0.08	0.08	393	0.08	0.08	0.08	316	0.08	0.08	0.08	316	0.08	0.09	0.09	393	0.08	0.09	0.09	1.001
**Glycoprotein acetyls**	mmol/l	358	0.65	0.73	0.82	347	0.65	0.73	0.83	393	1.35	1.49	1.67	316	1.30	1.43	1.56	316	1.27	1.44	1.61	393	1.17	1.32	1.48	0.975
**VLDL size**	nm	358	33.9	34.3	34.8	347	34.0	34.4	34.9	393	35.1	35.6	36.2	315	35.0	35.6	36.2	316	36.0	36.8	37.6	393	35.9	36.6	37.3	0.997
**LDL size**	nm	358	22.5	22.9	23.1	347	22.4	22.8	23.1	393	23.6	23.6	23.6	315	23.6	23.6	23.7	316	23.5	23.5	23.6	393	23.5	23.6	23.6	1.001
**HDL size**	nm	358	9.9	10.1	10.2	347	9.8	10.0	10.2	393	10.3	10.4	10.5	315	10.2	10.4	10.5	316	9.7	9.8	9.9	393	9.7	9.8	10.0	1.000
**XXL VLDL P**	nmol/l	358	0.00	0.00	0.01	347	0.00	0.00	0.01	393	0.08	0.11	0.16	315	0.05	0.07	0.11	316	0.10	0.19	0.30	393	0.07	0.11	0.17	0.596
**XL VLDL P**	nmol/l	358	0.00	0.00	0.07	347	0.00	0.02	0.08	393	0.26	0.48	0.81	315	0.13	0.31	0.54	316	0.25	0.72	1.27	393	0.20	0.42	0.75	0.673
**L VLDL P**	nmol/l	358	0.21	0.41	0.70	347	0.25	0.46	0.82	393	2.11	3.46	5.48	315	1.43	2.43	4.10	316	2.77	4.98	7.75	393	1.97	3.31	5.14	0.751
**M VLDL P**	nmol/l	358	1.66	2.47	3.74	347	1.57	2.41	3.91	393	11.00	15.20	20.90	315	8.19	11.50	16.30	316	14.00	20.00	26.30	393	10.60	14.70	19.70	0.793
**S VLDL P**	nmol/l	358	6.1	7.7	10.6	347	6.0	7.7	11.0	393	27.0	34.6	43.8	315	20.8	26.7	33.4	316	27.6	34.3	42.5	393	20.8	26.3	33.2	0.817
**XS VLDL P**	nmol/l	358	15.4	17.8	21.3	347	15.4	17.9	21.4	393	48.4	57.5	68.6	315	36.6	42.8	51.1	316	39.2	48.0	58.2	393	29.4	36.0	43.2	0.781
**IDL P**	nmol/l	358	25.4	32.3	40.5	347	23.5	29.6	37.7	393	130.0	154.0	179.0	315	98.8	113.0	132.0	316	104.0	129.0	155.0	393	79.8	95.3	113.0	0.730
**L LDL P**	nmol/l	358	21.4	32.0	47.5	347	0.00	26.6	41.4	393	207.0	246.0	290.0	315	152.0	179.0	206.0	316	178.0	220.0	271.0	393	129.0	156.0	189.0	0.694
**M LDL P**	nmol/l	358	11.2	20.2	33.2	347	0.00	15.9	28.1	393	164.0	197.0	233.0	315	117.0	139.0	163.0	316	145.0	182.0	225.0	393	103.0	126.0	154.0	0.679
**S LDL P**	nmol/l	358	56.1	64.2	75.2	347	51.9	61.5	72.0	393	193.0	229.0	266.0	315	139.0	165.0	190.0	316	164.0	205.0	248.0	393	122.0	147.0	177.0	0.707
**XL HDL P**	µmol/l	358	0.27	0.38	0.47	347	0.25	0.35	0.47	393	0.75	0.86	1.00	315	0.66	0.79	0.92	316	0.29	0.37	0.47	393	0.30	0.36	0.44	0.940
**L HDL P**	µmol/l	358	0.49	0.69	0.88	347	0.37	0.66	0.84	393	1.69	1.98	2.35	315	1.70	1.98	2.31	316	0.56	0.70	0.90	393	0.61	0.78	0.99	1.090
**M HDL P**	µmol/l	358	0.84	0.98	1.10	347	0.81	0.93	1.05	393	1.81	2.07	2.31	315	1.86	2.08	2.31	316	1.33	1.52	1.71	393	1.37	1.55	1.73	1.028
**S HDL P**	µmol/l	358	2.66	2.86	3.08	347	2.58	2.77	3.00	393	4.31	4.63	4.95	315	4.34	4.63	4.96	316	4.12	4.39	4.63	393	4.21	4.42	4.65	1.025

Data are presented as median (25th-75th percentile). C, cholesterol; VLDL, very low-density lipoprotein; LDL, low-density lipoprotein; HDL, high-density lipoprotein; TG, triglycerides; PG, phosphoglycerides; Apo, apolipoprotein; FA, fatty acid; PUFA, polyunsaturated fatty acid; MUFA, monounsaturated fatty acid; SFA, saturated fatty acid; LA, linoleic acid; DHA, docosahexaenoic acid; bOHbutyrate, β-hydroxybutyrate; P, particle concentration; IDL, intermediate-density lipoprotein; *Metabolites of fathers with hypercholesterolemia (using lipid-lowering treatment) were divided by the metabolite-specific effect of statin treatment relative to placebo found in a study including more than 5000 subjects (Sliz, Circulation 2018).
