# Supplementary material for: Novel associations between parental and newborn cord blood metabolic profiles in the Norwegian Mother, Father and Child Cohort Study
Source: BMC Med. 2021 Apr 14;19:91. doi: 10.1186/s12916-021-01959-w (PMC8045233; doi:10.1186/s12916-021-01959-w)

Directed acyclic graph of the relation between parental and newborn metabolites.

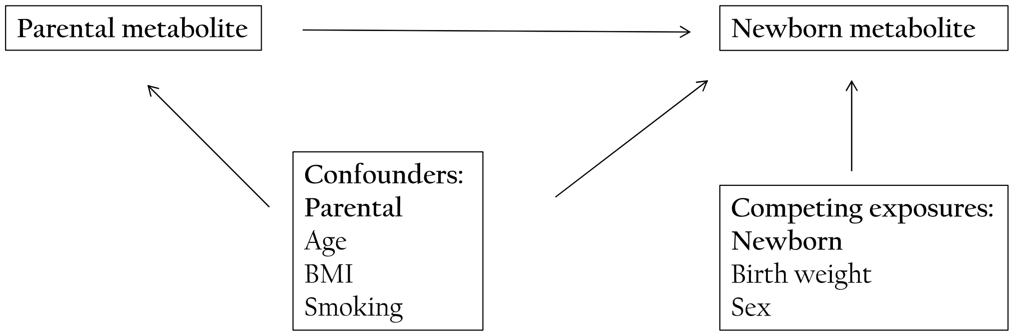

Directed acyclic graph of the relation between newborn sex and metabolites.

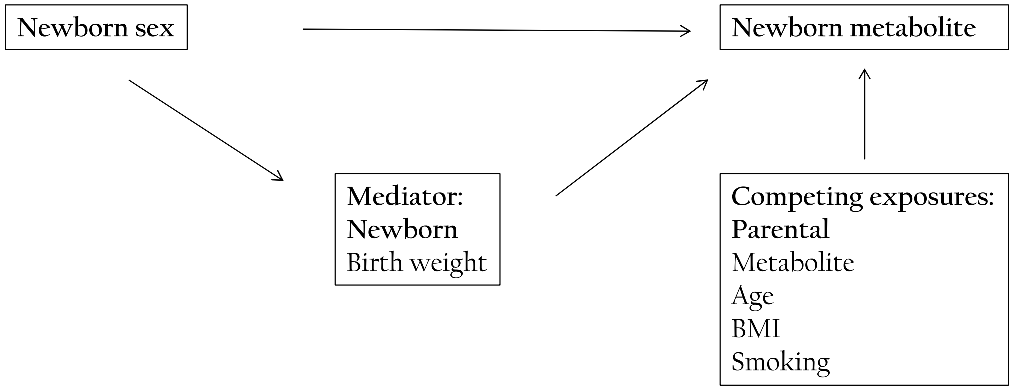

Directed acyclic graph of the relation between newborn birth weight and metabolites.

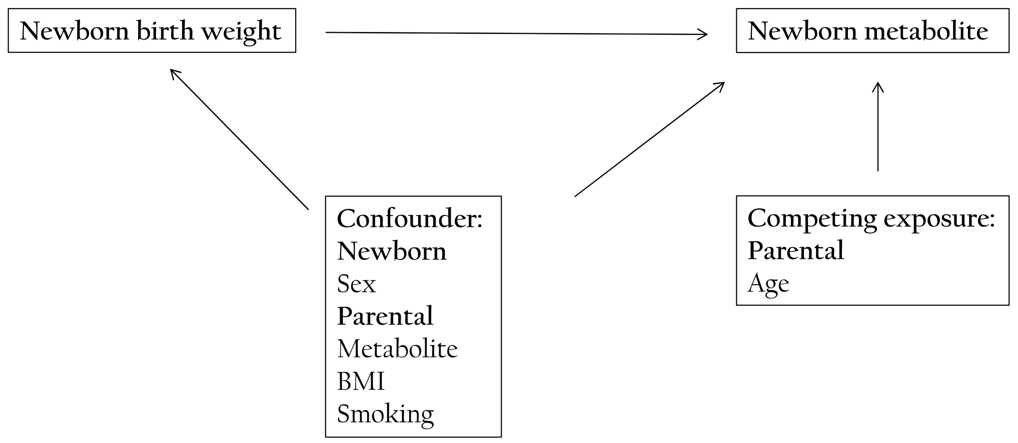

Directed acyclic graph of the relation between parental BMI and newborn metabolites.

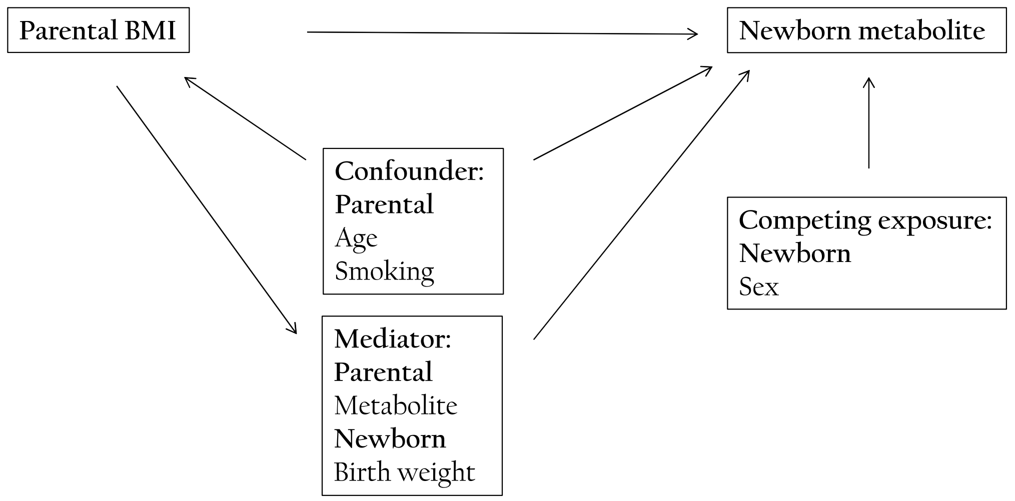

Directed acyclic graph of the relation between parental age and newborn metabolites.

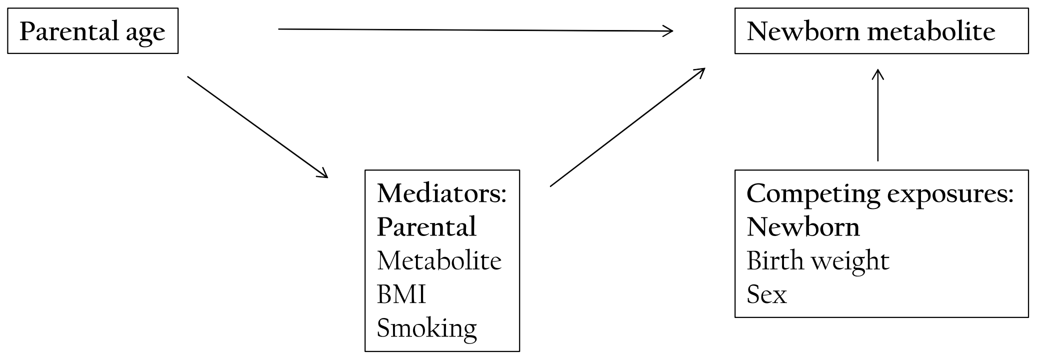

Directed acyclic graph of the relation between parental smoking and newborn metabolites.

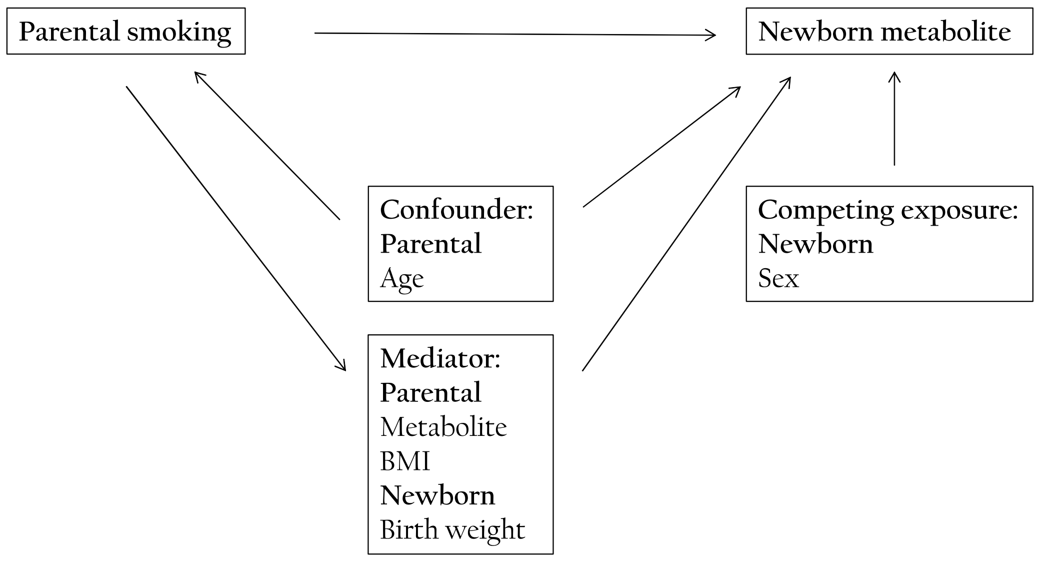

Supplement: Supplementary file 2 — Additional file 2. Directed acyclic graphs of the relation between parental and newborn exposures and newborn metabolites. [file 12916_2021_1959_MOESM2_ESM.pdf]
