# Supplementary material for: Novel associations between parental and newborn cord blood metabolic profiles in the Norwegian Mother, Father and Child Cohort Study
Source: BMC Med. 2021 Apr 14;19:91. doi: 10.1186/s12916-021-01959-w (PMC8045233; doi:10.1186/s12916-021-01959-w)
Supplement: Supplementary file 3 — Additional file 3. Characteristics of subjects who were included and not included. [file 12916_2021_1959_MOESM3_ESM.pdf]

Additional File 3. Characteristics of subjects who were included and not included.

	Mothers		Fathers	
	**Included**	**Not included**	**Included**	**Not included**
**n***	679	71040	679	71040
**Age (years), mean (SD)**	31.7 (4.5)	30.2 (4.6)	34.7 (6.1)	32.8 (5.4)
**BMI** (kg/m^2), mean (SD)**	25.2 (5.0)	24.1 (4.3)	27.0 (3.9)	25.9 (3.3)
**Gestational weight gain (kg), mean (SD)**	14.4 (6.3)	14.8 (6.0)	..	..
**Gestational age (days), median (IQR)**	281 (274-287)	281 (274-287)	..	..
**Education (years), n (%)**				
<12	46 (7.0)	5162 (7.4)	58 (8.9)	7038 (10.6)
12	167 (25.3)	19016 (27.3)	231 (35.5)	24984 (37.5)
13-16	274 (41.6)	28543 (41.3)	200 (30.7)	18526 (27.8)
≥17	172 (26.1)	16830 (24.2)	162 (24.9)	16004 (24.0)
**Smoking during pregnancy, n (%)**	50 (7.4)	5666 (8.0)	108 (16.0)	14044 (20.1)
**CVD, n (%)**	6 (0.9)	240 (0.3)	61 (9.0)	327 (0.5)
**LLT, n (%)**	48 (7.1)	0	303 (44.6)	0
**Total cholesterol (mmol/l), mean (SD)**	5.5 (1.3)	..	3.8 (0.9)	..
**LDL cholesterol (mmol/l), mean (SD)**	2.0 (0.7)	..	1.5 (0.5)	..
**HDL cholesterol (mmol/l), mean (SD)**	1.8 (0.3)	..	1.1 (0.2)	..

Parents who were not included are parents eligible for analysis except those with self-reported hypercholesterolemia or LLT use; SD, standard deviation; IQR, interquartile range; CVD, cardiovascular disease; LLT, self-reported lipid-lowering treatment; LDL, low-density lipoprotein; HDL, high-density lipoprotein. *Includes first participation only (31 mothers participated twice in our sample); **BMI is pre-pregnancy for mothers and peri-pregnancy for fathers (at completion of questionnaire 1).
