# Supplementary material for: Novel associations between parental and newborn cord blood metabolic profiles in the Norwegian Mother, Father and Child Cohort Study
Source: BMC Med. 2021 Apr 14;19:91. doi: 10.1186/s12916-021-01959-w (PMC8045233; doi:10.1186/s12916-021-01959-w)
Supplement: Supplementary file 4 — Additional file 4. Results from mixed model analyses for the association between parental and newborn exposures and newborn metabolites. [file 12916_2021_1959_MOESM4_ESM.pdf]

Additional File 4. Results from mixed model analyses for the association between parental and newborn exposures and newborn metabolites.

	Mothers with hypercholesterolemia										Mothers without hypercholesterolemia									
	Univariable					Multivariable					Univariable					Multivariable				
	Estimate	CI lower	CI higher	p-value	FDR-q	Estimate	CI lower	CI higher	p-value	FDR-q	Estimate	CI lower	CI higher	p-value	FDR-q	Estimate	CI lower	CI higher	p-value	FDR-q
**Total C, mmol/l (log)**																				
Maternal metabolite (log)	0.03	-0.13	0.19	0.733	0.823	0.02	-0.14	0.18	0.791	0.892	-0.08	-0.26	0.10	0.377	0.635	-0.10	-0.28	0.08	0.288	0.627
Paternal metabolite (log)	0.08	-0.05	0.21	0.209	0.830	0.12	0.00	0.25	0.055	0.743	0.10	-0.03	0.23	0.141	0.376	0.11	-0.03	0.24	0.118	0.362
Newborn sex, females vs males	0.10	0.04	0.16	0.001	0.006	0.13	0.07	0.19	0.000	0.001	0.06	-0.01	0.13	0.105	0.493	0.09	0.02	0.16	0.016	0.210
Newborn birth weight, kg	0.07	0.02	0.13	0.013	0.068	0.09	0.03	0.15	0.002	0.015	0.06	0.00	0.13	0.047	0.125	0.07	0.00	0.14	0.046	0.140
Maternal BMI, kg/m^2	0.00	-0.01	0.01	0.823	0.908	0.00	-0.01	0.01	0.675	0.882	0.00	-0.01	0.01	0.994	0.994	0.00	-0.01	0.01	0.690	0.940
Paternal BMI, kg/m^2	0.00	-0.01	0.00	0.309	0.822	-0.01	-0.02	0.00	0.130	0.936	0.00	-0.01	0.01	0.787	0.945	0.00	-0.01	0.01	0.959	0.987
Maternal age, years	0.00	-0.01	0.00	0.174	0.483	-0.01	-0.01	0.00	0.305	0.666	-0.01	-0.01	0.00	0.106	0.378	0.00	-0.01	0.01	0.428	0.995
Paternal age, years	0.00	-0.01	0.00	0.119	0.487	0.00	-0.01	0.01	0.530	0.902	-0.01	-0.01	0.00	0.008	0.063	-0.01	-0.01	0.00	0.045	0.194
Maternal smoking, yes vs no	-0.03	-0.15	0.09	0.634	0.780	-0.02	-0.15	0.10	0.716	0.884	-0.06	-0.19	0.08	0.402	0.805	0.07	-0.08	0.21	0.388	0.653
Paternal smoking, yes vs no	-0.02	-0.11	0.06	0.576	0.877	-0.01	-0.09	0.08	0.877	0.952	-0.06	-0.15	0.04	0.255	0.630	-0.05	-0.16	0.05	0.335	0.651
**n**						363										282				
**VLDL C, mmol/l (log)**																				
Maternal metabolite (log)	0.11	-0.01	0.24	0.074	0.175	0.08	-0.05	0.21	0.239	0.479	0.00	-0.14	0.13	0.949	0.964	-0.08	-0.23	0.07	0.304	0.627
Paternal metabolite (log)	0.01	-0.10	0.12	0.843	0.989	0.01	-0.10	0.13	0.825	0.955	0.11	-0.02	0.24	0.093	0.331	0.13	-0.01	0.26	0.071	0.284
Newborn sex, females vs males	-0.01	-0.10	0.07	0.730	0.798	-0.01	-0.09	0.07	0.818	0.872	0.07	-0.03	0.17	0.177	0.493	0.06	-0.04	0.16	0.262	0.394
Newborn birth weight, kg	-0.03	-0.11	0.04	0.388	0.556	-0.05	-0.12	0.03	0.232	0.354	-0.08	-0.17	0.01	0.095	0.202	-0.06	-0.16	0.04	0.238	0.381
Maternal BMI, kg/m^2	0.01	0.00	0.02	0.062	0.266	0.01	0.00	0.02	0.119	0.485	0.01	-0.01	0.02	0.340	0.811	0.01	0.00	0.02	0.182	0.728
Paternal BMI, kg/m^2	0.00	-0.01	0.02	0.567	0.892	0.00	-0.01	0.01	0.997	0.997	0.00	-0.01	0.02	0.676	0.945	0.00	-0.02	0.01	0.569	0.867
Maternal age, years	-0.01	-0.02	0.00	0.010	0.153	-0.01	-0.02	0.00	0.080	0.468	-0.01	-0.02	0.00	0.113	0.381	0.00	-0.02	0.01	0.525	0.995
Paternal age, years	-0.01	-0.02	0.00	0.105	0.487	0.00	-0.01	0.01	0.701	0.902	-0.01	-0.02	0.00	0.028	0.095	-0.01	-0.02	0.00	0.029	0.184
Maternal smoking, yes vs no	0.16	0.00	0.32	0.050	0.234	0.15	-0.02	0.32	0.082	0.362	0.15	-0.04	0.33	0.128	0.432	0.18	-0.04	0.39	0.112	0.431
Paternal smoking, yes vs no	0.05	-0.06	0.16	0.351	0.764	-0.02	-0.14	0.10	0.788	0.952	0.03	-0.11	0.16	0.711	0.855	0.02	-0.13	0.17	0.773	0.899
**n**						363										282				
**LDL C, mmol/l (log)**																				
Maternal metabolite (log)	-0.04	-0.32	0.25	0.798	0.838	-0.04	-0.32	0.24	0.783	0.892	-0.09	-0.40	0.23	0.584	0.787	-0.07	-0.39	0.24	0.650	0.801
Paternal metabolite (log)	0.12	-0.08	0.33	0.239	0.830	0.17	-0.04	0.37	0.111	0.791	0.11	-0.07	0.30	0.235	0.470	0.10	-0.10	0.29	0.332	0.574
Newborn sex, females vs males	0.31	0.15	0.48	0.000	0.006	0.38	0.22	0.55	0.000	0.001	0.20	0.01	0.39	0.036	0.493	0.28	0.09	0.47	0.004	0.210
Newborn birth weight, kg	0.24	0.08	0.39	0.003	0.043	0.28	0.12	0.44	0.000	0.008	0.21	0.04	0.39	0.014	0.054	0.23	0.04	0.41	0.018	0.069
Maternal BMI, kg/m^2	0.01	-0.01	0.02	0.527	0.697	0.01	-0.01	0.02	0.493	0.800	-0.01	-0.03	0.01	0.433	0.924	-0.01	-0.03	0.01	0.403	0.792
Paternal BMI, kg/m^2	-0.01	-0.04	0.01	0.364	0.822	-0.02	-0.04	0.01	0.149	0.936	-0.01	-0.04	0.01	0.354	0.945	-0.01	-0.03	0.02	0.537	0.867
Maternal age, years	-0.01	-0.03	0.01	0.289	0.543	-0.01	-0.03	0.02	0.560	0.716	-0.02	-0.04	0.00	0.073	0.343	-0.01	-0.04	0.01	0.313	0.995
Paternal age, years	-0.01	-0.03	0.00	0.134	0.487	-0.01	-0.03	0.01	0.452	0.902	-0.02	-0.03	0.00	0.026	0.095	-0.01	-0.03	0.00	0.124	0.331
Maternal smoking, yes vs no	-0.02	-0.35	0.30	0.881	0.924	-0.02	-0.36	0.32	0.915	0.915	-0.04	-0.39	0.31	0.826	0.944	0.22	-0.18	0.62	0.279	0.533
Paternal smoking, yes vs no	-0.04	-0.27	0.19	0.736	0.932	0.01	-0.23	0.25	0.914	0.952	-0.01	-0.27	0.25	0.924	0.933	0.02	-0.27	0.30	0.914	0.965
**n**						363										282				
**HDL C, mmol/l**																				
Maternal metabolite	0.07	0.02	0.13	0.008	0.035	0.08	0.02	0.14	0.006	0.062	0.07	0.00	0.13	0.048	0.163	0.06	0.00	0.13	0.067	0.330
Paternal metabolite	0.06	-0.03	0.14	0.209	0.830	0.06	-0.03	0.15	0.169	0.803	0.03	-0.06	0.13	0.500	0.762	0.05	-0.05	0.15	0.310	0.568
Newborn sex, females vs males	0.07	0.03	0.10	0.000	0.006	0.07	0.04	0.11	0.000	0.001	0.02	-0.02	0.06	0.445	0.663	0.03	-0.01	0.07	0.150	0.299
Newborn birth weight, kg	0.04	0.00	0.07	0.029	0.101	0.06	0.03	0.10	0.001	0.008	0.05	0.02	0.09	0.005	0.030	0.06	0.02	0.10	0.005	0.028
Maternal BMI, kg/m^2	0.00	-0.01	0.00	0.358	0.587	0.00	0.00	0.00	0.817	0.932	0.00	0.00	0.00	0.984	0.994	0.00	-0.01	0.00	0.807	0.970
Paternal BMI, kg/m^2	0.00	-0.01	0.00	0.233	0.822	0.00	-0.01	0.00	0.464	0.936	0.00	-0.01	0.00	0.693	0.945	0.00	0.00	0.01	0.749	0.867
Maternal age, years	0.00	0.00	0.00	0.944	0.974	0.00	-0.01	0.01	0.975	0.979	0.00	-0.01	0.00	0.442	0.566	0.00	-0.01	0.00	0.387	0.995
Paternal age, years	0.00	-0.01	0.00	0.401	0.730	0.00	-0.01	0.00	0.611	0.902	0.00	-0.01	0.00	0.110	0.238	0.00	0.00	0.00	0.595	0.778
Maternal smoking, yes vs no	-0.05	-0.12	0.02	0.171	0.366	-0.04	-0.11	0.04	0.348	0.664	-0.05	-0.13	0.03	0.196	0.503	0.02	-0.07	0.10	0.699	0.798

Paternal smoking, yes vs no	-0.03	-0.08	0.02	0.280	0.749	0.00	-0.05	0.06	0.939	0.952	-0.07	-0.12	-0.01	0.025	0.392	-0.07	-0.13	-0.01	0.033	0.581
**n**						363										282				
**HDL2 C, mmol/l**																				
Maternal metabolite	0.07	0.02	0.12	0.003	0.024	0.08	0.03	0.13	0.003	0.040	0.07	0.01	0.13	0.018	0.081	0.07	0.01	0.13	0.030	0.194
Paternal metabolite	0.05	-0.03	0.12	0.240	0.830	0.05	-0.03	0.13	0.223	0.803	0.06	-0.03	0.15	0.218	0.466	0.07	-0.02	0.16	0.149	0.396
Newborn sex, females vs males	0.05	0.02	0.08	0.001	0.006	0.06	0.03	0.09	0.000	0.002	0.01	-0.02	0.04	0.547	0.729	0.02	-0.01	0.05	0.209	0.361
Newborn birth weight, kg	0.03	0.00	0.06	0.049	0.149	0.05	0.02	0.08	0.001	0.012	0.04	0.01	0.07	0.008	0.038	0.05	0.01	0.08	0.006	0.034
Maternal BMI, kg/m^2	0.00	0.00	0.00	0.328	0.578	0.00	0.00	0.00	0.845	0.932	0.00	0.00	0.00	0.933	0.994	0.00	0.00	0.00	0.898	0.976
Paternal BMI, kg/m^2	0.00	-0.01	0.00	0.246	0.822	0.00	-0.01	0.00	0.522	0.936	0.00	0.00	0.00	0.786	0.945	0.00	0.00	0.01	0.632	0.867
Maternal age, years	0.00	0.00	0.00	0.935	0.974	0.00	0.00	0.01	0.849	0.905	0.00	-0.01	0.00	0.478	0.588	0.00	-0.01	0.00	0.405	0.995
Paternal age, years	0.00	0.00	0.00	0.419	0.730	0.00	-0.01	0.00	0.527	0.902	0.00	0.00	0.00	0.112	0.238	0.00	0.00	0.00	0.595	0.778
Maternal smoking, yes vs no	-0.04	-0.10	0.02	0.161	0.356	-0.03	-0.09	0.03	0.343	0.664	-0.04	-0.11	0.02	0.169	0.471	0.01	-0.06	0.08	0.723	0.812
Paternal smoking, yes vs no	-0.02	-0.06	0.02	0.288	0.749	0.00	-0.04	0.05	0.895	0.952	-0.05	-0.10	-0.01	0.025	0.392	-0.05	-0.10	0.00	0.038	0.581
**n**						363										282				
**HDL3 C, mmol/l**																				
Maternal metabolite	0.08	-0.03	0.19	0.153	0.305	0.08	-0.03	0.20	0.159	0.395	-0.05	-0.17	0.08	0.470	0.733	-0.04	-0.17	0.09	0.544	0.792
Paternal metabolite	0.07	-0.09	0.22	0.410	0.830	0.11	-0.05	0.26	0.194	0.803	-0.12	-0.26	0.02	0.089	0.331	-0.07	-0.22	0.07	0.326	0.574
Newborn sex, females vs males	0.01	0.01	0.02	0.000	0.006	0.02	0.01	0.02	0.000	0.001	0.01	0.00	0.01	0.169	0.493	0.01	0.00	0.02	0.057	0.210
Newborn birth weight, kg	0.01	0.00	0.02	0.006	0.065	0.01	0.01	0.02	0.000	0.008	0.01	0.00	0.02	0.003	0.021	0.01	0.00	0.02	0.004	0.023
Maternal BMI, kg/m^2	0.00	0.00	0.00	0.579	0.704	0.00	0.00	0.00	0.753	0.932	0.00	0.00	0.00	0.755	0.932	0.00	0.00	0.00	0.487	0.799
Paternal BMI, kg/m^2	0.00	0.00	0.00	0.221	0.822	0.00	0.00	0.00	0.144	0.936	0.00	0.00	0.00	0.370	0.945	0.00	0.00	0.00	0.709	0.867
Maternal age, years	0.00	0.00	0.00	0.529	0.702	0.00	0.00	0.00	0.417	0.666	0.00	0.00	0.00	0.337	0.502	0.00	0.00	0.00	0.385	0.995
Paternal age, years	0.00	0.00	0.00	0.433	0.730	0.00	0.00	0.00	0.945	0.960	0.00	0.00	0.00	0.142	0.275	0.00	0.00	0.00	0.547	0.778
Maternal smoking, yes vs no	-0.01	-0.02	0.01	0.285	0.506	-0.01	-0.02	0.01	0.410	0.664	-0.01	-0.02	0.01	0.355	0.734	0.00	-0.01	0.02	0.593	0.754
Paternal smoking, yes vs no	-0.01	-0.02	0.01	0.319	0.749	0.00	-0.01	0.01	0.795	0.952	-0.01	-0.02	0.00	0.062	0.444	-0.01	-0.02	0.00	0.083	0.581
**n**						363										282				
**Remnant C, mmol/l (log)**																				
Maternal metabolite (log)	0.07	-0.10	0.23	0.428	0.595	0.03	-0.14	0.20	0.739	0.892	-0.06	-0.24	0.12	0.507	0.755	-0.12	-0.31	0.07	0.225	0.627
Paternal metabolite (log)	0.01	-0.12	0.14	0.874	0.989	0.05	-0.09	0.18	0.520	0.955	0.16	0.00	0.31	0.044	0.283	0.16	0.00	0.32	0.045	0.264
Newborn sex, females vs males	0.07	-0.02	0.15	0.147	0.265	0.08	-0.01	0.17	0.071	0.138	0.09	-0.02	0.19	0.107	0.493	0.10	-0.01	0.20	0.082	0.210
Newborn birth weight, kg	0.01	-0.07	0.09	0.798	0.877	0.01	-0.07	0.10	0.784	0.865	-0.01	-0.11	0.08	0.789	0.856	0.01	-0.10	0.11	0.909	0.953
Maternal BMI, kg/m^2	0.01	0.00	0.02	0.158	0.421	0.01	0.00	0.02	0.128	0.485	0.00	-0.01	0.01	0.598	0.932	0.00	-0.01	0.02	0.483	0.799
Paternal BMI, kg/m^2	0.00	-0.01	0.01	0.994	0.994	0.00	-0.02	0.01	0.552	0.936	0.00	-0.01	0.01	0.841	0.946	0.00	-0.02	0.01	0.794	0.892
Maternal age, years	-0.01	-0.02	0.00	0.006	0.123	-0.01	-0.03	0.00	0.057	0.468	-0.01	-0.02	0.00	0.069	0.343	-0.01	-0.02	0.01	0.422	0.995
Paternal age, years	-0.01	-0.02	0.00	0.044	0.487	0.00	-0.02	0.01	0.640	0.902	-0.01	-0.02	0.00	0.008	0.063	-0.01	-0.02	0.00	0.019	0.135
Maternal smoking, yes vs no	0.12	-0.05	0.29	0.178	0.367	0.11	-0.07	0.29	0.223	0.550	0.07	-0.13	0.26	0.498	0.858	0.15	-0.08	0.37	0.198	0.452
Paternal smoking, yes vs no	0.04	-0.08	0.16	0.508	0.855	0.00	-0.13	0.13	0.952	0.952	-0.01	-0.15	0.14	0.933	0.933	0.00	-0.16	0.15	0.951	0.965
**n**						363.00										282.00				
**Esterified-C, mmol/l**																				
Maternal metabolite	0.01	-0.02	0.05	0.515	0.646	0.01	-0.02	0.05	0.502	0.784	-0.01	-0.08	0.06	0.861	0.918	-0.01	-0.08	0.06	0.723	0.801
Paternal metabolite	0.03	-0.02	0.08	0.271	0.830	0.05	-0.01	0.10	0.084	0.743	0.04	-0.01	0.09	0.120	0.349	0.05	0.00	0.10	0.078	0.293
Newborn sex, females vs males	0.11	0.05	0.17	0.001	0.006	0.13	0.07	0.20	0.000	0.001	0.08	-0.01	0.17	0.070	0.493	0.12	0.02	0.21	0.013	0.210
Newborn birth weight, kg	0.08	0.03	0.14	0.005	0.064	0.11	0.05	0.17	0.001	0.008	0.08	0.00	0.16	0.061	0.149	0.09	0.00	0.18	0.056	0.160
Maternal BMI, kg/m^2	0.00	-0.01	0.01	0.975	0.975	0.00	-0.01	0.01	0.961	0.976	0.00	-0.01	0.01	0.597	0.932	0.00	-0.01	0.01	0.723	0.945
Paternal BMI, kg/m^2	0.00	-0.01	0.00	0.352	0.822	-0.01	-0.02	0.00	0.194	0.936	0.00	-0.01	0.01	0.894	0.976	0.00	-0.01	0.01	0.972	0.987
Maternal age, years	0.00	-0.01	0.00	0.248	0.541	0.00	-0.01	0.01	0.360	0.666	-0.01	-0.02	0.00	0.212	0.411	0.00	-0.02	0.01	0.502	0.995
Paternal age, years	0.00	-0.01	0.00	0.133	0.487	0.00	-0.01	0.01	0.531	0.902	-0.01	-0.01	0.00	0.055	0.153	-0.01	-0.01	0.00	0.241	0.550
Maternal smoking, yes vs no	-0.02	-0.14	0.10	0.746	0.875	-0.01	-0.14	0.11	0.835	0.909	-0.06	-0.24	0.11	0.468	0.858	0.05	-0.14	0.24	0.609	0.754
Paternal smoking, yes vs no	-0.02	-0.11	0.07	0.660	0.917	0.00	-0.10	0.09	0.933	0.952	-0.08	-0.21	0.05	0.210	0.586	-0.08	-0.22	0.06	0.257	0.633
**n**						360										283				
**Free C, mmol/l (log)**																				
Maternal metabolite (log)	0.02	-0.11	0.15	0.718	0.822	0.00	-0.13	0.13	0.995	0.995	-0.03	-0.19	0.13	0.731	0.808	-0.02	-0.18	0.13	0.765	0.801
Paternal metabolite (log)	0.06	-0.05	0.16	0.277	0.830	0.10	-0.01	0.21	0.071	0.743	0.14	0.02	0.25	0.022	0.219	0.14	0.02	0.26	0.018	0.211

Newborn sex, females vs males	0.08	0.03	0.12	0.002	0.012	0.08	0.03	0.13	0.001	0.007	0.03	-0.03	0.09	0.271	0.509	0.05	-0.01	0.11	0.082	0.210
Newborn birth weight, kg	-0.01	-0.05	0.04	0.809	0.877	0.00	-0.04	0.05	0.860	0.918	0.02	-0.04	0.07	0.525	0.659	0.01	-0.05	0.07	0.678	0.775
Maternal BMI, kg/m^2	0.00	-0.01	0.00	0.927	0.951	0.00	0.00	0.01	0.713	0.913	0.00	-0.01	0.01	0.782	0.935	0.00	-0.01	0.01	0.969	0.976
Paternal BMI, kg/m^2	0.00	-0.01	0.01	0.834	0.941	0.00	-0.01	0.01	0.596	0.936	0.00	-0.01	0.01	0.719	0.945	0.00	-0.01	0.01	0.759	0.867
Maternal age, years	-0.01	-0.01	0.00	0.035	0.240	-0.01	-0.01	0.00	0.092	0.468	0.00	-0.01	0.00	0.403	0.537	0.00	-0.01	0.01	0.727	0.995
Paternal age, years	0.00	-0.01	0.00	0.069	0.487	0.00	-0.01	0.01	0.632	0.902	-0.01	-0.01	0.00	0.002	0.063	-0.01	-0.01	0.00	0.007	0.108
Maternal smoking, yes vs no	-0.04	-0.14	0.05	0.361	0.551	-0.04	-0.14	0.06	0.455	0.678	-0.07	-0.18	0.04	0.230	0.565	0.03	-0.09	0.15	0.650	0.770
Paternal smoking, yes vs no	-0.02	-0.08	0.05	0.601	0.894	-0.01	-0.08	0.06	0.691	0.952	-0.07	-0.15	0.02	0.117	0.483	-0.06	-0.15	0.03	0.189	0.633
**n**						360										283				
**Total TG, mmol/l (log)**																				
Maternal metabolite (log)	0.15	0.04	0.26	0.010	0.041	0.11	-0.01	0.24	0.078	0.278	0.08	-0.04	0.20	0.210	0.433	-0.02	-0.15	0.12	0.788	0.801
Paternal metabolite (log)	0.04	-0.07	0.14	0.473	0.834	0.01	-0.10	0.13	0.859	0.955	0.06	-0.05	0.17	0.263	0.510	0.06	-0.05	0.18	0.299	0.568
Newborn sex, females vs males	-0.06	-0.14	0.02	0.125	0.250	-0.06	-0.14	0.02	0.129	0.222	0.03	-0.06	0.12	0.480	0.683	0.01	-0.08	0.10	0.810	0.929
Newborn birth weight, kg	-0.01	-0.08	0.07	0.883	0.927	-0.03	-0.11	0.04	0.383	0.511	-0.06	-0.14	0.02	0.144	0.251	-0.05	-0.13	0.04	0.293	0.447
Maternal BMI, kg/m^2	0.01	0.00	0.02	0.008	0.053	0.01	0.00	0.02	0.082	0.485	0.01	0.00	0.02	0.110	0.641	0.01	0.00	0.02	0.102	0.634
Paternal BMI, kg/m^2	0.01	-0.01	0.02	0.346	0.822	0.00	-0.01	0.01	0.812	0.974	0.00	-0.01	0.02	0.437	0.945	0.00	-0.02	0.01	0.606	0.867
Maternal age, years	-0.01	-0.01	0.00	0.254	0.541	0.00	-0.02	0.01	0.475	0.685	-0.01	-0.02	0.00	0.187	0.411	0.00	-0.01	0.01	0.645	0.995
Paternal age, years	0.00	-0.01	0.00	0.451	0.740	0.00	-0.01	0.01	0.600	0.902	-0.01	-0.01	0.00	0.054	0.153	-0.01	-0.02	0.00	0.035	0.185
Maternal smoking, yes vs no	0.13	-0.02	0.28	0.093	0.297	0.13	-0.03	0.29	0.113	0.362	0.19	0.03	0.35	0.021	0.223	0.23	0.04	0.42	0.017	0.361
Paternal smoking, yes vs no	0.02	-0.09	0.12	0.778	0.932	-0.06	-0.17	0.06	0.347	0.952	0.03	-0.09	0.16	0.584	0.767	0.04	-0.09	0.17	0.560	0.874
**n**						363										282				
**VLDL TG, mmol/l (log)**																				
Maternal metabolite (log)	0.13	0.04	0.22	0.004	0.024	0.11	0.01	0.21	0.034	0.129	0.12	0.02	0.22	0.017	0.081	0.05	-0.06	0.16	0.362	0.656
Paternal metabolite (log)	0.03	-0.07	0.13	0.568	0.909	-0.01	-0.12	0.10	0.890	0.955	0.08	-0.02	0.18	0.108	0.345	0.08	-0.03	0.19	0.140	0.390
Newborn sex, females vs males	-0.09	-0.17	0.00	0.058	0.155	-0.09	-0.18	0.00	0.045	0.114	0.02	-0.08	0.12	0.669	0.766	-0.01	-0.11	0.09	0.828	0.929
Newborn birth weight, kg	-0.04	-0.12	0.05	0.391	0.556	-0.07	-0.16	0.01	0.082	0.168	-0.10	-0.18	-0.01	0.036	0.109	-0.09	-0.19	0.00	0.059	0.160
Maternal BMI, kg/m^2	0.01	0.00	0.02	0.005	0.053	0.01	0.00	0.02	0.064	0.485	0.01	0.00	0.02	0.091	0.641	0.01	0.00	0.02	0.148	0.714
Paternal BMI, kg/m^2	0.01	-0.01	0.02	0.304	0.822	0.00	-0.01	0.02	0.687	0.951	0.01	-0.01	0.02	0.402	0.945	-0.01	-0.02	0.01	0.464	0.867
Maternal age, years	-0.01	-0.02	0.00	0.241	0.541	-0.01	-0.02	0.01	0.412	0.666	-0.01	-0.02	0.00	0.143	0.411	0.00	-0.02	0.01	0.541	0.995
Paternal age, years	0.00	-0.01	0.01	0.551	0.756	0.00	-0.01	0.01	0.714	0.902	-0.01	-0.01	0.00	0.142	0.275	-0.01	-0.02	0.00	0.110	0.319
Maternal smoking, yes vs no	0.16	-0.01	0.33	0.066	0.234	0.16	-0.02	0.34	0.085	0.362	0.25	0.07	0.43	0.007	0.137	0.24	0.03	0.45	0.024	0.386
Paternal smoking, yes vs no	0.02	-0.10	0.14	0.762	0.932	-0.08	-0.21	0.05	0.234	0.952	0.06	-0.07	0.20	0.366	0.659	0.06	-0.09	0.20	0.460	0.775
**n**						363										282				
**LDL TG, mmol/l (log)**																				
Maternal metabolite (log)	0.17	0.00	0.35	0.052	0.130	0.12	-0.07	0.30	0.229	0.479	-0.03	-0.21	0.14	0.705	0.808	-0.13	-0.31	0.05	0.161	0.491
Paternal metabolite (log)	0.07	-0.06	0.20	0.309	0.830	0.06	-0.09	0.20	0.459	0.955	0.06	-0.09	0.20	0.438	0.719	0.06	-0.10	0.21	0.478	0.728
Newborn sex, females vs males	-0.03	-0.12	0.06	0.534	0.683	-0.02	-0.11	0.08	0.702	0.781	0.04	-0.06	0.14	0.389	0.622	0.04	-0.06	0.15	0.397	0.540
Newborn birth weight, kg	0.06	-0.03	0.15	0.167	0.324	0.05	-0.04	0.14	0.318	0.442	0.00	-0.09	0.09	0.959	0.959	0.03	-0.07	0.13	0.584	0.719
Maternal BMI, kg/m^2	0.01	0.00	0.02	0.021	0.125	0.01	0.00	0.02	0.130	0.485	0.01	-0.01	0.02	0.342	0.811	0.01	-0.01	0.02	0.328	0.764
Paternal BMI, kg/m^2	0.00	-0.01	0.02	0.534	0.892	0.00	-0.02	0.01	0.873	0.974	0.00	-0.01	0.01	0.745	0.945	0.00	-0.02	0.01	0.724	0.867
Maternal age, years	-0.01	-0.02	0.01	0.346	0.594	-0.01	-0.02	0.01	0.514	0.685	-0.01	-0.02	0.01	0.359	0.523	0.00	-0.01	0.01	0.993	0.995
Paternal age, years	0.00	-0.01	0.01	0.431	0.730	0.00	-0.02	0.01	0.586	0.902	-0.01	-0.02	0.00	0.010	0.063	-0.01	-0.02	0.00	0.006	0.108
Maternal smoking, yes vs no	0.14	-0.05	0.32	0.144	0.342	0.13	-0.07	0.33	0.196	0.524	0.12	-0.06	0.31	0.194	0.503	0.27	0.05	0.48	0.015	0.361
Paternal smoking, yes vs no	0.02	-0.10	0.15	0.700	0.932	-0.02	-0.16	0.12	0.760	0.952	0.01	-0.13	0.15	0.863	0.921	0.03	-0.13	0.18	0.742	0.899
**n**						363										282				
**HDL TG, mmol/l (log)**																				
Maternal metabolite (log)	0.20	0.00	0.39	0.053	0.130	0.17	-0.05	0.38	0.127	0.339	-0.04	-0.25	0.16	0.676	0.808	-0.15	-0.37	0.07	0.175	0.510
Paternal metabolite (log)	0.06	-0.10	0.21	0.482	0.834	0.05	-0.12	0.21	0.600	0.955	0.00	-0.15	0.16	0.964	0.986	0.00	-0.17	0.17	0.985	0.985
Newborn sex, females vs males	0.02	-0.07	0.11	0.679	0.790	0.02	-0.07	0.12	0.618	0.781	0.06	-0.03	0.16	0.206	0.493	0.06	-0.04	0.16	0.253	0.394
Newborn birth weight, kg	0.02	-0.06	0.10	0.652	0.802	0.01	-0.07	0.10	0.735	0.825	0.01	-0.08	0.10	0.865	0.920	0.03	-0.07	0.13	0.521	0.675
Maternal BMI, kg/m^2	0.01	0.00	0.02	0.167	0.428	0.00	-0.01	0.01	0.426	0.800	0.01	0.00	0.02	0.322	0.811	0.01	-0.01	0.02	0.287	0.764
Paternal BMI, kg/m^2	0.00	-0.01	0.02	0.547	0.892	0.00	-0.01	0.01	0.935	0.974	0.01	-0.01	0.02	0.391	0.945	0.00	-0.01	0.02	0.705	0.867
Maternal age, years	-0.01	-0.02	0.00	0.208	0.534	0.00	-0.02	0.01	0.509	0.685	-0.01	-0.02	0.01	0.330	0.502	0.00	-0.02	0.01	0.659	0.995

Paternal age, years	-0.01	-0.01	0.00	0.266	0.621	0.00	-0.02	0.01	0.528	0.902	-0.01	-0.01	0.00	0.047	0.143	-0.01	-0.02	0.00	0.040	0.185
Maternal smoking, yes vs no	0.05	-0.12	0.22	0.554	0.715	0.06	-0.12	0.24	0.521	0.757	0.09	-0.09	0.28	0.307	0.677	0.20	-0.01	0.41	0.060	0.431
Paternal smoking, yes vs no	0.00	-0.12	0.12	0.962	0.983	-0.03	-0.16	0.10	0.620	0.952	-0.05	-0.18	0.09	0.478	0.696	-0.02	-0.17	0.12	0.765	0.899
**n**						363										282				
**PG, mmol/l (log)**																				
Maternal metabolite (log)	0.08	-0.09	0.25	0.344	0.546	0.05	-0.12	0.23	0.548	0.797	0.03	-0.14	0.21	0.713	0.808	-0.03	-0.21	0.14	0.706	0.801
Paternal metabolite (log)	0.08	-0.03	0.18	0.161	0.830	0.09	-0.03	0.20	0.129	0.803	0.14	0.01	0.27	0.041	0.283	0.17	0.03	0.30	0.016	0.211
Newborn sex, females vs males	0.05	0.00	0.10	0.057	0.155	0.05	0.00	0.11	0.052	0.115	0.05	-0.02	0.11	0.166	0.493	0.06	0.00	0.13	0.071	0.210
Newborn birth weight, kg	-0.03	-0.07	0.02	0.297	0.475	-0.02	-0.07	0.03	0.497	0.600	0.04	-0.02	0.10	0.218	0.359	0.04	-0.02	0.11	0.199	0.355
Maternal BMI, kg/m^2	0.00	0.00	0.01	0.245	0.507	0.00	0.00	0.01	0.331	0.654	0.01	0.00	0.01	0.031	0.574	0.01	0.00	0.02	0.041	0.634
Paternal BMI, kg/m^2	0.00	-0.01	0.01	0.753	0.892	0.00	-0.01	0.01	0.731	0.974	0.00	-0.01	0.01	0.794	0.945	0.00	-0.01	0.01	0.424	0.867
Maternal age, years	0.00	-0.01	0.00	0.424	0.642	-0.01	-0.02	0.00	0.128	0.468	-0.01	-0.01	0.00	0.064	0.343	0.00	-0.01	0.01	0.419	0.995
Paternal age, years	0.00	0.00	0.01	0.789	0.837	0.00	0.00	0.01	0.404	0.902	-0.01	-0.01	0.00	0.007	0.063	-0.01	-0.01	0.00	0.037	0.185
Maternal smoking, yes vs no	0.08	-0.02	0.18	0.137	0.337	0.05	-0.06	0.16	0.357	0.664	-0.01	-0.13	0.12	0.908	0.961	0.09	-0.05	0.23	0.195	0.452
Paternal smoking, yes vs no	0.06	-0.02	0.13	0.124	0.589	0.03	-0.04	0.11	0.395	0.952	-0.05	-0.14	0.05	0.322	0.659	-0.04	-0.14	0.05	0.383	0.682
**n**						358										283				
**TG/PG (log)**																				
Maternal metabolite (log)	0.04	-0.10	0.18	0.609	0.736	0.01	-0.15	0.17	0.948	0.963	0.09	-0.07	0.24	0.286	0.524	0.05	-0.12	0.22	0.572	0.792
Paternal metabolite (log)	-0.01	-0.09	0.08	0.881	0.989	-0.03	-0.13	0.07	0.578	0.955	0.00	-0.10	0.10	0.980	0.986	-0.02	-0.12	0.09	0.771	0.872
Newborn sex, females vs males	-0.04	-0.06	-0.01	0.004	0.021	-0.04	-0.06	-0.01	0.005	0.021	0.00	-0.02	0.03	0.889	0.933	0.00	-0.03	0.02	0.726	0.860
Newborn birth weight, kg	-0.01	-0.03	0.02	0.669	0.807	-0.02	-0.04	0.01	0.219	0.353	-0.04	-0.06	-0.01	0.002	0.019	-0.04	-0.07	-0.02	0.002	0.018
Maternal BMI, kg/m^2	0.00	0.00	0.00	0.111	0.348	0.00	0.00	0.01	0.171	0.497	0.00	0.00	0.00	0.272	0.811	0.00	0.00	0.00	0.305	0.764
Paternal BMI, kg/m^2	0.00	0.00	0.00	0.562	0.892	0.00	0.00	0.01	0.488	0.936	0.00	0.00	0.00	0.710	0.945	0.00	0.00	0.00	0.587	0.867
Maternal age, years	0.00	0.00	0.00	0.461	0.642	0.00	0.00	0.00	0.649	0.784	0.00	0.00	0.01	0.211	0.411	0.00	0.00	0.01	0.272	0.995
Paternal age, years	0.00	0.00	0.00	0.182	0.487	0.00	-0.01	0.00	0.119	0.902	0.00	0.00	0.00	0.336	0.524	0.00	0.00	0.00	0.995	0.996
Maternal smoking, yes vs no	0.02	-0.02	0.07	0.325	0.534	0.02	-0.03	0.07	0.394	0.664	0.06	0.01	0.11	0.015	0.187	0.03	-0.03	0.08	0.346	0.614
Paternal smoking, yes vs no	0.01	-0.02	0.05	0.507	0.855	0.00	-0.04	0.03	0.849	0.952	0.04	0.01	0.08	0.020	0.392	0.04	0.00	0.08	0.041	0.581
**n**						358										283				
**Cholines, mmol/l (log)**																				
Maternal metabolite (log)	0.05	-0.09	0.19	0.495	0.646	0.04	-0.11	0.18	0.625	0.837	0.09	-0.08	0.26	0.295	0.524	0.05	-0.12	0.22	0.578	0.792
Paternal metabolite (log)	0.05	-0.05	0.14	0.339	0.830	0.05	-0.05	0.15	0.309	0.955	0.17	0.04	0.31	0.014	0.219	0.20	0.05	0.34	0.007	0.211
Newborn sex, females vs males	0.03	-0.01	0.07	0.109	0.240	0.03	-0.01	0.07	0.142	0.233	0.03	-0.02	0.08	0.268	0.509	0.04	-0.02	0.10	0.163	0.317
Newborn birth weight, kg	-0.05	-0.08	-0.01	0.010	0.068	-0.04	-0.08	0.00	0.033	0.101	0.02	-0.03	0.07	0.515	0.659	0.02	-0.04	0.07	0.538	0.675
Maternal BMI, kg/m^2	0.00	0.00	0.01	0.574	0.704	0.00	0.00	0.01	0.548	0.800	0.01	0.00	0.01	0.039	0.574	0.01	0.00	0.01	0.033	0.634
Paternal BMI, kg/m^2	0.00	0.00	0.01	0.529	0.892	0.00	-0.01	0.01	0.835	0.974	0.00	-0.01	0.01	0.960	0.979	0.00	-0.01	0.00	0.318	0.867
Maternal age, years	0.00	-0.01	0.00	0.392	0.627	-0.01	-0.01	0.00	0.102	0.468	0.00	-0.01	0.00	0.190	0.411	0.00	-0.01	0.01	0.708	0.995
Paternal age, years	0.00	0.00	0.01	0.567	0.756	0.00	0.00	0.01	0.193	0.902	0.00	-0.01	0.00	0.034	0.110	0.00	-0.01	0.00	0.081	0.252
Maternal smoking, yes vs no	0.04	-0.04	0.11	0.325	0.534	0.01	-0.07	0.09	0.794	0.907	-0.02	-0.12	0.08	0.696	0.858	0.03	-0.08	0.15	0.560	0.754
Paternal smoking, yes vs no	0.05	0.00	0.11	0.046	0.325	0.04	-0.02	0.10	0.172	0.952	-0.04	-0.12	0.04	0.319	0.659	-0.04	-0.12	0.05	0.372	0.680
**n**						358										283				
**Phosphatidylcholines, mmol/l (log)**																				
Maternal metabolite (log)	0.05	-0.10	0.19	0.503	0.646	0.02	-0.13	0.17	0.808	0.892	0.03	-0.13	0.19	0.717	0.808	-0.02	-0.18	0.14	0.771	0.801
Paternal metabolite (log)	0.06	-0.04	0.16	0.271	0.830	0.07	-0.04	0.17	0.226	0.803	0.11	-0.01	0.24	0.075	0.331	0.15	0.02	0.27	0.027	0.223
Newborn sex, females vs males	0.05	0.00	0.09	0.035	0.113	0.05	0.00	0.10	0.035	0.096	0.05	-0.01	0.10	0.124	0.493	0.06	0.00	0.12	0.035	0.210
Newborn birth weight, kg	-0.02	-0.06	0.02	0.369	0.550	-0.01	-0.06	0.03	0.610	0.697	0.05	0.00	0.10	0.063	0.149	0.05	-0.01	0.11	0.098	0.231
Maternal BMI, kg/m^2	0.00	0.00	0.01	0.380	0.594	0.00	0.00	0.01	0.490	0.800	0.01	0.00	0.01	0.091	0.641	0.01	0.00	0.01	0.108	0.634
Paternal BMI, kg/m^2	0.00	-0.01	0.01	0.648	0.892	0.00	-0.01	0.01	0.904	0.974	0.00	-0.01	0.01	0.987	0.987	0.00	-0.01	0.00	0.420	0.867
Maternal age, years	0.00	-0.01	0.00	0.408	0.637	-0.01	-0.01	0.00	0.142	0.468	0.00	-0.01	0.00	0.175	0.411	0.00	-0.01	0.01	0.624	0.995
Paternal age, years	0.00	0.00	0.01	0.839	0.852	0.00	0.00	0.01	0.393	0.902	-0.01	-0.01	0.00	0.019	0.079	0.00	-0.01	0.00	0.083	0.252
Maternal smoking, yes vs no	0.05	-0.04	0.14	0.256	0.471	0.03	-0.06	0.12	0.541	0.770	-0.01	-0.12	0.10	0.817	0.944	0.08	-0.04	0.20	0.207	0.456
Paternal smoking, yes vs no	0.04	-0.02	0.10	0.158	0.632	0.03	-0.04	0.10	0.370	0.952	-0.05	-0.13	0.03	0.256	0.630	-0.05	-0.13	0.04	0.301	0.633
**n**						358										283				
**Sphingomyelins, mmol/l**																				

Maternal metabolite	0.01	-0.06	0.07	0.839	0.866	0.00	-0.06	0.07	0.913	0.943	0.04	-0.06	0.15	0.405	0.649	0.04	-0.07	0.14	0.498	0.777
Paternal metabolite	0.00	-0.07	0.07	0.947	0.993	0.01	-0.07	0.08	0.834	0.955	0.07	-0.01	0.15	0.093	0.331	0.08	0.00	0.17	0.056	0.276
Newborn sex, females vs males	0.02	0.00	0.03	0.012	0.044	0.01	0.00	0.03	0.037	0.098	0.01	-0.01	0.02	0.401	0.626	0.01	-0.01	0.03	0.226	0.381
Newborn birth weight, kg	-0.02	-0.03	-0.01	0.003	0.043	-0.02	-0.03	0.00	0.014	0.048	0.01	-0.01	0.02	0.270	0.421	0.01	-0.01	0.03	0.272	0.425
Maternal BMI, kg/m^2	0.00	0.00	0.00	0.924	0.951	0.00	0.00	0.00	0.600	0.828	0.00	0.00	0.00	0.328	0.811	0.00	0.00	0.00	0.206	0.764
Paternal BMI, kg/m^2	0.00	0.00	0.00	0.879	0.960	0.00	0.00	0.00	0.698	0.951	0.00	0.00	0.00	0.786	0.945	0.00	0.00	0.00	0.720	0.867
Maternal age, years	0.00	0.00	0.00	0.455	0.642	0.00	0.00	0.00	0.152	0.468	0.00	0.00	0.00	0.257	0.433	0.00	0.00	0.00	0.753	0.995
Paternal age, years	0.00	0.00	0.00	0.818	0.844	0.00	0.00	0.00	0.288	0.902	0.00	0.00	0.00	0.028	0.095	0.00	0.00	0.00	0.057	0.196
Maternal smoking, yes vs no	0.01	-0.01	0.04	0.395	0.589	0.00	-0.02	0.03	0.729	0.884	0.00	-0.04	0.03	0.829	0.944	0.01	-0.02	0.05	0.471	0.719
Paternal smoking, yes vs no	0.01	0.00	0.03	0.119	0.589	0.01	-0.01	0.03	0.353	0.952	-0.01	-0.04	0.01	0.301	0.659	-0.01	-0.04	0.02	0.445	0.769
**n**						358										283				
**ApoB, g/l (log)**																				
Maternal metabolite (log)	0.04	-0.05	0.14	0.360	0.546	0.03	-0.07	0.12	0.560	0.797	-0.02	-0.14	0.10	0.732	0.808	-0.06	-0.19	0.07	0.357	0.656
Paternal metabolite (log)	0.02	-0.07	0.11	0.723	0.978	0.04	-0.05	0.13	0.374	0.955	0.13	0.03	0.24	0.014	0.219	0.13	0.02	0.24	0.020	0.211
Newborn sex, females vs males	0.04	0.00	0.08	0.068	0.173	0.05	0.01	0.09	0.025	0.082	0.04	-0.01	0.09	0.110	0.493	0.06	0.00	0.11	0.040	0.210
Newborn birth weight, kg	0.03	-0.01	0.07	0.142	0.294	0.03	-0.01	0.07	0.128	0.235	0.01	-0.04	0.06	0.737	0.828	0.01	-0.04	0.06	0.650	0.771
Maternal BMI, kg/m^2	0.00	0.00	0.01	0.205	0.451	0.00	0.00	0.01	0.204	0.522	0.00	0.00	0.01	0.642	0.932	0.00	0.00	0.01	0.541	0.819
Paternal BMI, kg/m^2	0.00	-0.01	0.00	0.751	0.892	0.00	-0.01	0.00	0.345	0.936	0.00	-0.01	0.01	0.942	0.979	0.00	-0.01	0.01	0.740	0.867
Maternal age, years	0.00	-0.01	0.00	0.069	0.274	0.00	-0.01	0.00	0.224	0.572	-0.01	-0.01	0.00	0.086	0.343	0.00	-0.01	0.00	0.433	0.995
Paternal age, years	0.00	-0.01	0.00	0.099	0.487	0.00	-0.01	0.00	0.495	0.902	0.00	-0.01	0.00	0.011	0.063	0.00	-0.01	0.00	0.040	0.185
Maternal smoking, yes vs no	0.03	-0.05	0.11	0.448	0.652	0.03	-0.05	0.12	0.434	0.664	0.02	-0.08	0.12	0.668	0.858	0.08	-0.03	0.19	0.141	0.431
Paternal smoking, yes vs no	0.00	-0.05	0.06	0.968	0.983	-0.01	-0.07	0.05	0.738	0.952	-0.01	-0.08	0.06	0.737	0.858	-0.02	-0.09	0.06	0.668	0.899
**n**						363										282				
**ApoA1, g/l**																				
Maternal metabolite	0.07	0.02	0.13	0.012	0.046	0.08	0.02	0.14	0.008	0.073	0.04	-0.03	0.11	0.295	0.524	0.03	-0.04	0.10	0.356	0.656
Paternal metabolite	0.06	-0.02	0.14	0.144	0.830	0.08	0.00	0.16	0.063	0.743	0.04	-0.05	0.13	0.401	0.710	0.06	-0.03	0.15	0.186	0.459
Newborn sex, females vs males	0.04	0.02	0.06	0.001	0.007	0.04	0.02	0.07	0.000	0.002	0.01	-0.01	0.04	0.266	0.509	0.02	0.00	0.05	0.079	0.210
Newborn birth weight, kg	0.03	0.00	0.05	0.015	0.075	0.04	0.02	0.06	0.000	0.008	0.03	0.01	0.06	0.006	0.030	0.04	0.01	0.06	0.007	0.036
Maternal BMI, kg/m^2	0.00	0.00	0.00	0.544	0.697	0.00	0.00	0.00	0.898	0.951	0.00	0.00	0.00	0.715	0.932	0.00	0.00	0.00	0.958	0.976
Paternal BMI, kg/m^2	0.00	-0.01	0.00	0.211	0.822	0.00	-0.01	0.00	0.267	0.936	0.00	0.00	0.00	0.900	0.976	0.00	0.00	0.00	0.751	0.867
Maternal age, years	0.00	0.00	0.00	0.839	0.926	0.00	0.00	0.00	0.965	0.979	0.00	0.00	0.00	0.379	0.537	0.00	0.00	0.00	0.521	0.995
Paternal age, years	0.00	0.00	0.00	0.275	0.621	0.00	0.00	0.00	0.353	0.902	0.00	0.00	0.00	0.076	0.202	0.00	0.00	0.00	0.410	0.694
Maternal smoking, yes vs no	-0.03	-0.07	0.01	0.199	0.398	-0.02	-0.06	0.03	0.393	0.664	-0.03	-0.08	0.02	0.295	0.677	0.02	-0.04	0.07	0.517	0.741
Paternal smoking, yes vs no	-0.02	-0.05	0.01	0.222	0.710	0.00	-0.03	0.03	0.913	0.952	-0.04	-0.08	0.00	0.040	0.428	-0.04	-0.08	0.00	0.058	0.581
**n**						363										282				
**ApoB/ApoA1 (log)**																				
Maternal metabolite (log)	0.10	0.03	0.17	0.008	0.035	0.08	0.01	0.16	0.029	0.116	0.10	0.00	0.20	0.052	0.166	0.06	-0.04	0.16	0.265	0.627
Paternal metabolite (log)	0.00	-0.08	0.08	0.963	0.993	0.00	-0.08	0.08	0.973	0.975	0.17	0.08	0.27	0.000	0.032	0.18	0.08	0.28	0.001	0.035
Newborn sex, females vs males	0.00	-0.04	0.03	0.782	0.834	0.00	-0.04	0.03	0.894	0.906	0.03	-0.01	0.07	0.204	0.493	0.03	-0.01	0.07	0.196	0.349
Newborn birth weight, kg	0.00	-0.03	0.03	0.918	0.948	-0.01	-0.04	0.02	0.536	0.625	-0.03	-0.07	0.01	0.128	0.241	-0.03	-0.08	0.01	0.097	0.231
Maternal BMI, kg/m^2	0.00	0.00	0.01	0.055	0.251	0.00	0.00	0.01	0.153	0.485	0.00	0.00	0.01	0.556	0.932	0.00	0.00	0.01	0.409	0.792
Paternal BMI, kg/m^2	0.00	0.00	0.01	0.634	0.892	0.00	-0.01	0.00	0.928	0.974	0.00	0.00	0.01	0.842	0.946	0.00	-0.01	0.00	0.421	0.867
Maternal age, years	0.00	-0.01	0.00	0.036	0.240	0.00	-0.01	0.00	0.128	0.468	0.00	-0.01	0.00	0.157	0.411	0.00	-0.01	0.00	0.763	0.995
Paternal age, years	0.00	-0.01	0.00	0.186	0.487	0.00	-0.01	0.00	0.765	0.907	0.00	-0.01	0.00	0.088	0.224	0.00	-0.01	0.00	0.055	0.196
Maternal smoking, yes vs no	0.06	0.00	0.13	0.053	0.234	0.05	-0.01	0.12	0.111	0.362	0.05	-0.02	0.13	0.163	0.471	0.05	-0.03	0.14	0.223	0.458
Paternal smoking, yes vs no	0.02	-0.02	0.07	0.320	0.749	0.00	-0.05	0.04	0.868	0.952	0.02	-0.03	0.08	0.416	0.659	0.01	-0.05	0.07	0.714	0.899
**n**						363										282				
**Tot FA, mmol/l (log)**																				
Maternal metabolite (log)	0.07	-0.05	0.20	0.269	0.465	0.04	-0.10	0.17	0.595	0.827	0.04	-0.10	0.18	0.603	0.787	-0.04	-0.18	0.11	0.623	0.801
Paternal metabolite (log)	0.06	-0.03	0.15	0.194	0.830	0.07	-0.03	0.16	0.172	0.803	0.09	-0.03	0.20	0.133	0.371	0.11	-0.01	0.23	0.071	0.284
Newborn sex, females vs males	0.01	-0.04	0.05	0.708	0.795	0.01	-0.03	0.06	0.642	0.781	0.03	-0.02	0.09	0.229	0.506	0.04	-0.01	0.10	0.142	0.293
Newborn birth weight, kg	-0.03	-0.07	0.02	0.223	0.396	-0.03	-0.07	0.01	0.206	0.346	0.01	-0.04	0.06	0.586	0.695	0.02	-0.04	0.07	0.537	0.675
Maternal BMI, kg/m^2	0.00	0.00	0.01	0.125	0.348	0.00	0.00	0.01	0.156	0.485	0.01	0.00	0.01	0.045	0.574	0.01	0.00	0.01	0.033	0.634

Paternal BMI, kg/m^2	0.00	-0.01	0.01	0.703	0.892	0.00	-0.01	0.01	0.606	0.936	0.00	-0.01	0.01	0.922	0.979	0.00	-0.01	0.00	0.301	0.867
Maternal age, years	0.00	-0.01	0.00	0.244	0.541	-0.01	-0.01	0.00	0.145	0.468	0.00	-0.01	0.00	0.192	0.411	0.00	-0.01	0.01	0.892	0.995
Paternal age, years	0.00	-0.01	0.00	0.798	0.837	0.00	-0.01	0.01	0.734	0.902	-0.01	-0.01	0.00	0.012	0.063	-0.01	-0.01	0.00	0.017	0.135
Maternal smoking, yes vs no	0.04	-0.04	0.12	0.362	0.551	0.02	-0.07	0.11	0.657	0.858	0.02	-0.08	0.13	0.684	0.858	0.07	-0.04	0.19	0.222	0.458
Paternal smoking, yes vs no	0.04	-0.02	0.09	0.217	0.710	0.02	-0.05	0.08	0.637	0.952	-0.03	-0.11	0.05	0.431	0.659	-0.02	-0.10	0.06	0.629	0.899
**n**						346										280				
**Omega-3 (%)**																				
Maternal metabolite	-0.02	-0.36	0.33	0.924	0.924	0.05	-0.32	0.43	0.785	0.892	0.11	-0.23	0.45	0.515	0.755	0.21	-0.16	0.57	0.264	0.627
Paternal metabolite	0.00	-0.25	0.25	0.993	0.993	-0.08	-0.35	0.20	0.594	0.955	0.14	-0.12	0.40	0.303	0.571	0.16	-0.13	0.44	0.274	0.568
Newborn sex, females vs males	0.32	-0.18	0.82	0.216	0.329	0.43	-0.09	0.94	0.109	0.194	0.37	-0.17	0.92	0.178	0.493	0.46	-0.10	1.01	0.108	0.247
Newborn birth weight, kg	-0.06	-0.53	0.41	0.793	0.877	0.05	-0.44	0.54	0.840	0.911	0.20	-0.30	0.70	0.440	0.599	0.15	-0.40	0.69	0.598	0.722
Maternal BMI, kg/m^2	-0.02	-0.07	0.03	0.516	0.697	-0.02	-0.08	0.04	0.478	0.800	0.04	-0.02	0.10	0.201	0.761	0.07	0.01	0.14	0.024	0.634
Paternal BMI, kg/m^2	-0.04	-0.12	0.03	0.263	0.822	-0.03	-0.11	0.05	0.450	0.936	-0.04	-0.11	0.03	0.238	0.945	-0.07	-0.14	0.01	0.083	0.781
Maternal age, years	0.00	-0.05	0.06	0.905	0.974	-0.02	-0.10	0.06	0.626	0.770	-0.05	-0.11	0.01	0.123	0.393	-0.06	-0.13	0.02	0.130	0.915
Paternal age, years	0.01	-0.04	0.07	0.612	0.791	0.03	-0.05	0.10	0.457	0.902	0.00	-0.04	0.04	0.934	0.975	0.02	-0.03	0.07	0.392	0.694
Maternal smoking, yes vs no	1.08	0.12	2.05	0.028	0.207	1.14	0.10	2.18	0.032	0.362	0.93	-0.12	1.98	0.085	0.387	0.80	-0.39	1.99	0.187	0.452
Paternal smoking, yes vs no	0.27	-0.40	0.94	0.428	0.831	-0.03	-0.77	0.72	0.939	0.952	0.31	-0.46	1.08	0.432	0.659	0.15	-0.66	0.97	0.712	0.899
**n**						346										280				
**Unsaturation, degree**																				
Maternal metabolite	0.32	0.17	0.47	0.000	0.001	0.32	0.16	0.48	0.000	0.003	0.28	0.13	0.43	0.000	0.007	0.24	0.08	0.41	0.004	0.057
Paternal metabolite	0.01	-0.10	0.12	0.870	0.989	-0.10	-0.22	0.01	0.088	0.743	0.13	0.02	0.25	0.024	0.219	0.09	-0.04	0.21	0.187	0.459
Newborn sex, females vs males	0.01	0.00	0.02	0.147	0.265	0.02	0.00	0.03	0.033	0.095	0.00	-0.01	0.02	0.683	0.766	0.01	-0.01	0.02	0.329	0.478
Newborn birth weight, kg	0.01	-0.01	0.02	0.351	0.535	0.01	0.00	0.03	0.041	0.106	0.01	0.00	0.03	0.177	0.298	0.01	-0.01	0.03	0.184	0.355
Maternal BMI, kg/m^2	0.00	0.00	0.00	0.003	0.045	0.00	0.00	0.00	0.131	0.485	0.00	0.00	0.00	0.275	0.811	0.00	0.00	0.00	0.887	0.976
Paternal BMI, kg/m^2	0.00	0.00	0.00	0.116	0.822	0.00	0.00	0.00	0.348	0.936	0.00	0.00	0.00	0.133	0.945	0.00	0.00	0.00	0.738	0.867
Maternal age, years	0.00	0.00	0.00	0.319	0.567	0.00	0.00	0.00	0.497	0.685	0.00	0.00	0.00	0.060	0.343	0.00	0.00	0.00	0.519	0.995
Paternal age, years	0.00	0.00	0.00	0.134	0.487	0.00	0.00	0.00	0.697	0.902	0.00	0.00	0.00	0.091	0.224	0.00	0.00	0.00	0.058	0.196
Maternal smoking, yes vs no	-0.02	-0.05	0.00	0.101	0.299	-0.01	-0.04	0.01	0.344	0.664	0.01	-0.02	0.05	0.434	0.842	0.01	-0.02	0.05	0.548	0.754
Paternal smoking, yes vs no	-0.01	-0.03	0.01	0.291	0.749	0.00	-0.02	0.02	0.795	0.952	-0.01	-0.04	0.01	0.211	0.586	-0.01	-0.04	0.01	0.296	0.633
**n**						346										280				
**Omega-6 (%)**																				
Maternal metabolite	0.15	0.02	0.27	0.024	0.080	0.16	0.02	0.30	0.023	0.107	0.11	-0.06	0.28	0.192	0.418	0.13	-0.04	0.31	0.142	0.477
Paternal metabolite	0.03	-0.07	0.13	0.591	0.923	-0.02	-0.13	0.09	0.787	0.955	0.02	-0.08	0.12	0.670	0.927	0.01	-0.09	0.12	0.808	0.872
Newborn sex, females vs males	0.87	0.38	1.37	0.001	0.006	0.95	0.45	1.45	0.000	0.002	0.33	-0.30	0.95	0.309	0.530	0.46	-0.15	1.07	0.138	0.293
Newborn birth weight, kg	0.57	0.10	1.03	0.018	0.075	0.75	0.28	1.23	0.002	0.015	1.32	0.76	1.88	0.000	0.000	1.31	0.71	1.91	0.000	0.001
Maternal BMI, kg/m^2	-0.02	-0.07	0.03	0.480	0.683	0.01	-0.05	0.06	0.842	0.932	-0.02	-0.08	0.05	0.553	0.932	-0.02	-0.10	0.05	0.520	0.819
Paternal BMI, kg/m^2	-0.02	-0.09	0.06	0.625	0.892	-0.05	-0.13	0.03	0.238	0.936	-0.02	-0.10	0.06	0.592	0.945	0.02	-0.06	0.11	0.620	0.867
Maternal age, years	-0.01	-0.07	0.04	0.650	0.785	-0.03	-0.11	0.06	0.541	0.706	-0.08	-0.15	-0.01	0.023	0.343	-0.10	-0.18	-0.02	0.013	0.285
Paternal age, years	-0.01	-0.06	0.04	0.765	0.837	0.01	-0.06	0.09	0.747	0.902	-0.04	-0.09	0.01	0.100	0.228	0.01	-0.04	0.06	0.731	0.836
Maternal smoking, yes vs no	-0.10	-1.06	0.87	0.843	0.915	0.08	-0.93	1.09	0.874	0.915	-0.59	-1.80	0.61	0.334	0.713	0.71	-0.59	2.02	0.283	0.533
Paternal smoking, yes vs no	-0.34	-1.02	0.33	0.320	0.749	-0.09	-0.81	0.63	0.815	0.952	-0.55	-1.42	0.32	0.215	0.586	-0.76	-1.67	0.14	0.100	0.581
**n**						346										280				
**PUFA (%)**																				
Maternal metabolite	0.09	-0.06	0.23	0.241	0.428	0.07	-0.09	0.24	0.385	0.665	-0.04	-0.23	0.14	0.632	0.804	0.03	-0.16	0.22	0.785	0.801
Paternal metabolite	0.04	-0.07	0.16	0.454	0.830	-0.01	-0.14	0.11	0.860	0.955	-0.01	-0.13	0.10	0.841	0.978	0.00	-0.12	0.12	0.960	0.976
Newborn sex, females vs males	1.06	0.42	1.71	0.001	0.009	1.25	0.58	1.91	0.000	0.002	0.79	0.03	1.56	0.043	0.493	1.02	0.27	1.77	0.008	0.210
Newborn birth weight, kg	0.55	-0.05	1.16	0.073	0.187	0.82	0.19	1.44	0.011	0.048	1.50	0.80	2.20	0.000	0.001	1.38	0.64	2.12	0.000	0.005
Maternal BMI, kg/m^2	-0.04	-0.10	0.03	0.290	0.563	-0.03	-0.11	0.05	0.442	0.800	0.02	-0.06	0.10	0.627	0.932	0.04	-0.05	0.13	0.433	0.792
Paternal BMI, kg/m^2	-0.06	-0.15	0.04	0.247	0.822	-0.07	-0.18	0.04	0.201	0.936	-0.07	-0.17	0.03	0.192	0.945	-0.06	-0.17	0.05	0.262	0.867
Maternal age, years	-0.01	-0.09	0.06	0.745	0.867	-0.04	-0.15	0.06	0.419	0.666	-0.13	-0.22	-0.04	0.005	0.167	-0.15	-0.25	-0.04	0.006	0.177
Paternal age, years	0.00	-0.07	0.07	0.957	0.957	0.03	-0.06	0.13	0.475	0.902	-0.04	-0.10	0.02	0.158	0.298	0.03	-0.04	0.10	0.419	0.694
Maternal smoking, yes vs no	0.99	-0.26	2.24	0.122	0.311	1.22	-0.10	2.54	0.070	0.362	0.30	-1.22	1.82	0.697	0.858	1.24	-0.39	2.86	0.137	0.431
Paternal smoking, yes vs no	-0.06	-0.94	0.81	0.885	0.977	-0.10	-1.05	0.86	0.845	0.952	-0.35	-1.45	0.75	0.534	0.743	-0.67	-1.80	0.46	0.246	0.633

**n**						346										280				
**MUFA (%) (log)**																				
Maternal metabolite (log)	0.29	0.02	0.56	0.036	0.101	0.23	-0.10	0.56	0.175	0.400	0.17	-0.14	0.48	0.287	0.524	0.19	-0.16	0.54	0.296	0.627
Paternal metabolite (log)	0.16	-0.04	0.37	0.117	0.830	0.09	-0.14	0.31	0.443	0.955	-0.08	-0.29	0.13	0.461	0.720	-0.10	-0.33	0.13	0.374	0.628
Newborn sex, females vs males	-0.06	-0.09	-0.03	0.000	0.006	-0.06	-0.09	-0.03	0.001	0.003	-0.01	-0.04	0.03	0.671	0.766	-0.01	-0.05	0.02	0.486	0.622
Newborn birth weight, kg	0.00	-0.03	0.03	0.939	0.954	-0.01	-0.04	0.02	0.401	0.524	-0.04	-0.08	-0.01	0.014	0.054	-0.04	-0.07	0.00	0.044	0.140
Maternal BMI, kg/m^2	0.00	0.00	0.01	0.124	0.348	0.00	0.00	0.00	0.844	0.932	0.00	0.00	0.00	0.741	0.932	0.00	0.00	0.00	0.940	0.976
Paternal BMI, kg/m^2	0.00	0.00	0.01	0.210	0.822	0.00	0.00	0.01	0.204	0.936	0.00	0.00	0.01	0.273	0.945	0.00	0.00	0.01	0.448	0.867
Maternal age, years	0.00	0.00	0.00	0.989	0.989	0.00	0.00	0.01	0.303	0.666	0.00	0.00	0.01	0.044	0.343	0.00	0.00	0.01	0.053	0.570
Paternal age, years	0.00	0.00	0.00	0.471	0.754	0.00	-0.01	0.00	0.121	0.902	0.00	0.00	0.00	0.191	0.350	0.00	0.00	0.00	0.724	0.836
Maternal smoking, yes vs no	0.02	-0.04	0.08	0.549	0.715	0.01	-0.06	0.08	0.747	0.884	0.02	-0.05	0.09	0.531	0.858	-0.03	-0.11	0.05	0.472	0.719
Paternal smoking, yes vs no	0.01	-0.03	0.06	0.526	0.863	0.00	-0.05	0.05	0.936	0.952	0.02	-0.03	0.07	0.400	0.659	0.04	-0.02	0.09	0.186	0.633
**n**						346										280				
**SFA (%)**																				
Maternal metabolite	0.03	-0.19	0.26	0.776	0.829	0.03	-0.20	0.26	0.788	0.892	-0.14	-0.37	0.09	0.236	0.472	-0.15	-0.40	0.10	0.235	0.627
Paternal metabolite	0.03	-0.11	0.17	0.703	0.978	0.00	-0.15	0.15	0.975	0.975	0.01	-0.16	0.17	0.940	0.986	0.04	-0.14	0.22	0.648	0.827
Newborn sex, females vs males	0.28	-0.25	0.81	0.304	0.442	0.13	-0.41	0.68	0.631	0.781	-0.45	-1.04	0.14	0.137	0.493	-0.60	-1.22	0.01	0.054	0.210
Newborn birth weight, kg	-0.59	-1.08	-0.10	0.019	0.078	-0.54	-1.05	-0.03	0.037	0.105	-0.41	-0.96	0.13	0.134	0.245	-0.44	-1.03	0.16	0.153	0.337
Maternal BMI, kg/m^2	-0.03	-0.08	0.03	0.320	0.578	0.00	-0.06	0.06	0.978	0.978	-0.02	-0.08	0.04	0.493	0.932	-0.03	-0.10	0.04	0.425	0.792
Paternal BMI, kg/m^2	-0.03	-0.11	0.04	0.393	0.822	-0.05	-0.14	0.03	0.231	0.936	0.01	-0.06	0.09	0.720	0.945	0.03	-0.06	0.11	0.519	0.867
Maternal age, years	0.02	-0.05	0.08	0.616	0.758	-0.03	-0.12	0.05	0.444	0.666	0.01	-0.06	0.08	0.719	0.794	0.02	-0.06	0.10	0.562	0.995
Paternal age, years	0.04	-0.02	0.09	0.204	0.501	0.07	-0.01	0.14	0.085	0.902	-0.02	-0.07	0.02	0.360	0.549	-0.03	-0.09	0.02	0.233	0.550
Maternal smoking, yes vs no	-1.41	-2.43	-0.40	0.007	0.170	-1.46	-2.54	-0.39	0.008	0.256	-0.91	-2.04	0.22	0.115	0.414	-0.52	-1.81	0.77	0.428	0.684
Paternal smoking, yes vs no	-0.33	-1.04	0.38	0.358	0.764	-0.04	-0.81	0.73	0.918	0.952	-0.38	-1.21	0.44	0.362	0.659	-0.31	-1.21	0.58	0.495	0.812
**n**						346										280				
**LA (%)**																				
Maternal metabolite	0.26	0.08	0.44	0.006	0.034	0.29	0.10	0.48	0.003	0.040	0.31	0.10	0.53	0.005	0.036	0.39	0.17	0.61	0.001	0.021
Paternal metabolite	0.09	-0.06	0.23	0.247	0.830	0.04	-0.11	0.19	0.583	0.955	0.02	-0.12	0.16	0.790	0.953	-0.05	-0.19	0.10	0.547	0.786
Newborn sex, females vs males	0.30	-0.45	1.06	0.429	0.574	0.47	-0.30	1.23	0.232	0.372	0.61	-0.28	1.50	0.183	0.493	0.66	-0.21	1.52	0.140	0.293
Newborn birth weight, kg	0.90	0.21	1.59	0.011	0.068	0.87	0.16	1.58	0.016	0.053	1.46	0.65	2.26	0.000	0.006	1.49	0.64	2.34	0.001	0.007
Maternal BMI, kg/m^2	0.06	-0.01	0.14	0.094	0.335	0.10	0.01	0.18	0.022	0.351	0.04	-0.06	0.13	0.431	0.924	0.06	-0.04	0.16	0.243	0.764
Paternal BMI, kg/m^2	-0.03	-0.14	0.08	0.623	0.892	-0.09	-0.21	0.03	0.129	0.936	-0.03	-0.14	0.09	0.640	0.945	-0.05	-0.17	0.07	0.428	0.867
Maternal age, years	-0.05	-0.13	0.04	0.279	0.543	-0.05	-0.17	0.07	0.395	0.666	-0.19	-0.29	-0.09	0.000	0.012	-0.22	-0.33	-0.10	0.000	0.016
Paternal age, years	-0.03	-0.11	0.05	0.421	0.730	-0.01	-0.12	0.09	0.820	0.912	-0.08	-0.15	-0.01	0.018	0.079	0.00	-0.07	0.08	0.903	0.964
Maternal smoking, yes vs no	1.35	-0.07	2.78	0.064	0.234	1.29	-0.21	2.79	0.093	0.362	0.45	-1.27	2.18	0.607	0.858	1.48	-0.35	3.31	0.114	0.431
Paternal smoking, yes vs no	0.09	-0.91	1.09	0.857	0.962	-0.18	-1.26	0.90	0.746	0.952	-0.17	-1.43	1.08	0.789	0.870	-0.60	-1.88	0.68	0.361	0.679
**n**						346										280				
**DHA (%)**																				
Maternal metabolite	0.38	0.13	0.64	0.003	0.024	0.34	0.07	0.61	0.013	0.084	0.21	-0.05	0.47	0.121	0.299	0.29	0.01	0.57	0.042	0.224
Paternal metabolite	0.08	-0.07	0.24	0.297	0.830	0.05	-0.12	0.21	0.576	0.955	-0.14	-0.33	0.05	0.156	0.384	-0.20	-0.40	0.00	0.055	0.276
Newborn sex, females vs males	0.03	-0.12	0.17	0.705	0.795	0.04	-0.11	0.19	0.579	0.756	0.06	-0.11	0.23	0.471	0.683	0.07	-0.10	0.24	0.412	0.549
Newborn birth weight, kg	-0.17	-0.30	-0.04	0.012	0.068	-0.14	-0.28	0.00	0.045	0.111	0.05	-0.11	0.20	0.552	0.667	0.01	-0.16	0.18	0.892	0.951
Maternal BMI, kg/m^2	-0.01	-0.03	0.00	0.090	0.335	-0.01	-0.02	0.01	0.530	0.800	0.00	-0.02	0.02	0.944	0.994	0.01	-0.01	0.03	0.349	0.764
Paternal BMI, kg/m^2	-0.02	-0.04	0.00	0.126	0.822	-0.01	-0.03	0.02	0.525	0.936	-0.01	-0.04	0.01	0.189	0.945	-0.02	-0.04	0.00	0.118	0.840
Maternal age, years	0.00	-0.02	0.02	0.935	0.974	-0.02	-0.04	0.00	0.122	0.468	0.00	-0.02	0.02	0.903	0.903	-0.01	-0.03	0.02	0.580	0.995
Paternal age, years	0.01	-0.01	0.02	0.281	0.621	0.02	0.00	0.04	0.090	0.902	0.00	-0.01	0.01	0.910	0.975	0.00	-0.01	0.02	0.721	0.836
Maternal smoking, yes vs no	0.13	-0.15	0.41	0.358	0.551	0.20	-0.09	0.49	0.184	0.512	0.09	-0.24	0.41	0.600	0.858	0.34	-0.03	0.70	0.071	0.431
Paternal smoking, yes vs no	0.01	-0.19	0.20	0.943	0.983	-0.02	-0.23	0.19	0.883	0.952	-0.13	-0.36	0.11	0.292	0.659	-0.12	-0.38	0.13	0.335	0.651
**n**						346										280				
**Alanine, mmol/l**																				
Maternal metabolite	0.42	0.04	0.79	0.030	0.096	0.48	0.09	0.87	0.017	0.091	-0.03	-0.45	0.38	0.887	0.930	-0.11	-0.53	0.31	0.594	0.792
Paternal metabolite	0.05	-0.23	0.33	0.742	0.978	0.07	-0.22	0.37	0.624	0.955	0.06	-0.25	0.37	0.720	0.927	0.10	-0.23	0.43	0.553	0.786
Newborn sex, females vs males	-0.02	-0.05	0.01	0.199	0.321	-0.02	-0.05	0.02	0.332	0.499	0.02	-0.02	0.05	0.316	0.530	0.03	0.00	0.07	0.079	0.210

Newborn birth weight, kg	0.02	-0.01	0.05	0.112	0.263	0.03	0.00	0.06	0.098	0.184	0.04	0.01	0.07	0.012	0.050	0.04	0.01	0.08	0.013	0.054
Maternal BMI, kg/m^2	0.00	0.00	0.00	0.781	0.877	0.00	0.00	0.00	0.498	0.800	0.00	0.00	0.00	0.757	0.932	0.00	0.00	0.01	0.468	0.799
Paternal BMI, kg/m^2	0.00	-0.01	0.00	0.292	0.822	0.00	-0.01	0.00	0.346	0.936	0.00	-0.01	0.00	0.797	0.945	0.00	-0.01	0.00	0.572	0.867
Maternal age, years	0.00	-0.01	0.00	0.024	0.215	0.00	-0.01	0.00	0.608	0.764	0.00	-0.01	0.00	0.278	0.447	0.00	-0.01	0.00	0.229	0.915
Paternal age, years	0.00	-0.01	0.00	0.010	0.209	0.00	-0.01	0.00	0.151	0.902	0.00	0.00	0.00	0.778	0.889	0.00	0.00	0.00	0.297	0.634
Maternal smoking, yes vs no	0.06	0.00	0.13	0.066	0.234	0.06	-0.01	0.12	0.107	0.362	0.00	-0.07	0.07	0.994	0.994	0.00	-0.08	0.07	0.958	0.958
Paternal smoking, yes vs no	0.03	-0.02	0.07	0.211	0.710	0.01	-0.04	0.06	0.715	0.952	0.01	-0.04	0.06	0.721	0.855	0.01	-0.04	0.06	0.677	0.899
**n**						363										283				
**Glutamine, mmol/l**																				
Maternal metabolite	0.29	0.14	0.43	0.000	0.002	0.32	0.14	0.49	0.000	0.009	0.14	-0.01	0.28	0.073	0.195	0.08	-0.11	0.27	0.433	0.735
Paternal metabolite	0.11	0.00	0.21	0.043	0.830	-0.01	-0.12	0.10	0.862	0.955	0.09	-0.02	0.21	0.116	0.349	0.07	-0.06	0.21	0.294	0.568
Newborn sex, females vs males	0.01	0.00	0.02	0.143	0.265	0.01	-0.01	0.02	0.488	0.670	0.01	-0.01	0.02	0.207	0.493	0.01	-0.01	0.02	0.362	0.504
Newborn birth weight, kg	-0.02	-0.03	0.00	0.016	0.075	-0.02	-0.03	-0.01	0.005	0.025	-0.01	-0.02	0.01	0.355	0.507	-0.01	-0.02	0.01	0.386	0.548
Maternal BMI, kg/m^2	0.00	0.00	0.00	0.452	0.673	0.00	0.00	0.00	0.237	0.545	0.00	0.00	0.00	0.721	0.932	0.00	0.00	0.00	0.823	0.970
Paternal BMI, kg/m^2	0.00	0.00	0.00	0.684	0.892	0.00	0.00	0.00	0.625	0.936	0.00	0.00	0.00	0.778	0.945	0.00	0.00	0.00	0.583	0.867
Maternal age, years	0.00	0.00	0.00	0.267	0.543	0.00	0.00	0.00	0.712	0.843	0.00	0.00	0.00	0.206	0.411	0.00	0.00	0.00	0.909	0.995
Paternal age, years	0.00	0.00	0.00	0.070	0.487	0.00	0.00	0.00	0.438	0.902	0.00	0.00	0.00	0.011	0.063	0.00	0.00	0.00	0.017	0.135
Maternal smoking, yes vs no	-0.03	-0.06	0.00	0.032	0.207	-0.03	-0.06	0.00	0.072	0.362	-0.01	-0.03	0.02	0.680	0.858	-0.03	-0.07	0.01	0.095	0.431
Paternal smoking, yes vs no	-0.02	-0.03	0.00	0.119	0.589	-0.01	-0.03	0.01	0.395	0.952	-0.02	-0.04	0.00	0.099	0.483	0.00	-0.03	0.02	0.878	0.954
**n**						288										228				
**Histidine, mmol/l**																				
Maternal metabolite	0.15	-0.03	0.33	0.105	0.224	0.09	-0.09	0.28	0.329	0.602	0.13	-0.07	0.33	0.193	0.418	0.11	-0.09	0.32	0.283	0.627
Paternal metabolite	0.08	-0.08	0.25	0.311	0.830	0.01	-0.16	0.18	0.870	0.955	0.01	-0.16	0.19	0.882	0.986	0.03	-0.16	0.21	0.786	0.872
Newborn sex, females vs males	0.00	0.00	0.00	0.118	0.248	0.00	0.00	0.01	0.048	0.115	0.00	0.00	0.01	0.141	0.493	0.00	0.00	0.01	0.044	0.210
Newborn birth weight, kg	0.00	0.00	0.01	0.062	0.166	0.00	0.00	0.01	0.002	0.015	0.00	0.00	0.01	0.001	0.011	0.00	0.00	0.01	0.002	0.018
Maternal BMI, kg/m^2	0.00	0.00	0.00	0.003	0.045	0.00	0.00	0.00	0.000	0.032	0.00	0.00	0.00	0.202	0.761	0.00	0.00	0.00	0.119	0.634
Paternal BMI, kg/m^2	0.00	0.00	0.00	0.912	0.973	0.00	0.00	0.00	0.627	0.936	0.00	0.00	0.00	0.783	0.945	0.00	0.00	0.00	0.215	0.867
Maternal age, years	0.00	0.00	0.00	0.310	0.567	0.00	0.00	0.00	0.246	0.604	0.00	0.00	0.00	0.592	0.702	0.00	0.00	0.00	0.990	0.995
Paternal age, years	0.00	0.00	0.00	0.634	0.791	0.00	0.00	0.00	0.364	0.902	0.00	0.00	0.00	0.497	0.677	0.00	0.00	0.00	0.842	0.929
Maternal smoking, yes vs no	-0.01	-0.01	0.00	0.014	0.193	-0.01	-0.01	0.00	0.031	0.362	0.01	0.00	0.01	0.072	0.355	0.01	0.00	0.02	0.007	0.361
Paternal smoking, yes vs no	0.00	-0.01	0.00	0.383	0.791	0.00	0.00	0.00	0.707	0.952	0.00	-0.01	0.00	0.220	0.586	0.00	-0.01	0.00	0.076	0.581
**n**						358										280				
**Isoleucine, mmol/l**																				
Maternal metabolite	0.14	0.06	0.22	0.000	0.005	0.11	0.03	0.19	0.010	0.079	0.11	0.01	0.21	0.027	0.109	0.08	-0.02	0.19	0.111	0.443
Paternal metabolite	0.03	-0.03	0.09	0.404	0.830	-0.01	-0.07	0.06	0.800	0.955	-0.01	-0.06	0.05	0.855	0.978	-0.04	-0.10	0.02	0.206	0.488
Newborn sex, females vs males	0.00	0.00	0.00	0.587	0.723	0.00	0.00	0.00	0.498	0.670	0.00	0.00	0.00	0.252	0.509	0.00	-0.01	0.00	0.044	0.210
Newborn birth weight, kg	0.00	0.00	0.00	0.489	0.653	0.00	0.00	0.00	0.185	0.324	0.00	0.00	0.00	0.877	0.920	0.00	0.00	0.00	0.962	0.978
Maternal BMI, kg/m^2	0.00	0.00	0.00	0.000	0.005	0.00	0.00	0.00	0.008	0.240	0.00	0.00	0.00	0.068	0.623	0.00	0.00	0.00	0.116	0.634
Paternal BMI, kg/m^2	0.00	0.00	0.00	0.017	0.822	0.00	0.00	0.00	0.175	0.936	0.00	0.00	0.00	0.014	0.173	0.00	0.00	0.00	0.077	0.781
Maternal age, years	0.00	0.00	0.00	0.067	0.274	0.00	0.00	0.00	0.032	0.468	0.00	0.00	0.00	0.075	0.343	0.00	0.00	0.00	0.215	0.915
Paternal age, years	0.00	0.00	0.00	0.790	0.837	0.00	0.00	0.00	0.358	0.902	0.00	0.00	0.00	0.336	0.524	0.00	0.00	0.00	0.330	0.660
Maternal smoking, yes vs no	0.00	0.00	0.01	0.022	0.207	0.00	0.00	0.01	0.154	0.469	0.00	0.00	0.00	0.905	0.961	0.00	-0.01	0.00	0.950	0.958
Paternal smoking, yes vs no	0.00	0.00	0.01	0.013	0.170	0.00	0.00	0.00	0.452	0.952	0.00	0.00	0.00	0.397	0.659	0.00	0.00	0.01	0.279	0.633
**n**						362										282				
**Leucine, mmol/l**																				
Maternal metabolite	0.16	0.05	0.26	0.003	0.024	0.13	0.02	0.24	0.016	0.091	0.21	0.08	0.33	0.001	0.016	0.14	0.01	0.27	0.037	0.214
Paternal metabolite	0.04	-0.04	0.11	0.327	0.830	0.01	-0.07	0.09	0.768	0.955	0.01	-0.06	0.09	0.739	0.927	-0.02	-0.10	0.06	0.600	0.819
Newborn sex, females vs males	0.00	0.00	0.00	0.803	0.842	0.00	0.00	0.00	0.684	0.781	0.00	-0.01	0.00	0.141	0.493	0.00	-0.01	0.00	0.047	0.210
Newborn birth weight, kg	0.00	0.00	0.00	0.289	0.475	0.00	0.00	0.00	0.187	0.324	0.00	0.00	0.00	0.371	0.516	0.00	0.00	0.00	0.671	0.775
Maternal BMI, kg/m^2	0.00	0.00	0.00	0.008	0.053	0.00	0.00	0.00	0.066	0.485	0.00	0.00	0.00	0.024	0.574	0.00	0.00	0.00	0.096	0.634
Paternal BMI, kg/m^2	0.00	0.00	0.00	0.056	0.822	0.00	0.00	0.00	0.310	0.936	0.00	0.00	0.00	0.004	0.130	0.00	0.00	0.00	0.063	0.781
Maternal age, years	0.00	0.00	0.00	0.019	0.204	0.00	0.00	0.00	0.039	0.468	0.00	0.00	0.00	0.084	0.343	0.00	0.00	0.00	0.226	0.915
Paternal age, years	0.00	0.00	0.00	0.307	0.634	0.00	0.00	0.00	0.690	0.902	0.00	0.00	0.00	0.619	0.748	0.00	0.00	0.00	0.523	0.778

Maternal smoking, yes vs no	0.00	0.00	0.01	0.228	0.442	0.00	0.00	0.01	0.649	0.858	0.00	-0.01	0.01	0.670	0.858	0.00	-0.01	0.01	0.854	0.911
Paternal smoking, yes vs no	0.00	0.00	0.01	0.025	0.227	0.00	0.00	0.01	0.292	0.952	0.00	0.00	0.01	0.421	0.659	0.00	0.00	0.01	0.275	0.633
**n**						363										283				
**Valine, mmol/l**																				
Maternal metabolite	0.15	0.05	0.25	0.004	0.024	0.13	0.03	0.23	0.013	0.084	0.22	0.11	0.34	0.000	0.005	0.15	0.04	0.27	0.011	0.084
Paternal metabolite	0.04	-0.03	0.12	0.259	0.830	0.02	-0.06	0.11	0.566	0.955	0.06	-0.04	0.15	0.227	0.469	0.01	-0.08	0.11	0.802	0.872
Newborn sex, females vs males	0.00	0.00	0.01	0.834	0.861	0.00	-0.01	0.01	0.906	0.906	-0.01	-0.01	0.00	0.099	0.493	-0.01	-0.01	0.00	0.051	0.210
Newborn birth weight, kg	0.00	-0.01	0.00	0.330	0.515	0.00	-0.01	0.00	0.241	0.359	-0.01	-0.01	0.00	0.069	0.158	0.00	-0.01	0.00	0.408	0.568
Maternal BMI, kg/m^2	0.00	0.00	0.00	0.004	0.045	0.00	0.00	0.00	0.017	0.351	0.00	0.00	0.00	0.032	0.574	0.00	0.00	0.00	0.103	0.634
Paternal BMI, kg/m^2	0.00	0.00	0.00	0.062	0.822	0.00	0.00	0.00	0.477	0.936	0.00	0.00	0.00	0.003	0.130	0.00	0.00	0.00	0.077	0.781
Maternal age, years	0.00	0.00	0.00	0.067	0.274	0.00	0.00	0.00	0.009	0.468	0.00	0.00	0.00	0.183	0.411	0.00	0.00	0.00	0.155	0.915
Paternal age, years	0.00	0.00	0.00	0.698	0.828	0.00	0.00	0.00	0.058	0.902	0.00	0.00	0.00	0.981	0.981	0.00	0.00	0.00	0.875	0.949
Maternal smoking, yes vs no	0.00	-0.01	0.01	0.901	0.925	0.00	-0.02	0.01	0.436	0.664	0.00	-0.01	0.01	0.962	0.977	0.00	-0.01	0.02	0.738	0.814
Paternal smoking, yes vs no	0.01	0.00	0.02	0.011	0.170	0.01	0.00	0.02	0.044	0.936	0.00	-0.01	0.01	0.931	0.933	0.00	-0.01	0.01	0.752	0.899
**n**						363										282				
**Phenylalanine, mmol/l**																				
Maternal metabolite	0.17	0.00	0.34	0.046	0.123	0.20	0.02	0.38	0.029	0.116	0.22	0.04	0.40	0.016	0.081	0.14	-0.05	0.34	0.152	0.488
Paternal metabolite	0.06	-0.10	0.23	0.452	0.830	0.05	-0.12	0.22	0.559	0.955	0.00	-0.18	0.19	0.986	0.986	-0.04	-0.24	0.15	0.665	0.827
Newborn sex, females vs males	0.00	0.00	0.00	0.975	0.975	0.00	0.00	0.00	0.682	0.781	0.00	0.00	0.00	0.912	0.941	0.00	0.00	0.00	0.917	0.971
Newborn birth weight, kg	0.00	0.00	0.00	0.141	0.294	0.00	0.00	0.00	0.228	0.354	0.00	0.00	0.00	0.706	0.806	0.00	0.00	0.00	0.506	0.675
Maternal BMI, kg/m^2	0.00	0.00	0.00	0.744	0.850	0.00	0.00	0.00	0.260	0.554	0.00	0.00	0.00	0.157	0.670	0.00	0.00	0.00	0.370	0.764
Paternal BMI, kg/m^2	0.00	0.00	0.00	0.699	0.892	0.00	0.00	0.00	0.611	0.936	0.00	0.00	0.00	0.030	0.316	0.00	0.00	0.00	0.129	0.840
Maternal age, years	0.00	0.00	0.00	0.362	0.594	0.00	0.00	0.00	0.835	0.905	0.00	0.00	0.00	0.649	0.729	0.00	0.00	0.00	0.969	0.995
Paternal age, years	0.00	0.00	0.00	0.299	0.634	0.00	0.00	0.00	0.351	0.902	0.00	0.00	0.00	0.804	0.903	0.00	0.00	0.00	0.803	0.902
Maternal smoking, yes vs no	0.00	0.00	0.01	0.910	0.925	0.00	-0.01	0.01	0.905	0.915	-0.01	-0.01	0.00	0.041	0.265	-0.01	-0.01	0.00	0.078	0.431
Paternal smoking, yes vs no	0.00	0.00	0.00	0.571	0.877	0.00	0.00	0.00	0.865	0.952	0.00	0.00	0.01	0.508	0.722	0.00	0.00	0.01	0.278	0.633
**n**						359										280				
**Tyrosine, mmol/l**																				
Maternal metabolite	0.06	-0.06	0.17	0.331	0.543	0.05	-0.07	0.16	0.450	0.758	0.21	0.08	0.34	0.001	0.016	0.19	0.05	0.33	0.006	0.069
Paternal metabolite	-0.02	-0.11	0.07	0.667	0.978	0.03	-0.07	0.12	0.602	0.955	0.02	-0.09	0.13	0.760	0.936	-0.03	-0.15	0.09	0.601	0.819
Newborn sex, females vs males	0.00	0.00	0.00	0.568	0.713	0.00	0.00	0.00	0.707	0.781	0.00	-0.01	0.00	0.026	0.493	0.00	-0.01	0.00	0.063	0.210
Newborn birth weight, kg	0.00	0.00	0.00	0.289	0.475	0.00	0.00	0.00	0.221	0.353	0.00	0.00	0.00	0.612	0.712	0.00	0.00	0.00	0.975	0.978
Maternal BMI, kg/m^2	0.00	0.00	0.00	0.871	0.945	0.00	0.00	0.00	0.230	0.545	0.00	0.00	0.00	0.148	0.670	0.00	0.00	0.00	0.310	0.764
Paternal BMI, kg/m^2	0.00	0.00	0.00	0.049	0.822	0.00	0.00	0.00	0.017	0.936	0.00	0.00	0.00	0.006	0.130	0.00	0.00	0.00	0.024	0.757
Maternal age, years	0.00	0.00	0.00	0.041	0.241	0.00	0.00	0.00	0.149	0.468	0.00	0.00	0.00	0.418	0.546	0.00	0.00	0.00	0.879	0.995
Paternal age, years	0.00	0.00	0.00	0.107	0.487	0.00	0.00	0.00	0.857	0.912	0.00	0.00	0.00	0.509	0.679	0.00	0.00	0.00	0.588	0.778
Maternal smoking, yes vs no	0.00	0.00	0.01	0.558	0.715	0.00	0.00	0.01	0.589	0.807	0.00	-0.01	0.00	0.097	0.399	0.00	-0.01	0.00	0.176	0.452
Paternal smoking, yes vs no	0.00	0.00	0.00	0.672	0.917	0.00	0.00	0.00	0.921	0.952	0.00	0.00	0.00	0.923	0.933	0.00	0.00	0.01	0.524	0.838
**n**						359										281				
**Glucose, mmol/l (log)**																				
Maternal metabolite (log)	0.96	0.07	1.85	0.036	0.101	0.92	-0.24	2.08	0.120	0.335	1.01	-0.07	2.10	0.068	0.191	1.11	-0.11	2.33	0.076	0.349
Paternal metabolite (log)	0.77	0.11	1.44	0.023	0.745	0.25	-0.59	1.09	0.559	0.955	-0.07	-0.72	0.59	0.846	0.978	-0.29	-1.05	0.47	0.450	0.702
Newborn sex, females vs males	-0.01	-0.41	0.39	0.966	0.975	-0.04	-0.44	0.37	0.864	0.892	-0.27	-0.70	0.15	0.208	0.493	-0.36	-0.79	0.07	0.100	0.247
Newborn birth weight, kg	-0.26	-0.65	0.13	0.194	0.365	-0.22	-0.62	0.17	0.270	0.383	-0.32	-0.73	0.08	0.122	0.238	-0.27	-0.71	0.17	0.235	0.381
Maternal BMI, kg/m^2	-0.01	-0.05	0.03	0.742	0.850	0.00	-0.05	0.04	0.894	0.951	-0.02	-0.06	0.03	0.497	0.932	-0.02	-0.07	0.03	0.452	0.799
Paternal BMI, kg/m^2	0.02	-0.04	0.07	0.588	0.892	0.01	-0.05	0.07	0.842	0.974	-0.02	-0.07	0.04	0.501	0.945	0.01	-0.06	0.07	0.826	0.904
Maternal age, years	0.01	-0.04	0.06	0.610	0.758	-0.01	-0.08	0.06	0.798	0.883	0.06	0.01	0.11	0.027	0.343	0.04	-0.02	0.10	0.224	0.915
Paternal age, years	0.01	-0.03	0.05	0.643	0.791	0.01	-0.05	0.06	0.848	0.912	0.02	-0.02	0.05	0.302	0.508	0.00	-0.04	0.04	0.955	0.996
Maternal smoking, yes vs no	-1.32	-2.12	-0.51	0.001	0.095	-0.87	-1.72	-0.01	0.047	0.362	-0.43	-1.25	0.39	0.304	0.677	-0.29	-1.18	0.60	0.521	0.741
Paternal smoking, yes vs no	-0.98	-1.52	-0.44	0.000	0.014	-0.85	-1.44	-0.26	0.005	0.160	-0.53	-1.14	0.08	0.088	0.483	-0.49	-1.14	0.16	0.144	0.619
**n**						313										249				
**Lactate, mmol/l**																				
Maternal metabolite	0.74	0.39	1.09	0.000	0.001	0.35	-0.14	0.84	0.167	0.395	0.37	0.04	0.70	0.027	0.109	0.12	-0.42	0.65	0.671	0.801

Paternal metabolite	0.55	0.29	0.81	0.000	0.003	0.33	-0.03	0.70	0.077	0.743	0.31	0.04	0.57	0.024	0.219	0.26	-0.18	0.70	0.243	0.554
Newborn sex, females vs males	-0.20	-0.71	0.31	0.447	0.584	-0.17	-0.68	0.33	0.503	0.670	0.28	-0.21	0.78	0.262	0.509	0.45	-0.05	0.95	0.079	0.210
Newborn birth weight, kg	0.15	-0.33	0.64	0.536	0.686	0.03	-0.44	0.51	0.893	0.937	0.57	0.11	1.02	0.016	0.057	0.59	0.10	1.08	0.020	0.072
Maternal BMI, kg/m^2	0.02	-0.03	0.07	0.472	0.683	0.02	-0.04	0.07	0.550	0.800	0.03	-0.03	0.08	0.339	0.811	0.04	-0.02	0.09	0.228	0.764
Paternal BMI, kg/m^2	0.00	-0.08	0.08	0.994	0.994	0.00	-0.07	0.08	0.910	0.974	0.02	-0.05	0.08	0.597	0.945	-0.02	-0.09	0.05	0.534	0.867
Maternal age, years	-0.12	-0.17	-0.06	0.000	0.009	-0.09	-0.18	-0.01	0.028	0.468	-0.06	-0.12	0.00	0.036	0.343	-0.05	-0.12	0.01	0.121	0.915
Paternal age, years	-0.08	-0.13	-0.03	0.003	0.095	-0.02	-0.09	0.05	0.632	0.902	-0.01	-0.05	0.03	0.747	0.869	0.02	-0.03	0.06	0.434	0.694
Maternal smoking, yes vs no	0.95	-0.06	1.95	0.065	0.234	0.58	-0.46	1.62	0.276	0.632	0.10	-0.85	1.06	0.832	0.944	0.45	-0.60	1.50	0.404	0.662
Paternal smoking, yes vs no	0.89	0.19	1.58	0.013	0.170	0.30	-0.43	1.03	0.426	0.952	0.68	-0.03	1.38	0.062	0.444	0.56	-0.19	1.31	0.145	0.619
**n**						363										283				
**Citrate, mmol/l**																				
Maternal metabolite	0.19	0.01	0.38	0.036	0.101	0.16	-0.04	0.35	0.114	0.331	0.26	0.10	0.43	0.002	0.018	0.25	0.07	0.43	0.006	0.069
Paternal metabolite	0.00	-0.18	0.18	0.982	0.993	-0.03	-0.22	0.16	0.742	0.955	0.07	-0.10	0.24	0.422	0.710	-0.04	-0.22	0.13	0.628	0.827
Newborn sex, females vs males	0.00	-0.01	0.00	0.338	0.471	0.00	-0.01	0.00	0.654	0.781	0.00	0.00	0.01	0.820	0.874	0.00	-0.01	0.01	0.937	0.971
Newborn birth weight, kg	0.01	0.00	0.01	0.030	0.101	0.00	0.00	0.01	0.077	0.168	0.00	-0.01	0.00	0.357	0.507	0.00	-0.01	0.00	0.808	0.891
Maternal BMI, kg/m^2	0.00	0.00	0.00	0.335	0.578	0.00	0.00	0.00	0.842	0.932	0.00	0.00	0.00	0.866	0.994	0.00	0.00	0.00	0.621	0.885
Paternal BMI, kg/m^2	0.00	0.00	0.00	0.885	0.960	0.00	0.00	0.00	0.859	0.974	0.00	0.00	0.00	0.413	0.945	0.00	0.00	0.00	0.491	0.867
Maternal age, years	0.00	0.00	0.00	0.361	0.594	0.00	0.00	0.00	0.066	0.468	0.00	0.00	0.00	0.024	0.343	0.00	0.00	0.00	0.030	0.476
Paternal age, years	0.00	0.00	0.00	0.716	0.833	0.00	0.00	0.00	0.142	0.902	0.00	0.00	0.00	0.569	0.714	0.00	0.00	0.00	0.970	0.996
Maternal smoking, yes vs no	0.00	-0.01	0.01	0.491	0.668	0.00	-0.01	0.02	0.430	0.664	-0.01	-0.02	0.00	0.041	0.265	-0.01	-0.02	0.00	0.143	0.431
Paternal smoking, yes vs no	0.00	-0.01	0.01	0.852	0.962	0.00	-0.01	0.01	0.549	0.952	0.00	-0.01	0.01	0.587	0.767	0.00	-0.01	0.01	0.730	0.899
**n**						353										280				
**Acetate, mmol/l (log)**																				
Maternal metabolite (log)	-0.03	-0.11	0.04	0.375	0.546	-0.08	-0.17	0.01	0.098	0.309	0.00	-0.07	0.07	0.991	0.991	0.05	-0.05	0.15	0.369	0.656
Paternal metabolite (log)	0.04	-0.02	0.10	0.192	0.830	0.08	0.01	0.14	0.023	0.743	-0.02	-0.09	0.04	0.455	0.720	-0.05	-0.14	0.04	0.269	0.568
Newborn sex, females vs males	-0.02	-0.06	0.01	0.181	0.305	-0.03	-0.07	0.00	0.060	0.123	-0.01	-0.05	0.03	0.545	0.729	-0.01	-0.05	0.03	0.725	0.860
Newborn birth weight, kg	0.00	-0.04	0.03	0.841	0.897	0.00	-0.03	0.03	0.950	0.965	0.01	-0.02	0.05	0.485	0.634	0.03	-0.01	0.07	0.144	0.329
Maternal BMI, kg/m^2	0.00	0.00	0.01	0.115	0.348	0.00	0.00	0.01	0.197	0.522	0.00	0.00	0.00	0.876	0.994	0.00	-0.01	0.00	0.292	0.764
Paternal BMI, kg/m^2	0.00	0.00	0.01	0.360	0.822	0.00	0.00	0.01	0.595	0.936	0.01	0.00	0.01	0.012	0.173	0.01	0.00	0.01	0.007	0.461
Maternal age, years	0.00	-0.01	0.00	0.591	0.756	0.00	-0.01	0.01	0.801	0.883	0.00	-0.01	0.00	0.297	0.463	0.00	-0.01	0.00	0.305	0.995
Paternal age, years	0.00	0.00	0.00	0.561	0.756	0.00	-0.01	0.00	0.630	0.902	0.00	0.00	0.00	0.971	0.981	0.00	0.00	0.00	0.609	0.780
Maternal smoking, yes vs no	0.04	-0.03	0.11	0.258	0.471	0.06	-0.01	0.14	0.107	0.362	-0.02	-0.09	0.06	0.679	0.858	0.00	-0.09	0.08	0.958	0.958
Paternal smoking, yes vs no	-0.02	-0.07	0.02	0.328	0.749	-0.04	-0.09	0.01	0.127	0.952	0.01	-0.04	0.07	0.621	0.779	0.01	-0.05	0.07	0.831	0.933
**n**						360										281				
**Acetoacetate, mmol/l (log)**																				
Maternal metabolite (log)	0.05	-0.20	0.29	0.720	0.822	-0.10	-0.36	0.17	0.470	0.771	0.07	-0.21	0.34	0.641	0.804	0.06	-0.23	0.35	0.691	0.801
Paternal metabolite (log)	0.04	-0.18	0.26	0.733	0.978	0.07	-0.17	0.30	0.577	0.955	0.05	-0.15	0.26	0.626	0.911	0.05	-0.17	0.26	0.683	0.827
Newborn sex, females vs males	0.00	-0.01	0.00	0.644	0.763	0.00	-0.01	0.00	0.783	0.849	0.00	0.00	0.01	0.436	0.663	0.00	0.00	0.01	0.536	0.673
Newborn birth weight, kg	0.00	0.00	0.01	0.059	0.164	0.00	0.00	0.01	0.093	0.180	0.00	-0.01	0.00	0.760	0.839	0.00	0.00	0.01	0.843	0.914
Maternal BMI, kg/m^2	0.00	0.00	0.00	0.033	0.172	0.00	0.00	0.00	0.138	0.485	0.00	0.00	0.00	0.240	0.809	0.00	0.00	0.00	0.088	0.634
Paternal BMI, kg/m^2	0.00	0.00	0.00	0.411	0.822	0.00	0.00	0.00	0.781	0.974	0.00	0.00	0.00	0.513	0.945	0.00	0.00	0.00	0.154	0.867
Maternal age, years	0.00	0.00	0.00	0.809	0.918	0.00	0.00	0.00	0.968	0.979	0.00	0.00	0.00	0.105	0.378	0.00	0.00	0.00	0.040	0.513
Paternal age, years	0.00	0.00	0.00	0.680	0.821	0.00	0.00	0.00	0.927	0.956	0.00	0.00	0.00	0.945	0.975	0.00	0.00	0.00	0.658	0.826
Maternal smoking, yes vs no	0.00	-0.01	0.01	0.843	0.915	0.00	-0.01	0.01	0.759	0.884	0.00	-0.01	0.01	0.655	0.858	0.00	-0.01	0.01	0.946	0.958
Paternal smoking, yes vs no	0.00	0.00	0.01	0.323	0.749	0.00	0.00	0.01	0.308	0.952	0.00	-0.01	0.00	0.375	0.659	0.00	-0.01	0.00	0.307	0.633
**n**						360										282				
**bOHbutyrate, mmol/l (log)**																				
Maternal metabolite (log)	0.06	-0.07	0.19	0.375	0.546	-0.02	-0.16	0.13	0.802	0.892	0.06	-0.12	0.23	0.519	0.755	0.10	-0.09	0.28	0.315	0.631
Paternal metabolite (log)	0.14	-0.01	0.29	0.063	0.830	0.14	-0.02	0.30	0.093	0.743	-0.16	-0.35	0.02	0.086	0.331	-0.23	-0.43	-0.03	0.028	0.223
Newborn sex, females vs males	-0.01	-0.07	0.04	0.631	0.762	-0.01	-0.06	0.05	0.851	0.892	0.03	-0.03	0.09	0.309	0.530	0.03	-0.04	0.09	0.428	0.559
Newborn birth weight, kg	0.06	0.01	0.11	0.012	0.068	0.06	0.00	0.11	0.038	0.105	0.00	-0.06	0.05	0.922	0.952	0.04	-0.02	0.10	0.191	0.355
Maternal BMI, kg/m^2	0.01	0.00	0.01	0.008	0.053	0.01	0.00	0.01	0.033	0.351	0.00	0.00	0.01	0.452	0.932	0.00	0.00	0.01	0.534	0.819
Paternal BMI, kg/m^2	0.00	0.00	0.01	0.437	0.847	0.00	-0.01	0.01	0.971	0.986	0.00	-0.01	0.01	0.664	0.945	0.00	-0.01	0.01	0.722	0.867

Maternal age, years	0.00	-0.01	0.00	0.536	0.702	0.00	-0.01	0.01	0.734	0.851	0.00	-0.01	0.00	0.247	0.433	-0.01	-0.01	0.00	0.112	0.915
Paternal age, years	0.00	-0.01	0.00	0.541	0.756	0.00	-0.01	0.01	0.629	0.902	0.00	-0.01	0.00	0.430	0.625	0.00	-0.01	0.00	0.732	0.836
Maternal smoking, yes vs no	0.03	-0.08	0.14	0.573	0.719	0.01	-0.10	0.12	0.867	0.915	-0.03	-0.14	0.09	0.673	0.858	0.02	-0.11	0.15	0.776	0.842
Paternal smoking, yes vs no	0.03	-0.05	0.10	0.479	0.855	0.01	-0.07	0.09	0.828	0.952	-0.03	-0.12	0.05	0.459	0.684	-0.02	-0.12	0.07	0.654	0.899
**n**						361										277				
**Creatinine, mmol/l**																				
Maternal metabolite	0.49	0.31	0.66	0.000	0.000	0.49	0.31	0.66	0.000	0.000	0.49	0.25	0.73	0.000	0.005	0.50	0.23	0.76	0.000	0.017
Paternal metabolite	0.01	-0.13	0.14	0.927	0.993	-0.02	-0.15	0.11	0.762	0.955	0.00	-0.07	0.07	0.958	0.986	0.01	-0.06	0.08	0.817	0.872
Newborn sex, females vs males	0.00	0.00	0.00	0.007	0.029	0.00	0.00	0.00	0.018	0.062	0.00	0.00	0.00	0.999	0.999	0.00	0.00	0.00	0.996	0.996
Newborn birth weight, kg	0.00	0.00	0.00	0.443	0.616	0.00	0.00	0.00	0.488	0.600	0.00	0.00	0.00	0.100	0.206	0.00	0.00	0.00	0.191	0.355
Maternal BMI, kg/m^2	0.00	0.00	0.00	0.539	0.697	0.00	0.00	0.00	0.907	0.951	0.00	0.00	0.00	0.632	0.932	0.00	0.00	0.00	0.931	0.976
Paternal BMI, kg/m^2	0.00	0.00	0.00	0.983	0.994	0.00	0.00	0.00	0.944	0.974	0.00	0.00	0.00	0.261	0.945	0.00	0.00	0.00	0.231	0.867
Maternal age, years	0.00	0.00	0.00	0.281	0.543	0.00	0.00	0.00	0.293	0.666	0.00	0.00	0.00	0.850	0.869	0.00	0.00	0.00	0.867	0.995
Paternal age, years	0.00	0.00	0.00	0.759	0.837	0.00	0.00	0.00	0.411	0.902	0.00	0.00	0.00	0.535	0.686	0.00	0.00	0.00	0.572	0.778
Maternal smoking, yes vs no	0.00	-0.01	0.00	0.036	0.207	0.00	-0.01	0.00	0.217	0.550	0.00	-0.01	0.00	0.116	0.414	0.00	-0.01	0.00	0.579	0.754
Paternal smoking, yes vs no	-0.01	-0.01	0.00	0.000	0.011	0.00	-0.01	0.00	0.004	0.160	0.00	-0.01	0.00	0.604	0.774	0.00	0.00	0.00	0.952	0.965
**n**						357										279				
**Albumin, signal area**																				
Maternal metabolite	0.03	-0.16	0.22	0.777	0.829	0.11	-0.10	0.31	0.297	0.560	0.06	-0.14	0.26	0.569	0.787	0.06	-0.14	0.26	0.546	0.792
Paternal metabolite	-0.03	-0.20	0.14	0.720	0.978	-0.09	-0.27	0.10	0.352	0.955	-0.04	-0.21	0.13	0.672	0.927	-0.09	-0.26	0.08	0.311	0.568
Newborn sex, females vs males	0.00	0.00	0.00	0.149	0.265	0.00	0.00	0.00	0.051	0.115	0.00	0.00	0.00	0.119	0.493	0.00	0.00	0.00	0.021	0.210
Newborn birth weight, kg	0.00	0.00	0.00	0.002	0.043	0.00	0.00	0.00	0.001	0.009	0.00	0.00	0.01	0.000	0.000	0.00	0.00	0.01	0.000	0.001
Maternal BMI, kg/m^2	0.00	0.00	0.00	0.936	0.951	0.00	0.00	0.00	0.529	0.800	0.00	0.00	0.00	0.409	0.924	0.00	0.00	0.00	0.551	0.819
Paternal BMI, kg/m^2	0.00	0.00	0.00	0.740	0.892	0.00	0.00	0.00	0.579	0.936	0.00	0.00	0.00	0.254	0.945	0.00	0.00	0.00	0.260	0.867
Maternal age, years	0.00	0.00	0.00	0.721	0.855	0.00	0.00	0.00	0.413	0.666	0.00	0.00	0.00	0.840	0.869	0.00	0.00	0.00	0.814	0.995
Paternal age, years	0.00	0.00	0.00	0.148	0.487	0.00	0.00	0.00	0.102	0.902	0.00	0.00	0.00	0.324	0.524	0.00	0.00	0.00	0.434	0.694
Maternal smoking, yes vs no	0.00	0.00	0.00	0.880	0.924	0.00	0.00	0.00	0.742	0.884	0.00	0.00	0.00	0.855	0.944	0.00	0.00	0.01	0.105	0.431
Paternal smoking, yes vs no	0.00	0.00	0.00	0.913	0.983	0.00	0.00	0.00	0.754	0.952	0.00	0.00	0.00	0.267	0.634	0.00	0.00	0.00	0.236	0.633
**n**						363										283				
**Glycoprotein acetyls, mmol/l**																				
Maternal metabolite	0.09	0.02	0.15	0.007	0.035	0.05	-0.03	0.12	0.206	0.455	0.08	0.00	0.16	0.042	0.150	0.02	-0.08	0.11	0.744	0.801
Paternal metabolite	0.03	-0.04	0.09	0.403	0.830	0.01	-0.06	0.08	0.719	0.955	0.04	-0.02	0.10	0.181	0.429	0.03	-0.03	0.09	0.383	0.628
Newborn sex, females vs males	-0.02	-0.05	0.01	0.154	0.266	-0.01	-0.04	0.02	0.372	0.541	0.03	0.00	0.06	0.067	0.493	0.03	0.00	0.06	0.074	0.210
Newborn birth weight, kg	0.04	0.02	0.07	0.001	0.043	0.04	0.01	0.06	0.009	0.043	0.04	0.01	0.07	0.005	0.030	0.06	0.03	0.09	0.001	0.007
Maternal BMI, kg/m^2	0.00	0.00	0.01	0.003	0.045	0.00	0.00	0.01	0.159	0.485	0.00	0.00	0.01	0.055	0.590	0.00	0.00	0.01	0.364	0.764
Paternal BMI, kg/m^2	0.00	0.00	0.01	0.635	0.892	0.00	-0.01	0.00	0.666	0.947	0.00	0.00	0.01	0.124	0.945	0.00	0.00	0.01	0.405	0.867
Maternal age, years	0.00	0.00	0.00	0.959	0.974	0.00	0.00	0.01	0.447	0.666	0.00	0.00	0.00	0.855	0.869	0.00	0.00	0.00	0.915	0.995
Paternal age, years	0.00	0.00	0.00	0.357	0.714	0.00	-0.01	0.00	0.140	0.902	0.00	0.00	0.00	0.582	0.717	0.00	0.00	0.00	0.312	0.643
Maternal smoking, yes vs no	0.05	-0.01	0.10	0.091	0.297	0.03	-0.02	0.09	0.262	0.621	0.02	-0.04	0.08	0.526	0.858	0.05	-0.02	0.11	0.183	0.452
Paternal smoking, yes vs no	0.03	-0.01	0.07	0.146	0.624	0.02	-0.02	0.06	0.409	0.952	0.01	-0.04	0.06	0.680	0.837	0.01	-0.04	0.06	0.762	0.899
**n**						363										283				
**VLDL size, nm**																				
Maternal metabolite	0.10	0.02	0.19	0.014	0.049	0.08	-0.01	0.17	0.096	0.309	0.12	0.05	0.20	0.002	0.018	0.07	-0.01	0.16	0.093	0.398
Paternal metabolite	0.03	-0.04	0.11	0.386	0.830	0.01	-0.08	0.09	0.905	0.955	0.07	0.00	0.13	0.043	0.283	0.05	-0.01	0.12	0.119	0.362
Newborn sex, females vs males	-0.13	-0.28	0.02	0.082	0.194	-0.15	-0.30	0.00	0.058	0.123	0.02	-0.13	0.17	0.810	0.874	-0.04	-0.18	0.11	0.638	0.785
Newborn birth weight, kg	-0.11	-0.25	0.03	0.115	0.263	-0.18	-0.32	-0.04	0.013	0.048	-0.15	-0.28	-0.01	0.035	0.109	-0.17	-0.31	-0.02	0.025	0.085
Maternal BMI, kg/m^2	0.02	0.01	0.04	0.006	0.053	0.02	0.00	0.04	0.028	0.351	0.01	0.00	0.03	0.106	0.641	0.01	-0.01	0.03	0.167	0.714
Paternal BMI, kg/m^2	0.01	-0.01	0.03	0.264	0.822	0.01	-0.02	0.03	0.643	0.936	0.01	-0.01	0.03	0.362	0.945	-0.01	-0.03	0.01	0.508	0.867
Maternal age, years	-0.01	-0.03	0.00	0.167	0.483	-0.01	-0.04	0.01	0.321	0.666	-0.02	-0.03	0.00	0.067	0.343	-0.01	-0.03	0.01	0.221	0.915
Paternal age, years	-0.01	-0.02	0.01	0.497	0.756	0.00	-0.02	0.02	0.743	0.902	0.00	-0.02	0.01	0.455	0.633	0.00	-0.02	0.01	0.536	0.778
Maternal smoking, yes vs no	0.23	-0.05	0.52	0.112	0.299	0.25	-0.05	0.55	0.108	0.362	0.40	0.13	0.68	0.004	0.137	0.30	-0.02	0.61	0.064	0.431
Paternal smoking, yes vs no	0.02	-0.18	0.22	0.851	0.962	-0.16	-0.38	0.06	0.147	0.952	0.14	-0.06	0.34	0.172	0.561	0.12	-0.09	0.34	0.272	0.633
**n**						363										282				

**LDL size, nm**																				
Maternal metabolite	0.29	-0.56	1.13	0.506	0.646	0.12	-0.76	1.00	0.796	0.892	0.62	-0.03	1.27	0.064	0.186	0.52	-0.16	1.21	0.134	0.475
Paternal metabolite	0.04	-0.44	0.53	0.864	0.989	0.04	-0.45	0.54	0.862	0.955	0.19	-0.24	0.63	0.388	0.710	0.04	-0.44	0.52	0.866	0.909
Newborn sex, females vs males	0.09	0.00	0.18	0.050	0.144	0.11	0.01	0.20	0.031	0.093	0.06	-0.03	0.16	0.188	0.493	0.07	-0.03	0.17	0.173	0.326
Newborn birth weight, kg	0.00	-0.09	0.09	0.997	0.997	0.00	-0.08	0.09	0.925	0.955	0.04	-0.05	0.13	0.351	0.507	0.04	-0.05	0.14	0.372	0.541
Maternal BMI, kg/m^2	0.01	0.00	0.01	0.257	0.515	0.01	0.00	0.02	0.283	0.584	0.00	-0.01	0.01	0.613	0.932	0.00	-0.01	0.01	0.723	0.945
Paternal BMI, kg/m^2	0.00	-0.01	0.02	0.506	0.892	0.00	-0.01	0.02	0.766	0.974	0.00	-0.01	0.02	0.649	0.945	0.00	-0.01	0.02	0.834	0.904
Maternal age, years	-0.01	-0.02	0.00	0.014	0.174	-0.01	-0.03	0.00	0.113	0.468	-0.01	-0.02	0.00	0.211	0.411	0.00	-0.01	0.01	0.965	0.995
Paternal age, years	-0.01	-0.02	0.00	0.080	0.487	0.00	-0.02	0.01	0.713	0.902	-0.01	-0.02	0.00	0.015	0.075	-0.01	-0.02	0.00	0.016	0.135
Maternal smoking, yes vs no	0.09	-0.08	0.27	0.303	0.525	0.09	-0.10	0.28	0.374	0.664	0.01	-0.17	0.19	0.931	0.961	0.10	-0.11	0.31	0.371	0.642
Paternal smoking, yes vs no	0.05	-0.07	0.17	0.432	0.831	0.02	-0.12	0.15	0.822	0.952	-0.02	-0.16	0.11	0.752	0.859	0.02	-0.13	0.17	0.795	0.908
**n**						363										282				
**HDL size, nm**																				
Maternal metabolite	0.09	-0.05	0.22	0.209	0.394	0.07	-0.07	0.22	0.339	0.603	0.22	0.09	0.36	0.001	0.016	0.22	0.07	0.36	0.003	0.057
Paternal metabolite	0.02	-0.12	0.16	0.734	0.978	0.04	-0.11	0.19	0.579	0.955	0.03	-0.12	0.18	0.715	0.927	-0.02	-0.18	0.13	0.766	0.872
Newborn sex, females vs males	0.05	0.00	0.10	0.072	0.177	0.05	0.00	0.11	0.050	0.115	-0.02	-0.07	0.04	0.569	0.734	0.00	-0.05	0.06	0.967	0.983
Newborn birth weight, kg	0.03	-0.02	0.08	0.203	0.371	0.05	0.00	0.10	0.062	0.147	0.03	-0.02	0.08	0.259	0.415	0.03	-0.02	0.09	0.226	0.381
Maternal BMI, kg/m^2	0.00	-0.01	0.00	0.199	0.451	0.00	-0.01	0.00	0.337	0.654	0.00	-0.01	0.01	0.925	0.994	0.00	-0.01	0.01	0.844	0.970
Paternal BMI, kg/m^2	0.00	-0.01	0.01	0.521	0.892	0.00	-0.01	0.01	0.790	0.974	0.00	-0.01	0.01	0.615	0.945	0.00	-0.01	0.01	0.995	0.995
Maternal age, years	0.00	-0.01	0.00	0.450	0.642	0.00	-0.01	0.01	0.487	0.685	0.00	-0.01	0.00	0.591	0.702	0.00	-0.01	0.01	0.572	0.995
Paternal age, years	0.00	-0.01	0.00	0.539	0.756	0.00	-0.01	0.01	0.828	0.912	0.00	-0.01	0.00	0.118	0.244	0.00	-0.01	0.00	0.427	0.694
Maternal smoking, yes vs no	-0.02	-0.12	0.08	0.752	0.875	-0.01	-0.11	0.10	0.906	0.915	-0.10	-0.20	0.01	0.070	0.355	-0.04	-0.16	0.08	0.493	0.734
Paternal smoking, yes vs no	-0.01	-0.08	0.06	0.785	0.932	0.00	-0.07	0.08	0.924	0.952	-0.06	-0.14	0.02	0.121	0.483	-0.07	-0.15	0.02	0.124	0.619
**n**						363										282				
**XXL VLDL P**																				
Maternal metabolite, nmol/l	0.68	-2.13	3.50	0.634	0.751	0.46	-2.63	3.54	0.771	0.892	0.57	-3.23	4.38	0.767	0.832	-0.84	-5.09	3.41	0.699	0.801
Paternal metabolite, nmol/l	-0.79	-3.24	1.65	0.526	0.885	-0.15	-2.87	2.56	0.911	0.955	-0.22	-1.42	0.97	0.712	0.927	-0.06	-1.33	1.21	0.922	0.952
Newborn sex, females vs males	0.26	-0.15	0.66	0.212	0.329	0.24	-0.19	0.67	0.272	0.424	0.01	-0.43	0.46	0.956	0.971	-0.04	-0.52	0.43	0.857	0.945
Newborn birth weight, kg	-0.07	-0.45	0.30	0.703	0.833	0.00	-0.40	0.40	0.988	0.988	-0.21	-0.63	0.20	0.308	0.469	-0.17	-0.64	0.29	0.464	0.632
Maternal BMI, kg/m^2	-0.01	-0.05	0.03	0.641	0.760	-0.01	-0.06	0.04	0.608	0.828	0.03	-0.01	0.08	0.144	0.670	0.05	0.00	0.11	0.070	0.634
Paternal BMI, kg/m^2	-0.01	-0.07	0.05	0.807	0.939	0.00	-0.06	0.07	0.931	0.974	-0.03	-0.09	0.03	0.308	0.945	-0.05	-0.12	0.02	0.131	0.840
Maternal age, years	-0.07	-0.12	-0.03	0.003	0.082	-0.05	-0.12	0.01	0.121	0.468	-0.01	-0.06	0.04	0.619	0.707	-0.04	-0.10	0.02	0.180	0.915
Paternal age, years	-0.07	-0.11	-0.02	0.003	0.095	-0.04	-0.10	0.02	0.240	0.902	0.00	-0.03	0.03	0.941	0.975	0.00	-0.04	0.04	0.996	0.996
Maternal smoking, yes vs no	0.87	0.08	1.66	0.031	0.207	0.85	-0.02	1.72	0.056	0.362	-0.09	-0.93	0.76	0.841	0.944	0.21	-0.79	1.22	0.676	0.787
Paternal smoking, yes vs no	0.42	-0.12	0.96	0.129	0.589	0.12	-0.50	0.74	0.709	0.952	-0.09	-0.72	0.53	0.770	0.865	0.02	-0.68	0.71	0.965	0.965
**n**						364										283				
**XL VLDL P**																				
Maternal metabolite, nmol/l	0.23	-0.22	0.68	0.321	0.540	0.13	-0.38	0.63	0.627	0.837	0.40	-0.21	1.00	0.196	0.418	0.15	-0.55	0.85	0.679	0.801
Paternal metabolite, nmol/l	0.08	-0.43	0.59	0.763	0.978	-0.08	-0.66	0.50	0.794	0.955	0.04	-0.21	0.30	0.738	0.927	-0.06	-0.34	0.23	0.696	0.827
Newborn sex, females vs males	-0.33	-0.75	0.09	0.120	0.248	-0.40	-0.84	0.05	0.080	0.151	-0.13	-0.57	0.32	0.573	0.734	-0.32	-0.80	0.16	0.195	0.349
Newborn birth weight, kg	-0.13	-0.51	0.25	0.503	0.657	-0.23	-0.64	0.17	0.262	0.381	-0.65	-1.08	-0.21	0.003	0.024	-0.64	-1.12	-0.16	0.009	0.040
Maternal BMI, kg/m^2	0.02	-0.02	0.06	0.343	0.578	0.01	-0.04	0.06	0.627	0.837	0.01	-0.03	0.06	0.605	0.932	0.00	-0.05	0.06	0.951	0.976
Paternal BMI, kg/m^2	0.05	-0.01	0.11	0.093	0.822	0.06	-0.01	0.13	0.096	0.936	0.04	-0.02	0.09	0.191	0.945	0.02	-0.04	0.09	0.510	0.867
Maternal age, years	-0.05	-0.10	0.00	0.049	0.255	-0.03	-0.10	0.04	0.393	0.666	-0.02	-0.07	0.03	0.392	0.537	-0.02	-0.09	0.04	0.472	0.995
Paternal age, years	-0.03	-0.07	0.01	0.177	0.487	-0.02	-0.08	0.04	0.452	0.902	-0.01	-0.04	0.03	0.741	0.869	-0.02	-0.06	0.03	0.483	0.754
Maternal smoking, yes vs no	1.30	0.34	2.26	0.008	0.170	1.44	0.40	2.47	0.006	0.256	0.94	0.06	1.82	0.037	0.265	0.77	-0.29	1.84	0.155	0.431
Paternal smoking, yes vs no	0.19	-0.37	0.75	0.505	0.855	-0.28	-0.92	0.36	0.395	0.952	0.43	-0.19	1.05	0.175	0.561	0.48	-0.22	1.19	0.180	0.633
**n**						364										283				
**L VLDL P**																				
Maternal metabolite, nmol/l	0.06	-0.01	0.14	0.090	0.206	0.05	-0.04	0.13	0.278	0.539	0.11	0.01	0.21	0.032	0.121	0.05	-0.08	0.17	0.459	0.735
Paternal metabolite, nmol/l	0.01	-0.07	0.10	0.790	0.989	-0.03	-0.13	0.06	0.508	0.955	0.07	0.01	0.12	0.014	0.219	0.06	0.00	0.13	0.042	0.264
Newborn sex, females vs males	-0.27	-0.67	0.14	0.201	0.321	-0.37	-0.81	0.07	0.099	0.181	-0.10	-0.54	0.34	0.658	0.766	-0.29	-0.79	0.20	0.247	0.394
Newborn birth weight, kg	-0.37	-0.75	0.01	0.057	0.164	-0.52	-0.94	-0.11	0.014	0.048	-0.92	-1.38	-0.46	0.000	0.001	-0.94	-1.45	-0.43	0.000	0.005

Maternal BMI, kg/m^2	0.05	0.00	0.09	0.035	0.172	0.05	0.00	0.10	0.076	0.485	0.04	-0.01	0.08	0.139	0.670	0.04	-0.02	0.10	0.162	0.714
Paternal BMI, kg/m^2	0.04	-0.01	0.10	0.141	0.822	0.04	-0.03	0.11	0.295	0.936	0.02	-0.03	0.08	0.412	0.945	-0.04	-0.11	0.03	0.301	0.867
Maternal age, years	-0.02	-0.06	0.03	0.458	0.642	-0.04	-0.11	0.02	0.200	0.555	-0.05	-0.10	0.00	0.055	0.343	-0.04	-0.10	0.03	0.295	0.995
Paternal age, years	0.01	-0.03	0.05	0.502	0.756	0.03	-0.03	0.09	0.334	0.902	-0.01	-0.05	0.02	0.413	0.615	-0.02	-0.06	0.02	0.384	0.694
Maternal smoking, yes vs no	0.67	-0.15	1.50	0.111	0.299	0.79	-0.13	1.71	0.092	0.362	1.28	0.33	2.23	0.009	0.137	1.14	-0.08	2.36	0.066	0.431
Paternal smoking, yes vs no	-0.02	-0.56	0.52	0.948	0.983	-0.41	-1.04	0.22	0.204	0.952	0.52	-0.10	1.14	0.102	0.483	0.49	-0.25	1.23	0.197	0.633
**n**						364										283				
**M VLDL P**																				
Maternal metabolite, nmol/l	0.01	-0.01	0.04	0.372	0.546	0.00	-0.02	0.03	0.743	0.892	0.04	0.00	0.07	0.055	0.166	0.01	-0.03	0.05	0.590	0.792
Paternal metabolite, nmol/l	0.00	-0.03	0.04	0.764	0.978	0.00	-0.04	0.03	0.925	0.955	0.02	-0.01	0.04	0.154	0.384	0.01	-0.01	0.04	0.303	0.568
Newborn sex, females vs males	-0.07	-0.48	0.34	0.735	0.798	-0.09	-0.51	0.33	0.679	0.781	0.13	-0.33	0.58	0.589	0.739	0.06	-0.43	0.55	0.815	0.929
Newborn birth weight, kg	-0.28	-0.66	0.10	0.152	0.303	-0.35	-0.75	0.05	0.087	0.175	-0.39	-0.82	0.04	0.074	0.164	-0.31	-0.79	0.17	0.200	0.355
Maternal BMI, kg/m^2	0.03	-0.01	0.08	0.110	0.348	0.04	-0.01	0.08	0.150	0.485	0.03	-0.02	0.08	0.240	0.809	0.03	-0.03	0.08	0.361	0.764
Paternal BMI, kg/m^2	0.03	-0.03	0.08	0.366	0.822	0.02	-0.05	0.09	0.565	0.936	0.01	-0.05	0.07	0.682	0.945	-0.03	-0.09	0.04	0.435	0.867
Maternal age, years	-0.05	-0.09	0.00	0.052	0.255	-0.07	-0.14	0.00	0.047	0.468	-0.03	-0.08	0.02	0.253	0.433	-0.01	-0.08	0.05	0.746	0.995
Paternal age, years	-0.01	-0.05	0.04	0.786	0.837	0.03	-0.03	0.08	0.406	0.902	-0.01	-0.05	0.02	0.445	0.633	-0.02	-0.07	0.02	0.271	0.599
Maternal smoking, yes vs no	0.79	-0.05	1.63	0.066	0.234	0.83	-0.07	1.73	0.072	0.362	0.90	-0.02	1.81	0.055	0.320	0.85	-0.29	1.99	0.144	0.431
Paternal smoking, yes vs no	0.12	-0.43	0.66	0.673	0.917	-0.22	-0.84	0.39	0.473	0.952	0.59	-0.07	1.24	0.078	0.483	0.73	-0.01	1.48	0.054	0.581
**n**						364										283				
**S VLDL P**																				
Maternal metabolite, nmol/l	0.01	-0.01	0.02	0.471	0.642	0.00	-0.02	0.02	0.875	0.934	0.02	-0.01	0.04	0.162	0.385	0.00	-0.03	0.03	0.946	0.946
Paternal metabolite, nmol/l	0.00	-0.02	0.02	0.922	0.993	0.00	-0.03	0.02	0.720	0.955	0.01	-0.01	0.03	0.414	0.710	0.01	-0.01	0.03	0.437	0.700
Newborn sex, females vs males	-0.16	-0.57	0.24	0.431	0.574	-0.16	-0.58	0.27	0.467	0.664	0.16	-0.29	0.60	0.491	0.683	0.02	-0.46	0.50	0.938	0.971
Newborn birth weight, kg	-0.06	-0.43	0.31	0.752	0.860	-0.14	-0.53	0.25	0.492	0.600	-0.40	-0.82	0.01	0.058	0.148	-0.30	-0.77	0.16	0.205	0.355
Maternal BMI, kg/m^2	0.04	0.00	0.08	0.073	0.290	0.04	-0.01	0.09	0.111	0.485	0.03	-0.02	0.07	0.285	0.811	0.03	-0.03	0.09	0.279	0.764
Paternal BMI, kg/m^2	0.02	-0.03	0.08	0.404	0.822	0.02	-0.05	0.08	0.558	0.936	0.01	-0.04	0.07	0.621	0.945	-0.02	-0.09	0.04	0.513	0.867
Maternal age, years	-0.05	-0.10	0.00	0.037	0.240	-0.07	-0.13	0.00	0.051	0.468	-0.03	-0.08	0.02	0.254	0.433	-0.01	-0.07	0.06	0.834	0.995
Paternal age, years	-0.01	-0.05	0.03	0.619	0.791	0.02	-0.04	0.08	0.492	0.902	-0.02	-0.05	0.02	0.284	0.492	-0.03	-0.07	0.01	0.180	0.461
Maternal smoking, yes vs no	0.59	-0.23	1.41	0.157	0.356	0.62	-0.25	1.50	0.161	0.469	1.51	0.47	2.55	0.004	0.137	1.27	0.09	2.45	0.034	0.431
Paternal smoking, yes vs no	0.08	-0.47	0.62	0.786	0.932	-0.18	-0.79	0.42	0.553	0.952	0.50	-0.14	1.13	0.128	0.483	0.54	-0.18	1.26	0.141	0.619
**n**						364										283				
**XS VLDL P**																				
Maternal metabolite, nmol/l	0.01	0.00	0.02	0.220	0.403	0.00	-0.01	0.02	0.497	0.784	0.00	-0.02	0.02	0.935	0.964	-0.01	-0.03	0.01	0.450	0.735
Paternal metabolite, nmol/l	0.01	-0.01	0.02	0.543	0.891	0.00	-0.02	0.02	0.871	0.955	0.02	0.00	0.03	0.062	0.308	0.01	0.00	0.03	0.113	0.362
Newborn sex, females vs males	-0.21	-0.63	0.20	0.311	0.442	-0.21	-0.65	0.22	0.335	0.499	0.08	-0.37	0.53	0.728	0.804	-0.02	-0.51	0.47	0.941	0.971
Newborn birth weight, kg	-0.11	-0.49	0.27	0.560	0.703	-0.13	-0.53	0.27	0.537	0.625	-0.13	-0.54	0.29	0.549	0.667	-0.09	-0.57	0.38	0.697	0.783
Maternal BMI, kg/m^2	0.02	-0.02	0.06	0.376	0.594	0.00	-0.04	0.05	0.922	0.952	0.01	-0.03	0.06	0.604	0.932	0.01	-0.04	0.07	0.623	0.885
Paternal BMI, kg/m^2	0.04	-0.02	0.10	0.182	0.822	0.04	-0.02	0.11	0.223	0.936	0.01	-0.05	0.06	0.816	0.946	-0.02	-0.09	0.05	0.528	0.867
Maternal age, years	-0.03	-0.08	0.01	0.144	0.461	-0.03	-0.10	0.04	0.427	0.666	-0.03	-0.08	0.02	0.279	0.447	0.01	-0.06	0.07	0.818	0.995
Paternal age, years	-0.02	-0.06	0.02	0.401	0.730	-0.01	-0.07	0.05	0.791	0.912	-0.05	-0.08	-0.01	0.011	0.063	-0.08	-0.13	-0.03	0.001	0.063
Maternal smoking, yes vs no	0.92	0.07	1.77	0.035	0.207	0.78	-0.13	1.69	0.095	0.362	0.64	-0.24	1.53	0.152	0.471	0.90	-0.23	2.03	0.117	0.431
Paternal smoking, yes vs no	0.50	-0.06	1.06	0.079	0.507	0.27	-0.35	0.89	0.389	0.952	0.83	0.15	1.51	0.017	0.392	1.14	0.37	1.90	0.004	0.238
**n**						364										283				
**IDL P**																				
Maternal metabolite, nmol/l	0.00	0.00	0.01	0.761	0.829	0.00	-0.01	0.01	0.893	0.937	0.00	-0.01	0.01	0.679	0.808	0.00	-0.01	0.01	0.629	0.801
Paternal metabolite, nmol/l	0.00	0.00	0.01	0.259	0.830	0.01	0.00	0.01	0.150	0.803	0.01	0.00	0.01	0.056	0.300	0.01	0.00	0.01	0.044	0.264
Newborn sex, females vs males	0.53	0.11	0.94	0.013	0.047	0.71	0.26	1.16	0.002	0.009	0.22	-0.22	0.67	0.323	0.530	0.27	-0.21	0.75	0.264	0.394
Newborn birth weight, kg	0.30	-0.08	0.69	0.122	0.270	0.36	-0.05	0.77	0.081	0.168	0.01	-0.40	0.42	0.953	0.959	0.01	-0.46	0.48	0.978	0.978
Maternal BMI, kg/m^2	0.03	-0.01	0.07	0.176	0.433	0.03	-0.02	0.08	0.189	0.522	-0.01	-0.05	0.04	0.789	0.935	-0.01	-0.06	0.04	0.742	0.945
Paternal BMI, kg/m^2	0.01	-0.05	0.07	0.747	0.892	-0.02	-0.08	0.05	0.643	0.936	-0.02	-0.08	0.04	0.504	0.945	-0.02	-0.08	0.04	0.536	0.867
Maternal age, years	-0.04	-0.09	0.01	0.083	0.311	-0.03	-0.10	0.04	0.358	0.666	-0.03	-0.08	0.02	0.256	0.433	-0.01	-0.07	0.06	0.816	0.995
Paternal age, years	-0.04	-0.08	0.01	0.094	0.487	-0.02	-0.08	0.04	0.460	0.902	-0.05	-0.08	-0.01	0.007	0.063	-0.06	-0.10	-0.01	0.011	0.135
Maternal smoking, yes vs no	0.10	-0.69	0.89	0.801	0.915	-0.10	-0.97	0.77	0.822	0.909	-0.20	-1.03	0.64	0.642	0.858	0.26	-0.77	1.29	0.624	0.754

Paternal smoking, yes vs no	0.37	-0.18	0.92	0.187	0.705	0.37	-0.25	0.99	0.243	0.952	-0.26	-0.88	0.36	0.409	0.659	-0.04	-0.74	0.67	0.922	0.965
**n**						364										283				
**L LDL P**																				
Maternal metabolite, nmol/l	0.00	0.00	0.00	0.387	0.551	0.00	0.00	0.00	0.519	0.790	0.00	0.00	0.01	0.550	0.782	0.00	0.00	0.01	0.514	0.783
Paternal metabolite, nmol/l	0.00	0.00	0.01	0.435	0.830	0.00	0.00	0.01	0.200	0.803	0.00	0.00	0.01	0.102	0.345	0.00	0.00	0.01	0.069	0.284
Newborn sex, females vs males	0.62	0.20	1.04	0.004	0.019	0.83	0.37	1.29	0.000	0.003	0.28	-0.17	0.72	0.223	0.506	0.45	-0.04	0.93	0.069	0.210
Newborn birth weight, kg	0.42	0.03	0.81	0.034	0.107	0.53	0.11	0.95	0.013	0.048	0.43	0.01	0.85	0.044	0.123	0.31	-0.16	0.79	0.196	0.355
Maternal BMI, kg/m^2	0.03	-0.01	0.07	0.212	0.451	0.03	-0.02	0.07	0.238	0.545	-0.01	-0.06	0.04	0.658	0.932	-0.01	-0.06	0.05	0.799	0.970
Paternal BMI, kg/m^2	-0.01	-0.06	0.05	0.838	0.941	-0.04	-0.10	0.03	0.297	0.936	-0.02	-0.08	0.04	0.469	0.945	-0.02	-0.08	0.04	0.550	0.867
Maternal age, years	-0.04	-0.08	0.01	0.134	0.450	-0.04	-0.11	0.03	0.212	0.565	-0.04	-0.09	0.01	0.159	0.411	-0.01	-0.07	0.05	0.749	0.995
Paternal age, years	-0.03	-0.07	0.01	0.184	0.487	-0.01	-0.07	0.05	0.845	0.912	-0.05	-0.08	-0.01	0.010	0.063	-0.04	-0.09	0.00	0.050	0.196
Maternal smoking, yes vs no	-0.08	-0.87	0.70	0.835	0.915	-0.24	-1.10	0.63	0.592	0.807	-0.23	-1.06	0.61	0.598	0.858	0.26	-0.76	1.28	0.622	0.754
Paternal smoking, yes vs no	0.21	-0.33	0.76	0.441	0.831	0.27	-0.35	0.89	0.396	0.952	-0.29	-0.91	0.33	0.356	0.659	-0.16	-0.86	0.55	0.658	0.899
**n**						364										283				
**M LDL P**																				
Maternal metabolite, nmol/l	0.00	0.00	0.01	0.204	0.394	0.00	0.00	0.01	0.234	0.479	0.00	0.00	0.01	0.328	0.567	0.00	0.00	0.01	0.273	0.627
Paternal metabolite, nmol/l	0.00	-0.01	0.00	0.834	0.989	0.00	0.00	0.01	0.821	0.955	0.00	0.00	0.01	0.217	0.466	0.00	0.00	0.01	0.134	0.388
Newborn sex, females vs males	0.58	0.16	1.00	0.007	0.029	0.77	0.31	1.24	0.001	0.006	0.23	-0.22	0.67	0.321	0.530	0.40	-0.09	0.88	0.108	0.247
Newborn birth weight, kg	0.53	0.13	0.94	0.009	0.068	0.66	0.22	1.10	0.003	0.018	0.44	0.02	0.86	0.042	0.122	0.31	-0.17	0.79	0.202	0.355
Maternal BMI, kg/m^2	0.02	-0.03	0.06	0.443	0.673	0.02	-0.03	0.06	0.530	0.800	-0.01	-0.05	0.04	0.717	0.932	0.00	-0.05	0.05	0.976	0.976
Paternal BMI, kg/m^2	-0.01	-0.07	0.05	0.710	0.892	-0.03	-0.10	0.04	0.352	0.936	-0.04	-0.09	0.02	0.225	0.945	-0.04	-0.10	0.03	0.273	0.867
Maternal age, years	-0.03	-0.08	0.01	0.183	0.489	-0.03	-0.10	0.04	0.412	0.666	-0.03	-0.08	0.02	0.200	0.411	-0.01	-0.08	0.05	0.653	0.995
Paternal age, years	-0.03	-0.07	0.01	0.158	0.487	-0.01	-0.08	0.05	0.673	0.902	-0.04	-0.07	-0.01	0.022	0.089	-0.03	-0.08	0.01	0.123	0.331
Maternal smoking, yes vs no	-0.28	-1.09	0.52	0.490	0.668	-0.39	-1.29	0.50	0.391	0.664	-0.04	-0.88	0.80	0.920	0.961	0.64	-0.40	1.69	0.229	0.458
Paternal smoking, yes vs no	0.09	-0.46	0.64	0.746	0.932	0.23	-0.40	0.86	0.474	0.952	-0.49	-1.11	0.13	0.121	0.483	-0.45	-1.16	0.26	0.211	0.633
**n**						364										283				
**S LDL P**																				
Maternal metabolite, nmol/l	0.00	0.00	0.01	0.125	0.259	0.00	0.00	0.01	0.162	0.395	0.00	0.00	0.01	0.594	0.787	0.00	0.00	0.01	0.731	0.801
Paternal metabolite, nmol/l	0.00	0.00	0.01	0.403	0.830	0.00	0.00	0.01	0.254	0.856	0.00	0.00	0.00	0.530	0.789	0.00	0.00	0.00	0.697	0.827
Newborn sex, females vs males	0.44	0.02	0.85	0.040	0.121	0.60	0.14	1.05	0.011	0.041	0.43	-0.02	0.88	0.062	0.493	0.53	0.05	1.02	0.032	0.210
Newborn birth weight, kg	0.46	0.06	0.85	0.024	0.091	0.55	0.12	0.98	0.012	0.048	0.16	-0.25	0.57	0.450	0.600	0.25	-0.22	0.72	0.302	0.450
Maternal BMI, kg/m^2	0.03	-0.01	0.07	0.190	0.450	0.03	-0.02	0.08	0.248	0.547	0.00	-0.05	0.05	0.978	0.994	-0.01	-0.06	0.05	0.753	0.945
Paternal BMI, kg/m^2	0.00	-0.06	0.06	0.951	0.994	-0.03	-0.10	0.04	0.396	0.936	-0.02	-0.08	0.04	0.468	0.945	-0.02	-0.09	0.04	0.450	0.867
Maternal age, years	-0.03	-0.08	0.01	0.153	0.468	-0.05	-0.12	0.02	0.154	0.468	-0.02	-0.07	0.03	0.397	0.537	0.00	-0.06	0.06	0.989	0.995
Paternal age, years	-0.03	-0.07	0.01	0.181	0.487	-0.01	-0.07	0.06	0.870	0.912	-0.05	-0.08	-0.01	0.006	0.063	-0.06	-0.11	-0.02	0.005	0.108
Maternal smoking, yes vs no	-0.28	-1.08	0.52	0.490	0.668	-0.36	-1.26	0.53	0.425	0.664	0.22	-0.63	1.07	0.610	0.858	0.91	-0.17	1.99	0.097	0.431
Paternal smoking, yes vs no	0.00	-0.55	0.55	0.990	0.990	0.06	-0.58	0.69	0.863	0.952	-0.18	-0.80	0.44	0.562	0.765	-0.15	-0.87	0.57	0.686	0.899
**n**						364										283				
**XL HDL P**																				
Maternal metabolite, µmol/l	0.29	-0.70	1.27	0.567	0.698	0.32	-0.76	1.41	0.560	0.797	1.51	0.27	2.76	0.017	0.081	1.61	0.23	2.99	0.022	0.158
Paternal metabolite, µmol/l	0.68	-0.97	2.33	0.421	0.830	1.15	-0.64	2.95	0.208	0.803	-1.15	-2.88	0.58	0.192	0.440	-1.72	-3.68	0.23	0.084	0.298
Newborn sex, females vs males	0.59	0.17	1.01	0.006	0.026	0.66	0.22	1.10	0.004	0.015	0.10	-0.35	0.56	0.659	0.766	0.23	-0.27	0.73	0.360	0.504
Newborn birth weight, kg	0.21	-0.17	0.58	0.277	0.475	0.37	-0.04	0.77	0.075	0.168	0.32	-0.11	0.74	0.145	0.251	0.43	-0.06	0.93	0.085	0.217
Maternal BMI, kg/m^2	-0.01	-0.05	0.03	0.517	0.697	0.00	-0.05	0.04	0.839	0.932	-0.01	-0.06	0.04	0.736	0.932	-0.01	-0.06	0.05	0.849	0.970
Paternal BMI, kg/m^2	-0.04	-0.10	0.02	0.200	0.822	-0.04	-0.10	0.03	0.245	0.936	-0.02	-0.08	0.04	0.451	0.945	0.00	-0.07	0.06	0.896	0.942
Maternal age, years	-0.04	-0.09	0.01	0.090	0.321	-0.05	-0.11	0.02	0.192	0.555	0.02	-0.03	0.07	0.478	0.588	0.00	-0.06	0.07	0.894	0.995
Paternal age, years	-0.03	-0.07	0.01	0.145	0.487	0.00	-0.06	0.06	0.967	0.967	-0.01	-0.05	0.02	0.536	0.686	-0.01	-0.06	0.03	0.547	0.778
Maternal smoking, yes vs no	0.15	-0.63	0.92	0.710	0.858	0.09	-0.77	0.94	0.838	0.909	-1.05	-1.99	-0.10	0.029	0.265	-0.85	-2.02	0.31	0.149	0.431
Paternal smoking, yes vs no	0.16	-0.38	0.69	0.572	0.877	0.15	-0.46	0.77	0.624	0.952	-0.73	-1.39	-0.07	0.031	0.392	-0.72	-1.47	0.04	0.065	0.581
**n**						364										283				
**L HDL P**																				
Maternal metabolite, µmol/l	0.03	-0.35	0.41	0.879	0.893	0.04	-0.38	0.45	0.868	0.934	0.44	-0.06	0.94	0.085	0.217	0.42	-0.12	0.97	0.127	0.475
Paternal metabolite, µmol/l	0.37	-0.25	1.00	0.244	0.830	0.34	-0.34	1.03	0.327	0.955	0.04	-0.77	0.85	0.916	0.986	0.18	-0.71	1.06	0.692	0.827

Newborn sex, females vs males	0.25	-0.15	0.65	0.224	0.333	0.33	-0.10	0.75	0.134	0.226	0.12	-0.34	0.57	0.619	0.762	0.29	-0.21	0.78	0.254	0.394
Newborn birth weight, kg	0.34	-0.04	0.72	0.079	0.195	0.43	0.02	0.83	0.041	0.106	0.58	0.13	1.03	0.011	0.050	0.69	0.18	1.19	0.008	0.037
Maternal BMI, kg/m^2	-0.01	-0.05	0.03	0.583	0.704	-0.01	-0.05	0.04	0.787	0.932	0.00	-0.05	0.05	0.924	0.994	-0.01	-0.07	0.04	0.688	0.940
Paternal BMI, kg/m^2	-0.03	-0.09	0.03	0.272	0.822	-0.02	-0.09	0.04	0.462	0.936	0.00	-0.06	0.06	0.964	0.979	0.03	-0.04	0.09	0.415	0.867
Maternal age, years	-0.03	-0.07	0.02	0.231	0.541	0.00	-0.07	0.07	0.979	0.979	-0.01	-0.07	0.04	0.617	0.707	-0.02	-0.08	0.05	0.592	0.995
Paternal age, years	-0.04	-0.08	0.00	0.060	0.487	-0.04	-0.10	0.02	0.159	0.902	-0.03	-0.07	0.01	0.099	0.228	-0.02	-0.06	0.02	0.363	0.694
Maternal smoking, yes vs no	0.01	-0.76	0.79	0.970	0.970	0.14	-0.71	0.98	0.753	0.884	-0.64	-1.53	0.25	0.158	0.471	-0.31	-1.38	0.76	0.571	0.754
Paternal smoking, yes vs no	-0.12	-0.66	0.42	0.661	0.917	-0.06	-0.67	0.56	0.860	0.952	-0.66	-1.31	0.00	0.049	0.444	-0.63	-1.37	0.10	0.092	0.581
**n**						364										283				
**M HDL P**																				
Maternal metabolite, µmol/l	0.68	0.18	1.18	0.008	0.035	0.64	0.11	1.17	0.019	0.094	0.29	-0.37	0.94	0.389	0.638	0.28	-0.44	1.00	0.445	0.735
Paternal metabolite, µmol/l	0.35	-0.33	1.03	0.319	0.830	0.23	-0.51	0.97	0.540	0.955	0.75	-0.01	1.52	0.053	0.300	1.17	0.27	2.06	0.010	0.211
Newborn sex, females vs males	0.49	0.09	0.89	0.016	0.055	0.50	0.08	0.93	0.021	0.071	0.38	-0.08	0.84	0.102	0.493	0.47	-0.03	0.97	0.066	0.210
Newborn birth weight, kg	0.06	-0.30	0.43	0.734	0.854	0.18	-0.22	0.58	0.367	0.500	0.52	0.08	0.96	0.021	0.071	0.78	0.26	1.30	0.004	0.023
Maternal BMI, kg/m^2	0.00	-0.04	0.04	0.927	0.951	0.01	-0.03	0.06	0.605	0.828	0.00	-0.05	0.05	0.988	0.994	-0.03	-0.09	0.02	0.277	0.764
Paternal BMI, kg/m^2	-0.02	-0.08	0.03	0.400	0.822	-0.03	-0.09	0.04	0.400	0.936	0.04	-0.02	0.10	0.157	0.945	0.06	-0.01	0.13	0.085	0.781
Maternal age, years	-0.01	-0.05	0.04	0.818	0.918	0.01	-0.06	0.08	0.745	0.851	-0.01	-0.06	0.05	0.830	0.869	0.00	-0.07	0.07	0.995	0.995
Paternal age, years	-0.03	-0.07	0.01	0.190	0.487	-0.04	-0.10	0.02	0.210	0.902	-0.02	-0.05	0.01	0.256	0.455	-0.03	-0.07	0.02	0.201	0.496
Maternal smoking, yes vs no	-1.09	-1.97	-0.21	0.015	0.193	-0.88	-1.82	0.07	0.069	0.362	-0.24	-1.10	0.63	0.593	0.858	0.55	-0.53	1.62	0.316	0.579
Paternal smoking, yes vs no	-0.62	-1.18	-0.06	0.029	0.231	-0.35	-0.97	0.28	0.276	0.952	-0.45	-1.09	0.19	0.169	0.561	-0.42	-1.16	0.31	0.258	0.633
**n**						364										283				
**S HDL P**																				
Maternal metabolite, µmol/l	0.33	-0.07	0.72	0.103	0.224	0.37	-0.07	0.82	0.101	0.309	0.63	0.13	1.13	0.014	0.081	0.75	0.19	1.31	0.009	0.084
Paternal metabolite, µmol/l	-0.01	-0.52	0.50	0.970	0.993	-0.11	-0.68	0.45	0.698	0.955	-0.02	-0.51	0.47	0.942	0.986	0.17	-0.39	0.74	0.540	0.786
Newborn sex, females vs males	0.34	-0.06	0.74	0.098	0.224	0.40	-0.03	0.82	0.067	0.133	0.50	0.04	0.96	0.033	0.493	0.53	0.03	1.04	0.037	0.210
Newborn birth weight, kg	0.14	-0.23	0.51	0.458	0.624	0.15	-0.25	0.54	0.465	0.595	0.34	-0.09	0.77	0.123	0.238	0.48	-0.02	0.98	0.060	0.160
Maternal BMI, kg/m^2	0.02	-0.02	0.06	0.328	0.578	0.03	-0.01	0.08	0.157	0.485	-0.02	-0.06	0.03	0.501	0.932	-0.03	-0.09	0.02	0.255	0.764
Paternal BMI, kg/m^2	-0.04	-0.10	0.02	0.193	0.822	-0.05	-0.12	0.01	0.089	0.936	-0.01	-0.07	0.05	0.765	0.945	0.00	-0.07	0.06	0.897	0.942
Maternal age, years	0.01	-0.03	0.06	0.537	0.702	0.03	-0.04	0.10	0.355	0.666	0.01	-0.05	0.06	0.825	0.869	0.00	-0.07	0.06	0.945	0.995
Paternal age, years	-0.01	-0.05	0.03	0.535	0.756	-0.04	-0.09	0.02	0.241	0.902	0.00	-0.04	0.03	0.892	0.975	-0.01	-0.05	0.04	0.715	0.836
Maternal smoking, yes vs no	-0.66	-1.47	0.14	0.105	0.299	-0.42	-1.29	0.46	0.350	0.664	0.77	-0.15	1.68	0.100	0.399	1.26	0.05	2.47	0.041	0.431
Paternal smoking, yes vs no	-0.66	-1.21	-0.11	0.019	0.199	-0.57	-1.19	0.04	0.069	0.952	0.07	-0.56	0.70	0.832	0.903	-0.06	-0.79	0.67	0.879	0.954
**n**						364										283				

Linear mixed model analyses for all cord blood metabolites except lipoprotein subclasses where generalized mixed model analyses were used. Results are presented as estimated regression coefficients (log-odds ratios from generalized mixed model analyses) with 95% confidence intervals (CIs), p-values, and FDR q-values (adjusted for false discovery rate). C, cholesterol; VLDL, very low-density lipoprotein; LDL, low-density lipoprotein; HDL, high-density lipoprotein; TG, triglycerides; PG, phosphoglycerides; Apo, apolipoprotein; FA, fatty acid; PUFA, polyunsaturated fatty acid; MUFA, monounsaturated fatty acid; SFA, saturated fatty acid; LA, linoleic acid; DHA, docosahexaenoic acid; bOHbutyrate, β-hydroxybutyrate; P, particle concentration; IDL, intermediate-density lipoprotein.
