# Supplementary material for: Novel associations between parental and newborn cord blood metabolic profiles in the Norwegian Mother, Father and Child Cohort Study
Source: BMC Med. 2021 Apr 14;19:91. doi: 10.1186/s12916-021-01959-w (PMC8045233; doi:10.1186/s12916-021-01959-w)
Supplement: Supplementary file 5 — Additional file 5. Heatmap of the associations between parental and newborn exposures and newborn metabolites including all subjects. [file 12916_2021_1959_MOESM5_ESM.pdf]

Heatmap of the associations between parental and newborn exposures and newborn metabolites including all subjects.

Results are presented as standardized regression coefficients from linear mixed model analyses, but as log-odds ratios from generalized linear mixed model analyses for lipoprotein particle concentrations. C, cholesterol; VLDL, very low-density lipoprotein; LDL, low-density lipoprotein; HDL, high-density lipoprotein; TG, triglycerides; PG, phosphoglycerides; P cholines, phosphatidylcholines; Apo, apolipoprotein; FA, fatty acid; PUFA, polyunsaturated fatty acid; MUFA, monounsaturated fatty acid; SFA, saturated fatty acid; LA, linoleic acid; DHA, docosahexaenoic acid; bOHbutyrate, β-hydroxybutyrate; glyc, glycoprotein; P, particle concentration; IDL, intermediate-density lipoprotein. Sex, females vs males; smoking, yes vs no; FDR, adjusted for false discovery rate. ***$q_{FDR}<0.001$; **$0.001\leq q_{FDR}<0.05$; *$0.05\leq q_{FDR}<0.10$.

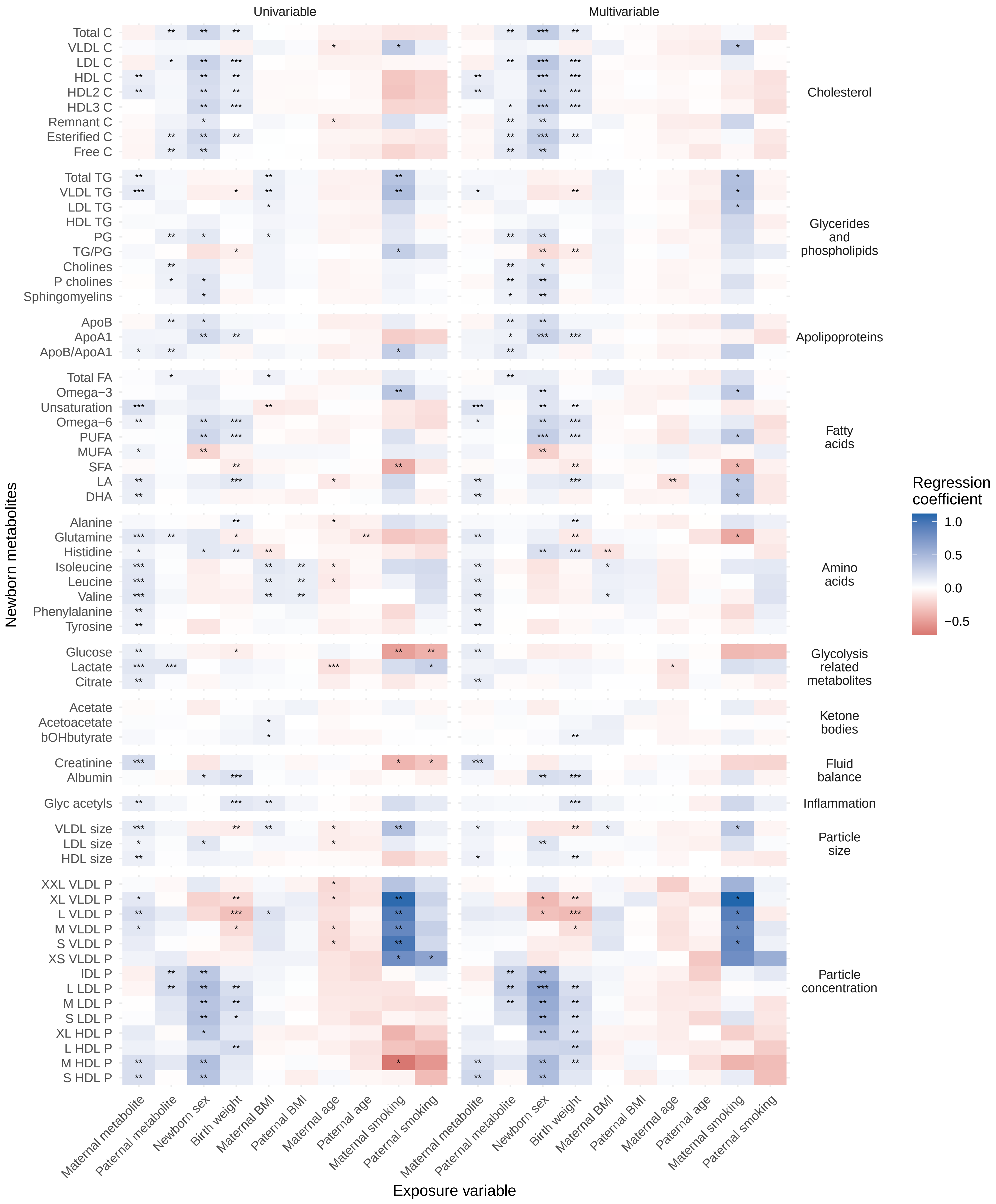
Univariable
Multivariable
Newborn metabolites
Total C
VLDL C
LDL C
HDL C
HDL2 C
HDL3 C
Remnant C
Esterified C
Free C
Cholesterol
Total TG
VLDL TG
LDL TG
HDL TG
PG
TG/PG
Cholines
P cholines
Sphingomyelins
Glycerides and phospholipids
ApoB
ApoA1
ApoB/ApoA1
Apolipoproteins
Total FA
Omega−3
Unsaturation
Omega−6
PUFA
MUFA
SFA
LA
DHA
Fatty acids
Alanine
Glutamine
Histidine
Isoleucine
Leucine
Valine
Phenylalanine
Tyrosine
Amino acids
Glucose
Lactate
Citrate
Glycolysis related metabolites
Acetate
Acetoacetate
bOHbutyrate
Ketone bodies
Creatinine
Albumin
Fluid balance
Glyc acetyls
Inflammation
VLDL size
LDL size
HDL size
Particle size
XXL VLDL P
XL VLDL P
L VLDL P
M VLDL P
S VLDL P
XS VLDL P
IDL P
L LDL P
M LDL P
S LDL P
XL HDL P
L HDL P
M HDL P
S HDL P
Particle concentration
Maternal metabolite
Paternal metabolite
Newborn sex
Birth weight
Maternal BMI
Paternal BMI
Maternal age
Paternal age
Maternal smoking
Paternal smoking
Exposure variable
Regression coefficient
1.0
0.5
0.0
−0.5
